# Synthesis of highly substituted tetrahydroquinolines using ethyl cyanoacetate *via* aza-Michael–Michael addition†

**DOI:** 10.1039/d0ra01264e

**Published:** 2020-04-03

**Authors:** Arunan Palanimuthu, Chinpiao Chen, Gene-Hsian Lee

**Affiliations:** Department of Nursing, Tzu Chi University of Science and Technology Hualien 970 Taiwan chinpiao@ems.tcust.edu.tw +886 3 856 1097 +886 3 857 2158 ext. 2624; Department of Chemistry, National Dong Hwa University Soufeng Hualien 974 Taiwan; Instrumentation Center, College of Science, National Taiwan University Taipei 106 Taiwan

## Abstract

A three-component cascade reaction involving 2-alkenyl aniline, aldehydes, and ethyl cyanoacetate in the presence of DBU to synthesize highly substituted 1,2,3,4-tetrahydroquinolines is reported. The reaction proceeded through the Knoevenagel condensation of ethyl cyanoacetate with aldehydes followed by the aza-Michael–Michael addition with 2-alkenyl anilines to prepare the tetrahydroquinoline scaffolds.

## Abstract 2

A three-component cascade reaction involving 2-alkenyl aniline, aldehydes, and ethyl cyanoacetate in the presence of DBU to synthesize highly substituted 1,2,3,4-tetrahydroquinolines is reported.

## Graphical abstract

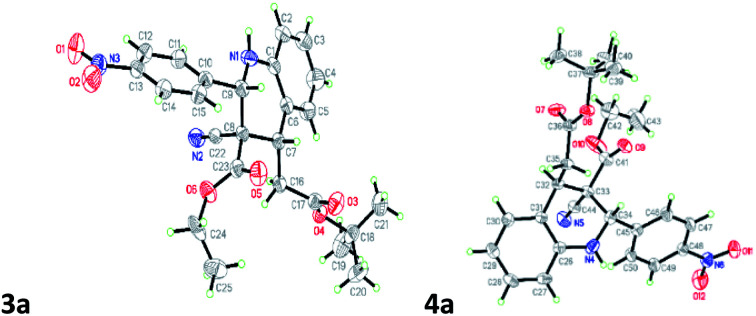

## Introduction

Cascade or tandem reactions continue to be of interest because they offer a rapid and highly effective strategy for the synthesis of bioactive natural products^1–5^ and pharmaceutical agents.^6–16^ Tetrahydroquinolines have been targeted by many research groups because of their abundance in natural products and notable biological activity. Tetrahydroquinoline derivatives are used in pesticides, antioxidants, photosensitizers, and dyes in addition to pharmaceutical applications. Overall, the tetrahydroquinoline family has a wide range of applications and is a key structural motif in pharmaceutical agents; therefore, multiple strategies have been proposed for the synthesis of tetrahydroquinoline derivatives.^17–22^

Cascade reactions are valuable for generating 1,2,3,4-tetrahydroquinoline skeletons with various substitution groups, and many new drugs have been designed on the basis of this process. Bunce *et al.* reported a tandem-reduction-reductive cyclization sequence in one pot of ozonolysis-reduction followed by a reductive amination reaction sequence provided by *N*-methyl-2-substituted-1,2,3,4-tetrahydroquinoline 4-carboxylic esters.^23^ Povaraov performed an acid catalyzed one-pot conversion of *N*-arylimines and electron-rich dienophiles to produce 1,2,3,4-tetrahydroquinoline, which is normally classified as an aza-Diels–Alder or imino Diels–Alder reaction.^24^ Menéndez *et al.* revealed that CAN catalyzed the one-pot diastereoselective synthesis of 4-alkoxy-2-ary-1,2,3,4-tetrahydroquinolines.^25^ Wang reported that earlier Mannich–Michael addition using malononitrile as a nucleophile toward 2-alkenyl substituted imines yielded optically enriched and highly substituted tetrahydroquinolines.^26^ Commercially available, inexpensive ethyl cyanoacetate has seldom been discussed in relation to the synthesis of tetrahydroquinolines.

## Results and discussion

In this paper, it reports the simple one-pot economical preparation of highly substituted tetrahydroquinolines by using 2-alkenyl substituted aniline, aromatic aldehydes, and ethyl cyanoacetate; this method saves time during the workup procedure and purification of intermediates and yields minimal reagent waste.

The DBU plays a dual role in the cascade conversion of the Knoevenagel condensation intermediate as well as in the aza-Michael–Michael addition to prepare 1,2,3,4-tetrahydroquinolines. Thus, the overall conversion was integrated irrespective of the Michael acceptors attached to aniline, and resulted in high diastereoselectivity up to 93 : 7. Initial reaction conditions were tested with *tert*-butyl 2-alkenyl substituted imines (1) and ethyl cyanoacetate with bases including TEA, DIPEA, DABCO, and DBN (Table 1, entries 1–4) in DCM; however no characteristic reactions occurred.^27^ K_2_CO_3_ as a base in DMF and DMSO demonstrated reasonable conversion (Table 1, entries 5 and 6), and it was found that DBU in DCM enabled excellent conversion of (*E*)-*tert*-butyl-3-(2-((*E*)-4-nitrobenzylideneamino)phenyl)acrylate into tetrahydroquinolines 3a/4a at room temperature (Table 1, entries 10 and 11, 95%, racemate). DBU was deemed superior to the other bases.

**Table 1:** Ethyl cyanoacetate as nucleophile

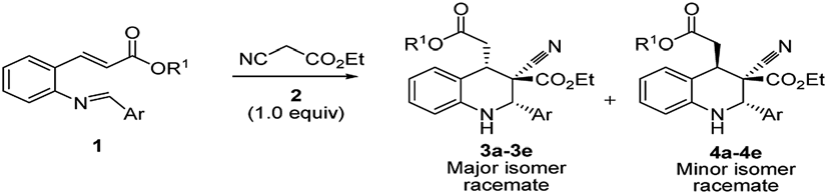							
Entry	R^1^	Ar^a^	Base (mol%)	Solvent	*T* (h)	Yield^b^ (%)	Ratio 3/4^c^
1	*t*-Bu	4-NO_2_Ph (3a/4a)	DABCO (100)	DCM	24	—	—
2	*t*-Bu	4-NO_2_Ph (3a/4a)	DIPEA (200)	DCM	24	—	—
3	*t*-Bu	4-NO_2_Ph (3a/4a)	DBN (100)	DCM	12	—	—
4	*t*-Bu	4-NO_2_Ph (3a/4a)	TEA (100)	DCM	24	—	—
5	*t*-Bu	4-NO_2_Ph (3a/4a)	K_2_CO_3_ (50)	DMF	12	74	62 : 38
6	*t*-Bu	4-NO_2_Ph (3a/4a)	K_2_CO_3_ (50)	DMSO	12	45	75 : 25
7	*t*-Bu	4-NO_2_Ph (3a/4a)	DBU (50)	DMF	5	79	70 : 30
8	*t*-Bu	4-NO_2_Ph (3a/4a)	DBU (50)	MeOH	8	31	51 : 49
9	*t*-Bu	4-NO_2_Ph (3a/4a)	DBU (50)	THF	8	45	54 : 46
10	*t*-Bu	4-NO_2_Ph (3a/4a)	DBU (200)	DCM	3	95	65 : 35
11	*t*-Bu	4-NO_2_Ph (3a/4a)	DBU (50)	DCM	3	95	67 : 33
12^d^	*t*-Bu	4-NO_2_Ph (3a/4a)	DBU (50)	DCM	10	62	52 : 48
13	*t*-Bu	4-OMePh (3b/4b)	DBU (50)	DCM	12	—	—
14	*t*-Bu	Ph (3c/4c)	DBU (50)	DCM	12	—	—
15	Me	4-NO_2_Ph (3d/4d)	DBU (50)	DCM	2	96	74 : 26
16	Me	2-OMePh (3e/4e)	DBU (50)	DCM	12	67	85 : 15
17	Me	3,5-diOMePh (3f/4f)	DBU (50)	DCM	48	—	—
18	Me	4-NO_2_Ph (3d/4d)	TEA (50)	DCM	12	—	—
19	Me	4-NO_2_Ph (3d/4d)	DIPEA (100)	DCM	12	—	—

a All reactions were performed in 30 to 50 mg scale.

b Yield of isolated product is a mixture of diastereomers after column chromatography.

c Determined by ^1^H NMR analysis of crude reaction mixture.

d Reactions were completed at −10 °C to rt, 10 h.

**Fig. 1:**
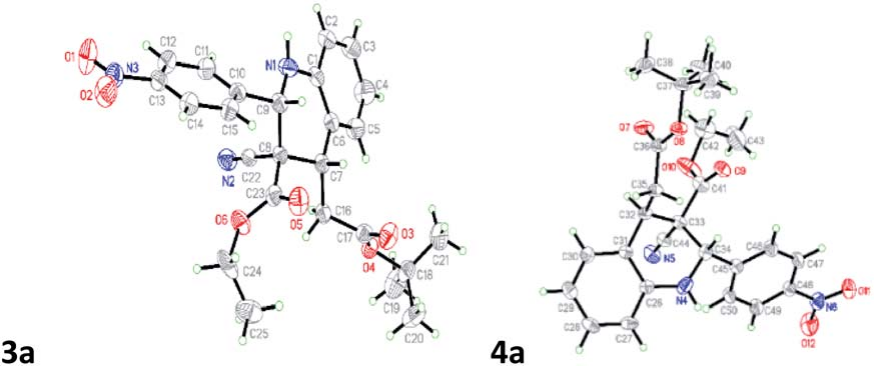
X-ray studies confirmed the relative isomeric structures of 3a (CCDC 1834300) and 4a (CCDC 1834305).

The 3a/4a isomers were separated through column chromatography, were recrystallized in DCM, and underwent X-ray analysis (Fig. 1) to facilitate understanding of the relative configuration of the diastereomers. The groups of 1,3-*cis*-tetrahydroquinoline 3a (major isomer) with the distorted chair configuration of 4-NO_2_Ph, –CH_2_CO_2_-^*t*^Bu preferred the same side of the ring; the opposite was observed for 4a (alternative, both hydrogens were 1,3 *cis* in the major diastereomer and in its opposite). To further evaluate diastereoselectivity, we measured the reaction temperature and catalyst loading; however, the results revealed a poor yield and no evident improvement in diastereoselectivity (Table 1, entries 10 and 12).

Further investigations were conducted using solvents such as MeOH, THF, and DMF (Table 1, entries 7–9) with DBU as a base, but no significant improvements in diastereomeric ratio (dr) or yield were observed.

The combination of DCM and DBU was preferable to the other solvents. The mixture of diastereomers was inevitable, and it further experimented with the versatility of the reaction through the cascade addition. Methyl 2-alkenyl-substituted imine (Table 1, entries 15 and 16) yielded products with improved diastereoselectivity.

**Scheme 1:**
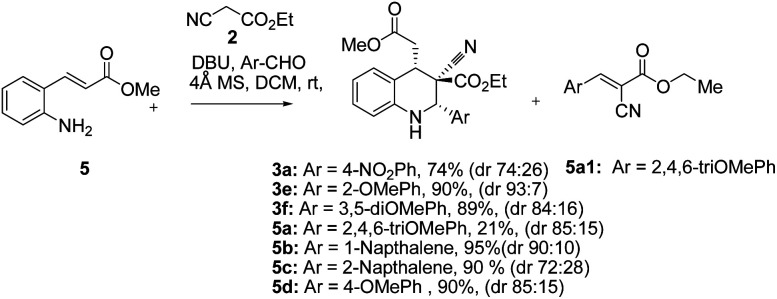
One pot-three component cascade reaction.

The synthesis and purification of Schiff bases were tedious in many cases; thus a complete tetrahydroquinoline conversion was attempted in a one-pot reaction. Reacting ethyl cyanoacetate, (*E*)-methyl 3-(2-aminophenyl)acrylate (5), and substituted aromatic aldehydes with DBU yielded 1,2,3,4-substituted tetrahydroquinolines effectively (Scheme 1).^28^ Electron rich aldehydes resulted in better conversion compared with the other aldehydes. The corresponding product 3e of 2-anisaldehyde demonstrated improved diastereoselectivity compared with the other substituted benzaldehydes (Scheme 1).

Tetrahydroquinolines obtained from 1-naphthaldehyde demonstrated improve yield and diastereoselectivity compared with those obtained from 2-naphthadehyde (5b and 5cScheme 1). Unexpectedly, when DBU was used as the base, the synthesis of 3f (Scheme 1), was unsuccessful after the corresponding imine reacted with ethyl cyanoacetate (Table 1, entry 17). In addition, the synthesis of 5a (Scheme 1) produced a low yield, and we managed to isolate the intermediate 5a1, which altered our understanding regarding the mechanistic pathway of the cascade reaction and verified the formation of 1,2,3,4-tetrahydroquinolines through a Knoevenagel-condensation intermediate.

**Scheme 2:**
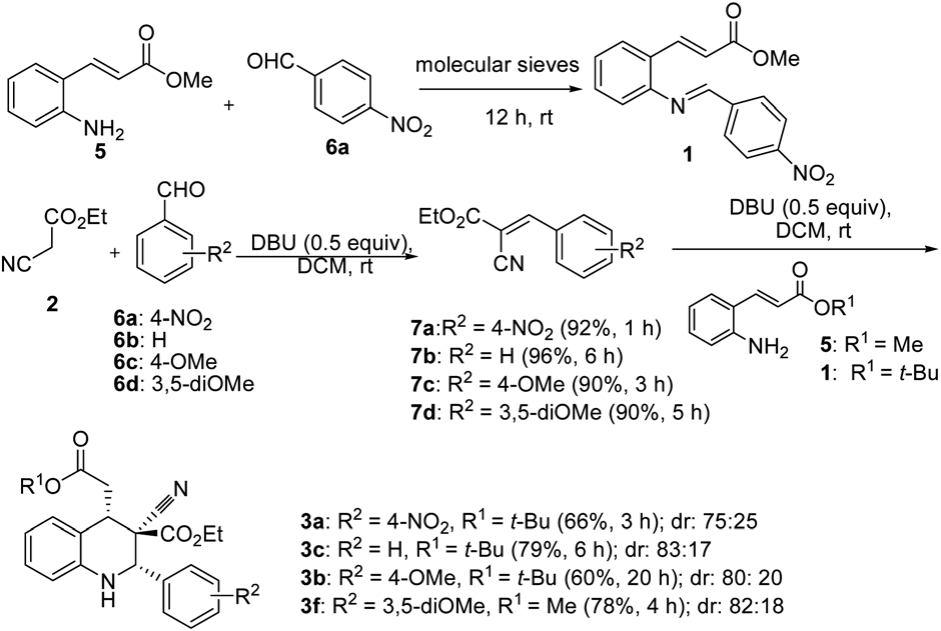
Two component approach *via* Knoevenagel intermediate.

Two control experiments were performed and monitored by TLC. (*E*)-Methyl 3-(2-aminophenyl)acrylate (5) and *p*-nitrobenzaldehyde (6a) in CH_2_Cl_2_, combined with the application of molecular sieves (4 Å), were used to synthesize the corresponding imine. This reaction solution was stirred at room temperature for 1 h and monitored by TLC, which revealed extremely poor conversion. By contrast, ethyl cyanoacetate (2) reacted readily with *p*-nitrobenzaldehyde (6a) in the presence of DBU to produce a Knoevenagel condensation product (7a).^29^ Similarly, the other intermediates (7b, 7c, and 7d) were synthesized under optimized conditions (Scheme 2). Electron-rich aldehydes (2-anisaldehyde and 4-anisaldehyde) were converted to imine (1, Table 1) at high temperatures (110 °C) by using toluene as a solvent to describe the formation of 1,2,3,4-tetrahydroquinolines at room temperature through Knoevenagel-condensation intermediate. Tetrahydroquinolines (3c, 3b, and 3f) synthesis was successful when Knoevenagel intermediates (7b, 7c, and 7d) were used and mediated by DBU in a two-component approach (Scheme 2).

**Fig. 2:**
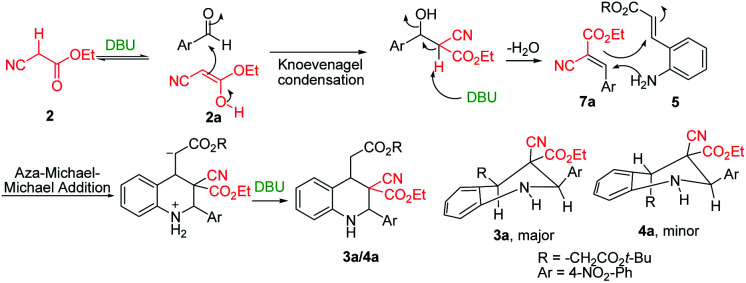
Plausible mechanism.

After the initial formation of enol intermediate 2a, the intermediate reacted with aldehyde to produce an aldol product that subsequently endured base-induced elimination to form 7a (Fig. 2). Reactions between Schiff base's and enol intermediate 2a (Mannich reaction) had failed in earlier experiments (Table 1, entries 13, 14, and 17) because the imines were mostly inert, and thus unable to react with ethyl cyanoacetate. It understand from the crystal structures 3a/4a (Fig. 1) that the initial aza-Michael addition to a Knoevenagel intermediate considerably increased the diastereoselectivity whereas subsequent Michael addition to α,β-unsaturated esters yielded a diastereomeric mixture. Thus, for the synthesis of 1,2,3,4-tetrahydroquinoline, it propose a plausible mechanism with a Knoevenagel intermediate that favours cascade transition through the aza-Michael–Michael addition.^30^

To determine the effective substrate scope of the reaction, it was reviewed systematic studies performed under optimized conditions (Table 2). In this study, 2-alkenyl-4-chloroanilines were efficiently converted to their corresponding tetrahydroquinolines 9a–9g (Table 2, entries 1–7). Regardless of the groups (X = Cl, H or CO_2_Me) present at 2-alkenylaniline, the yields of the tetrahydroquinolines primarily varied according to the reactivity of the aldehydes. Heteroaromatic aldehydes underwent one-pot conversion into 1,2,3,4-tetrahydroquinolines (9e–9g) with moderate yields (Table 2, entries 5–7). Aromatic aldehydes under the same conditions produced 9a, 9j, and 9n (Table 2, entries 1, 10, and 14) and demonstrated excellent yields compared with the other heteroaromatic aldehydes (Table 2, entries 5–7). In the synthesis of tetrahydroquinoline 9a up to 93 : 7, *o*-anisaldehyde exhibited the highest diastereoselectivity (Table 2, entry 1).

**Table 2:** Substrate scope

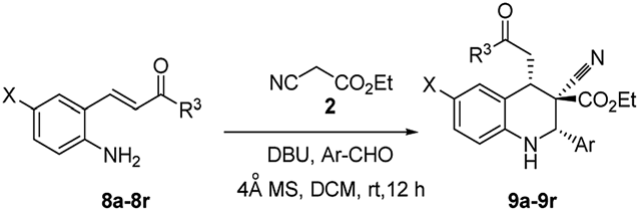					
Entry	Ar^a^	X	R^3^	Yield^b^ (%)	dr^c^
1	2-OMePh (9a)	Cl	OMe	88	93 : 7
2	3,5-diOMePh (9b)	Cl	OMe	89	81 : 19
3	2-Naphthyl (9c)	Cl	OMe	92	79 : 21
4	1-Naphthyl (9d)	Cl	OMe	90	91 : 9
5	2-Pyridyl (9e)	Cl	OMe	54	76 : 24
6	3-Thiophenyl (9f)	Cl	OMe	65	76 : 24
7	2-Thiophenyl (9g)	Cl	OMe	63	72 : 28
8	2-Naphthyl (9h)	CO_2_Me	OMe	85	70 : 30
9	1-Naphthyl (9i)	CO_2_Me	OMe	88	84 : 16
10	2-OMePh (9j)	CO_2_Me	OMe	91	90 : 10
11	3,5-diOMePh (9k)	CO_2_Me	OMe	86	80 : 20
12	2-Naphthyl (9l)	H	Ph	95	75 : 25
13	2-OMePh (9m)	H	Ph	90	90 : 10
14	1-Naphthyl (9n)	H	Ph	92	90 : 10
15	2-Thiophenyl (9o)	H	Ph	84	72 : 28
16	3,5-diOMePh (9p)	H	Ph	81	79 : 21
17	4-NO_2_Ph (9q)	H	Ph	83	70 : 30
18	2,4,6-triOMePh (9r)	H	Ph	79	83 : 17

a All reactions were performed in 50 mg scale at room temperature.

b Yield of isolated product was a mixture of diastereomers after column chromatography.

c Determined by ^1^H NMR analysis of the crude reaction mixture.

Naphthaldehydes (Table 2, entries 3 and 4) were converted into 1,2,3,4-tetrahydroquinolines (9c and 9d) under optimized conditions, and 5-methoxycarbonylaniline analogues were converted into their corresponding tetrahydroquinolines (9h–9k) with good to moderate yields (Table 2, entries 8–11). In addition to examining the versatility of the reaction toward Michael acceptor α,β-unsaturated esters (Schemes 1 and 2), it was also examined that of the reaction toward α,β-unsaturated phenyl ketones in tetrahydroquinoline synthesis; the results demonstrated high efficiency. In one-pot, 2-amino substituted chalcones were converted into 1,2,3,4-tetrahydroquinolines with good to moderate yield; all yields were superior to those of the other analogues (Table 2, entries 12–18). No major differences in diastereoselectivity were caused by α,β-unsaturated phenyl ketones (Table 2, entries 13 and 14); however, better yields were obtained with high diastereoselectivity upto 90 : 10. The stronger electron-withdrawing phenyl ketone group accelerated cascade conversion more easily than the other α,β-unsaturated esters. Separation of the diastereomers through column chromatography and preparative TLC failed in most cases; therefore, they were able to triturate the 1,3-*cis* isomer (major) separately from the mixture of diastereomers by using methanol.

## Conclusions

In summary, it was developed a simple DBU mediated cascade process to effectively synthesize a new class of highly substituted 1,2,3,4-tetarhydroquinolines by using ethyl cyanoacetate in one pot. The reaction mechanism was investigated through control experiments, namely three reactions involving Knoevenagel condensation followed by aza-Michael–Michael addition efficiently conducted at the room temperature with simple practicability.

## Experimental section

### General methods

Melting points were recorded using a Yanagimoto Micro Melting Point Apparatus Model-S3 capillary melting point apparatus and are uncorrected. TLC analysis was carried out on silica gel 60 F254 precoated glass sheets and detected under UV light. ^1^H NMR spectra were obtained at 300, 400 or 500 MHz (as indicated), and ^13^C NMR spectra were obtained at 75.5, 100 or 125.6 MHz, using a Bruker NMR spectrometer. Chemical shifts (*δ*) are reported in parts per million (ppm) relative to CDCl_3_ (7.26 and 77.0 ppm), the coupling constants are reported in hertz (Hz) and the multiplicities are indicated as b = broad, s = singlet, d = doublet, dd = doublet of doublet, dt = doublet of triplet, t = triplet, m = multiplet. In each case proton NMR showed the presence of indicated solvent(s). Infrared spectra were recorded using PerkinElmer FT/IR spectrometer. Mass spectra were recorded on a Micromass Platform II or Finnigan/Thermo Quest MAT 95XL spectrometer. All reactions were carried out in anhydrous solvents. CH_2_Cl_2_, DMF, DMSO were distilled from Molecular Sieves. MeOH was distilled from Mg cake. All chemicals and solvents were purchased from Aldrich Chemical Co.

### A typical procedure for synthesis of ethyl 6-chloro-3-cyano-4-(2-methoxy-2-oxoethyl)-2-(2-methoxyphenyl)-1,2,3,4-tetrahydroquinoline-3-carboxylate, 9a

A solution of (*E*)-3-(2-amino-5-chlorophenyl)acrylate (0.24 mmol), ethyl cyanoacetate (0.28 mmol), and 2-methoxybenzaldehyde (0.28 mmol) in CH_2_Cl_2_ (5 mL) with DBU (0.12 mmol) was stirred at room temperature, followed by the addition of molecular sieves (4 Å, 30 mg). The reaction mixture was stirred at room temperature for 12 h under N_2_ atmosphere, and the progress of the reaction was monitored by TLC (eluent: 20% EtOAc in hexane). The crude product was filtered through Celite and washed using CH_2_Cl_2_ (20 mL). The organic solvent was removed by a rotary evaporator under reduced pressure, and the obtained crude product was purified by column chromatography (100–200 mesh silica) using 30% ethyl acetate in hexane as an eluent. The mixture of diastereomers (94 mg, 88%) was stirred in anhydrous methanol, and the white precipitate that appeared was filtered and dried to yield major isomer 9a. Yield: 66.7% (71 mg); white solid; mp 164–166 °C; ^1^H NMR (400 MHz, CDCl_3_) 7.86 (1H, d, *J* = 7.8 Hz), 7.38–7.35 (1H, m), 7.08–7.02 (3H, m), 6.89–6.87 (1H, m), 6.58–6.56 (1H, m), 5.27 (1H, s), 4.40–4.38 (1H, m), 4.15 (1H, s), 4.03–3.96 (2H, m), 3.86 (1H, s), 3.80 (3H, b), 3.78 (3H, b), 2.93 (1H, dd, *J* = 7.9, 17.1 Hz), 2.71 (1H, dd, *J* = 3.4, 17.0 Hz), 0.97 (3H, t, *J* = 7.2 Hz); ^13^C NMR (100 MHz, CDCl_3_) 172.0, 166.2, 157.1, 141.7, 130.5, 128.1, 127.3, 124.4, 123.8, 122.0, 121.3, 116.3, 115.8, 110.5, 62.9, 55.5, 54.2, 53.6, 52.5, 13.5; FT-IR (KBr, *ν̃*) 3389, 2952, 2225, 1722, 1602, 1492, 1465, 1368, 1296, 1258, 1170, 1051, 1023, 862, 824, 755 cm^−1^; LRMS-EI^+^ (*m*/*z*) 465.40 ([M + Na]^+^100), 443.60 (43.69), 444.70 (16.67), 445.66 (13.54). HRMS-TOF-ES^+^ (*m*/*z*) [M + H]^+^ calcd for C_23_H_24_ClN_2_O_5_ 443.1374, found 443.1373.

### Ethyl 4-(2-(*tert*-butoxy)-2-oxoethyl)-3-cyano-2-(4-nitrophenyl)-1,2,3,4-tetrahydroquinoline-3-carboxylate (major isomer), 3a

Yellow solid; mp 198–200 °C; ^1^H NMR (300 MHz, CDCl_3_) 8.26 (2H, d, *J* = 8 8 Hz), 7.80 (2H, d, *J* = 8.8 Hz), 7.20–7.01 (2H, m), 6.85 (1H, dd, *J* = 7.7, 7.7 Hz), 6.68 (1H, d, *J* = 8.0 Hz), 4.87 (1H, s), 4.38–4.01 (3H, m), 2.88 (1H, dd, *J* = 7.8, 17.2 Hz), 2.65 (1H, dd, *J* = 3.8, 17.3 Hz), 1.60–1.36 (9H, m), 1.05 (3H, t, *J* = 7.1 Hz); ^13^C NMR (75.5 MHz, CDCl_3_) 170.7, 166.5, 148.8, 143.5, 142.1, 129.3, 128.3, 127.5, 123.9, 120.6, 120.0, 115.3, 114.9, 81.7, 63.3, 61.4, 55.4, 41.2, 38.7, 41.2, 38.7, 28.0, 13.8; FT-IR (KBr, *ν̃*) 3377, 2980, 2923, 2247, 1734, 1608, 1524, 1488, 1368, 1349, 1296, 1248, 1152, 1040, 858, 748 cm^−1^; LRMS-EI (*m*/*z*) 464.13 ([M − H]^+^, 100), 464.13 (28); HRMS-TOF-ES (*m*/*z*) [M − H]^+^ calcd for C_25_H_26_N_3_O_6_ 464.1822, found 464.1825.

### Ethyl 4-(2-(*tert*-butoxy)-2-oxoethyl)-3-cyano-2-(4-nitrophenyl)-1,2,3,4-tetrahydroquinoline-3-carboxylate (minor isomer), 4a

Yellow solid; mp 188–189 °C; ^1^H NMR (300 MHz, CDCl_3_) 8.26–8.21 (2H, d, *J* = 8.8 Hz), 7.91–7.86 (2H, d, *J* = 8.8 Hz), 7.22–7.07 (2H, m), 6.83–6.76 (1H, m), 6.65 (1H, d, *J* = 8.0 Hz), 4.97 (1H, s), 4.22–3.97 (3H, m), 2.91 (1H, dd, *J* = 7.7, 17.3 Hz), 2.57 (1H, dd, *J* = 5.2, 17.3 Hz), 1.46 (9H, m), 1.26 (1H, s), 1.15 (3H, t, *J* = 7.1 Hz); ^13^C NMR (75 MHz, CDCl_3_) 170.0, 166.0, 148.6, 144.4, 140.8, 130.3, 129.5, 128.8, 123.6, 119.7, 119.3, 116.5, 81.5, 63.2, 55.3, 50.7, 41.3, 40.8, 28.1, 13.7; FT-IR (KBr, *ν̃*) 3392, 2918, 2319, 1739, 1605, 1525, 1490, 1349, 1246, 1155, 1041, 848 cm^−1^; LRMS-EI (*m*/*z*) 464.18 ([M − H]^+^, 100), 464.13 (28); HRMS-TOF-ES (*m*/*z*) [M − H]^+^ calcd for C_25_H_26_N_3_O_6_ 464.1822, found 464.1821.

### Ethyl 4-(2-(*tert*-butoxy)-2-oxoethyl)-3-cyano-2-(4-methoxyphenyl)-1,2,3,4-tetrahydroquinoline-3-carboxylate, 3b

White solid; mp 138–140 °C; ^1^H NMR (400 MHz, CDCl_3_) 7.51–7.49 (2H, d, *J* = 8.6 Hz), 7.11–7.05 (2H, m), 6.92–6.90 (2H, d, *J* = 8.6 Hz), 6.78 (1H, t, *J* = 7.5 Hz), 6.64–6.60 (1H, m), 4.68 (1H, s), 4.26 (1H, dd, *J* = 2.7, 7.9 Hz), 4.06 (2H, q, *J* = 7.0 Hz), 3.82 (3H, s), 2.85 (1H, dd, *J* = 8.2, 17.0 Hz), 2.62 (1H, dd, *J* = 3.3, 16.9 Hz), 0.86 (1H, t, *J* = 7.1 Hz); ^13^C NMR (100 MHz, CDCl_3_) 170.9, 167.0, 160.6, 142.8, 129.1, 128.4, 128.1, 127.4, 120.49, 119.11, 115.67, 114.91, 114.15, 81.48, 62.86, 61.69, 55.6, 55.4, 41.1, 38.7, 28.0, 13.8; FT-IR (KBr, *ν̃*) 2979, 2247, 1738, 1610, 1585, 1514, 1485, 1368, 1299, 1152, 1034, 839, 748 cm^−1^; LRMS-EI^+^ (*m*/*z*) 473.24 ([M + Na]^+^, 100), 451.36 (13.48), 459.34 (69.06); HRMS-TOF-ES^+^ (*m*/*z*) [M + H]^+^ calcd for C_26_H_30_N_2_NaO_5_ 473.2052, found 473.2054.

### Ethyl4-(2-(*tert*-butoxy)-2-oxoethyl)-3-cyano-2-phenyl-1,2,3,4-tetrahydroquinoline-3-carboxylate, 3c

White solid; mp 146–148 °C; ^1^H NMR (400 MHz, CDCl_3_) 7.59–7.57 (2H, m), 7.41–7.40 (3H, m), 7.12–7.06 (2H, m), 6.80 (1H, t, *J* = 7.5 Hz), 6.64 (1H, d, *J* = 7.7 Hz), 4.73 (1H, s), 4.29 (1H, dd, *J* = 2.9, 7.8 Hz), 4.12–3.94 (2H, m), 2.90–2.82 (1H, dd, *J* = 8.24, 16.9 Hz), 2.63 (1H, dd, *J* = 3.3, 16.9 Hz), 1.51 (9H, s), 1.00–0.97 (1H, d, *J* = 7.1 Hz); ^13^C NMR (75 MHz, CDCl_3_) 170.9, 166.9, 142.7, 136.4, 129.7, 128.9, 128.1, 127.9, 127.5, 120.5, 119.2, 115.5, 115.0, 81.5, 62.9, 62.3, 55.4, 41.2, 38.8, 28.0, 13.7; FT-IR (KBr, *ν̃*) 2978, 2918, 2247, 1731, 1605, 1586, 1487, 1368, 1247, 1152, 988, 848, 747, 700 cm^−1^; LRMS-EI^+^ (*m*/*z*) 443.19 ([M + Na]^+^, 100), 421.19 (25.05); HRMS-TOF-ES^+^ (*m*/*z*) [M + H]^+^ calcd for C_25_H_28_N_2_NaO_4_ 443.1947, found 443.1947.

### Ethyl 3-cyano-4-(2-methoxy-2-oxoethyl)-2-(4-nitrophenyl)-1,2,3,4-tetrahydroquinoline-3-carboxylate, 3d

Yellow solid; mp 81–83 °C; ^1^H NMR (400 MHz, CDCl_3_) 8.29–8.24 (2H, m), 7.83–7.78 (2H, m), 7.17–7.11 (1H, m), 7.07–7.02 (1H, m), 6.88–6.82 (1H, m), 6.7–6.66 (1H, m), 4.88 (1H, s), 4.35 (1H, m), 4.24 (1H, s), 4.11–4.02 (2H, m), 3.78 (3H, s), 3.00 (1H, dd, *J* = 7.5, 17.1 Hz), 2.78–2.70 (1H, m), 1.04 (3H, t, *J* = 7.0 Hz); ^13^C NMR (100 MHz, CDCl_3_) 172.0, 166.5, 148.8, 143.4, 142.1, 129.3, 128.5, 127.3, 124.0, 120.0, 115.4, 114.8, 63.4, 61.3, 55.2, 52.5, 41.1, 37.1, 13.8; FT-IR (KBr, *ν̃*) 3375, 2955, 2247, 1739, 1608, 1524, 1488, 1350, 1295, 1247, 1247, 1161, 1041, 858, 750, 699 cm^−1^; LRMS-EI^+^ (*m*/*z*) 424.45 ([M + H]^+^, 100), 276.37 (4); HRMS-TOF-ES^+^ (*m*/*z*) [M + H]^+^ calcd for C_22_H_22_N_3_O_6_ 424.1509, found 424.1508.

### Ethyl 3-cyano-4-(2-methoxy-2-oxoethyl)-2-(4-nitrophenyl)-1,2,3,4-tetrahydroquinoline-3-carboxylate, 4d

Yellow solid; mp 70–72 °C; ^1^H NMR (400 MHz, CDCl_3_) 8.23 (2H, d, *J* = 8.8 Hz), 7.88 (2H, d, *J* = 8.8 Hz), 7.22–7.07 (2H, m), 6.81 (1H, dd, *J* = 7.5, 7.5 Hz), 6.67 (1H, d, *J* = 7.7 Hz), 4.98 (1H, s), 4.21 (1H, s), 4.18–3.99 (3H, m), 3.69 (3H, s), 3.01 (1H, dd, *J* = 7.9, 17.4 Hz), 2.69 (1H, dd, *J* = 5.1, 17.2 Hz), 1.16 (3H, t, *J* = 7.2 Hz); ^13^C NMR (100 MHz, CDCl_3_) 171.2, 166.0, 148.6, 144.2, 140.9, 130.2, 129.4, 129.0, 123.7, 119.3, 119.29, 116.3, 114.7, 63.5, 55.2, 52.1, 50.7, 41.46, 39.7, 13.7; FT-IR (KBr, *ν̃*): 3391, 2927, 2247, 1737, 1609, 1524, 1495, 1349, 1241, 1173.1, 1040.1, 857.0, 751.9 cm^−1^; LRMS-EI^+^ (*m*/*z*) 424.45 ([M + H]^+^, 100), 276.37 (4); HRMS-TOF-ES^+^ (*m*/*z*) [M + H]^+^ calcd for C_22_H_22_N_3_O_6_ 424.1509, found 424.1508.

### Ethyl 3-cyano-4-(2-methoxy-2-oxoethyl)-2-(2-methoxyphenyl)-1,2,3,4-tetrahydroquinoline-3-carboxylate, 3e

White solid; mp 118–120 °C; ^1^H NMR (400 MHz, CDCl_3_) 7.90 (1H, d, *J* = 6.8 Hz), 7.37–7.33 (1H, m), 7.11–7.03 (3H, m), 6.89–6.87 (1H, d, *J* = 8.0 Hz), 6.81–6.77 (1H, m), 6.63–6.62 (1H, d, *J* = 8.0 Hz), 5.30 (1H, s), 4.43 (1H, dd, *J* = 2.9, 7.7 Hz), 4.13 (1H, m), 4.02–3.98 (2H, m), 3.78 (6H, b), 2.96 (1H, dd, *J* = 8.0, 14.0 Hz), 2.72 (1H, dd, *J* = 3.3, 16.9 Hz), 0.98 (3H, t, *J* = 7.0 Hz); ^13^C NMR (100 MHz, CDCl_3_) 172.3, 166.5, 157.1, 143.1, 130.4, 128.2, 128.0, 127.3, 124.9, 121.3, 120.5, 119.2, 116., 116.1, 115.2, 110.5, 62.7, 55.5, 54.2, 54.1, 52.3, 41.1, 37.4, 13.5; FT-IR (KBr, *ν̃*) 3381, 2953, 2840, 2236, 1737, 1606, 1492, 1439, 1368, 1291, 1249, 1161, 1025, 857, 755 cm^−1^; LRMS-EI^+^ (*m*/*z*) 431.17 ([M + Na]^+^, 100), 409.30 ([M + H]^+^, 38.94); HRMS-TOF-ES^+^ (*m*/*z*) [M + H]^+^ calcd for C_23_H_25_N_2_O_5_ 409.1763, found 409.1762.

### Ethyl 3-cyano-2-(3,5-dimethoxyphenyl)-4-(2-methoxy-2-oxoethyl)-1,2,3,4-tetrahydroquinoline-3-carboxylate, 3f

White powder; mp 156–158 °C; ^1^H NMR (400 MHz, CDCl_3_) 7.11 (1H, t, *J* = 7.5 Hz), 7.03–7.01 (1H, d, *J* = 7.4 Hz), 6.79 (1H, t, *J* = 7.5 Hz), 6.73–6.72 (2H, m), 6.66–6.64 (1H, d, *J* = 8.0 Hz), 6.49–6.47 (1H, m), 4.65 (1H, s), 4.33 (1H, dd, *J* = 2.9, 7.7 Hz), 4.25 (1H, s), 4.14–4.02 (2H, m), 3.79 (6H, s), 3.78 (3H, s), 2.97 (1H, dd, *J* = 7.9, 17.1 Hz), 2.71 (1H, dd, *J* = 3.5, 17.1 Hz), 1.05 (3H, t, *J* = 7.2 Hz); ^13^C NMR (100 MHz, CDCl_3_) 172.1, 166.96, 161.1, 142.5, 138.5, 128.2, 127.2, 120.6, 119.31, 115.5, 115.0, 105.7, 101.85, 63.0, 62.3, 55.5, 55.2, 52.3, 41.1, 37.1, 13.7; FT-IR (KBr, *ν̃*) 3380, 2955, 2841, 2242, 1737, 1609, 1598, 1475, 1434, 1353, 1246, 1156, 1062, 851, 749 cm^−1^; LRMS-EI^+^ (*m*/*z*) 461.15 ([M + Na]^+^, 100), [M + H]^+^ 439.19 (12.92); HRMS-TOF-ES^+^ (*m*/*z*) [M + H]^+^ calcd for C_24_H_27_N_2_O_6_ 439.1869, found 439.1884.

### Ethyl 3-cyano-4-(2-methoxy-2-oxoethyl)-2-(2,4,6-trimethoxyphenyl)-1,2,3,4-tetrahydroquinoline-3-carboxylate, 5a

Low melting yellow solid; ^1^H NMR (400 MHz, CDCl_3_), 7.13–7.05 (2H, m), 6.87–6.76 (2H, s), 6.11 (2H, s), 5.25 (1H, d, *J* = 7.0 Hz), 4.79 (1H, d, *J* = 7.0 Hz), 4.03–3.09 (3H, m), 3.82–3.76 (12H, m), 2.95 (1H, dd, *J* = 8.4, 12.9 Hz), 2.69 (1H, dd, *J* = 2.8, 16.9 Hz), 0.96 (1H, t, *J* = 7.1 Hz); ^13^C NMR (100 MHz, CDCl_3_) 172.6, 167.3, 161.9, 160.1, 143.3, 127.8, 127.4, 123.1, 120.3, 118.3, 116.8, 104.4, 90.8, 62.4, 55.7, 55.6, 55.4, 52.3, 51.9, 43.0, 37.3, 13.6;. FT-IR (KBr, *ν̃*) 3396, 2940, 2841, 2236, 1734, 1606, 1468, 1333, 1202, 1156, 1122, 809, 749 cm^−1^; LRMS-EI^+^ (*m*/*z*) 469.37 [M + H]^+^ (100), 467.38 (4.39); HRMS-TOF-ES^+^ (*m*/*z*) [M + H]^+^ calcd for C_25_H_29_N_2_O_7_ 469.1975, found 469.1972.

### Ethyl 3-cyano-4-(2-methoxy-2-oxoethyl)-2-(naphthalen-1-yl)-1,2,3,4-tetrahydroquinoline-3-carboxylate, 5b

White powder; mp 138–140 °C; ^1^H NMR (400 MHz, CDCl_3_) 8.27 (1H, d, *J* = 8.0 Hz), 8.06 (1H, d, *J* = 8.0 Hz), 7.92–7.87 (2H, m), 7.62–7.51 (3H, m), 7.15–7.10 (2H, m), 6.86–6.83 (1H, m), 6.68–6.66 (1H, m), 5.68 (1H, b), 4.59–4.56 (1H, m), 4.24 (1H, m), 3.79 (3H, s), 3.70–3.52 (2H, m), 3.02 (1H, dd, *J* = 7.9, 17.1 Hz), 2.75 (1H, dd, *J* = 3.5, 17.1 Hz), 0.53–0.49 (3H, m); ^13^C NMR (100 MHz, CDCl_3_) 172.3, 166.8, 143.0, 133.8, 132.4, 131.1, 129.9, 129.0, 128.2, 127.5, 126.6, 126.0, 125.7, 125.4, 122.5, 120.3, 119.5, 115.2, 63.0, 55.9, 54.8, 52.4, 41.6, 37.4, 13.0; FT-IR (KBr, *ν̃*) 3382, 2912, 2253, 1737, 1602, 1487, 1435, 1372, 1245, 1169, 1040, 856, 748 cm^−1^; LRMS-EI^+^ (*m*/*z*) 451.33 ([M + Na]^+^, 100), [M + H]^+^ 429.58 (12.92); HRMS-TOF-ES^−^ (*m*/*z*) [M + H]^+^ calcd for C_26_H_25_N_2_O_4_ 429.1814, found 429.1814.

### Ethyl 3-cyano-4-(2-methoxy-2-oxoethyl)-2-(naphthalen-2-yl)-1,2,3,4-tetrahydroquinoline-3-carboxylate, 5c

White solid; mp 136–138 °C; ^1^H NMR (400 MHz, CDCl_3_) 8.04 (1H, s), 7.89–7.85 (3H, m), 7.71–7.69 (1H, d *J* = 8.1 Hz), 7.54–7.52 (2H, m), 7.15–7.12 (2H, dd, *J* = 7.4, 7.6 Hz), 7.06 (1H, d, *J* = 8.0 Hz), 6.82 (1H, dd, *J* = 7.7, 7.7 Hz), 6.68 (1H, d, *J* = 8.0 Hz), 4.90 (1H, s), 4.42–4.39 (1H, m), 4.34 (1H, s), 4.0–3.94 (2H, m), 3.79 (3H, s), 3.01 (1H, dd, *J* = 7.9, 17.1 Hz), 2.74 (1H, dd, *J* = 3.7, 17.2 Hz), m), 0.82 (3H, t, *J* = 7.9 Hz); ^13^C NMR (100 MHz, CDCl_3_) 172.2, 142.7, 133.9, 133.7, 1331, 128.8, 128.3, 127.7, 127.7, 127.3, 126.8, 126.6, 125.0, 120.1, 119.4, 115.1, 63.0, 62.4, 55.3, 52.4, 41.2, 37.2, 13.6; FT-IR (KBr, *ν̃*) 3379, 2954, 2242, 1740, 1608, 1483, 1436, 1369, 241, 1160, 1042, 854, 781, 748 cm^−1^; LRMS-EI^+^ (*m*/*z*) 451.33 ([M + Na]^+^, 100), 443.52 (17.75), [M + H]^+^ 429.46 (12.92); HRMS-TOF-ES^+^ (*m*/*z*) [M + H]^+^ calcd for C_26_H_25_N_2_O_4_ 429.1814, found 429.1812.

### Ethyl 3-cyano-4-(2-methoxy-2-oxoethyl)-2-(4-methoxyphenyl)-1,2,3,4-tetrahydroquinoline-3-carboxylate, 5e

White powder; mp 128–130 °C; ^1^H NMR (400 MHz, CDCl_3_) 7.49 (2H, d, *J* = 8.8 Hz), 7.11 (1H, dd, *J* = 7.7, 7.7 Hz), 7.02 (1H, d, *J* = 7.7 Hz), 6.92 (2H, d, *J* = 8.8 Hz), 6.79 (1H, dd, *J* = 7.5, 7.5 Hz), 6.63 (1H, d, *J* = 7.7 Hz), 4.68 (1H, s), 4.33 (1H, m, *J* = 3.7, 7.7 Hz), 4.22 (1H, s), 4.04 (2H, q, *J* = 7.2 Hz), 3.82 (3H, m), 3.78 (3H, m), 2.97 (1H, dd, *J* = 7.9, 17.1 Hz), 2.71 (1H, dd, *J* = 3.7, 16.9 Hz), 1.02 (3H, t, *J* = 7.2 Hz); ^13^C NMR (100 MHz, CDCl_3_) 172.2, 167.0, 160.6, 142.8, 129.1, 128.3, 128.2, 127.3, 112.0, 119.2, 115.0, 114.2, 63.0, 61.7, 55.5, 55.4, 52.4, 41.1, 37.2, 13.8; FT-IR (KBr, *ν̃*) 3381, 2955, 2247, 1734, 1610, 1532, 1485, 1299, 1249, 1176, 1033, 841, 749 cm^−1^; LRMS-EI^+^ (*m*/*z*) 409.49 ([M + H]^+^, 100), 333.43 (13.60), 407.36 (5); HRMS-TOF-ES^+^ (*m*/*z*) [M + H]^+^ calcd for C_23_H_25_N_2_O_5_ 409.1763, found 409.1764.

### Ethyl 6-chloro-3-cyano-4-(2-methoxy-2-oxoethyl)-2-(2-methoxyphenyl)-1,2,3,4-tetrahydroquinoline-3-carboxylate, 9a

White solid; mp 164–166 °C; ^1^H NMR (400 MHz, CDCl_3_) 7.86 (1H, d, *J* = 7.8 Hz), 7.38–7.35 (1H, m), 7.08–7.02 (3H, m), 6.89–6.87 (1H, m), 6.58–6.56 (1H, m), 5.27 (1H, s), 4.40–4.38 (1H, m), 4.15 (1H, s), 4.03–3.96 (2H, m), 3.86 (1H, s), 3.80 (3H, b), 3.78 (3H, b), 2.93 (1H, dd, *J* = 7.9, 17.1 Hz), 2.71 (1H, dd, *J* = 3.4, 17.0 Hz), 0.97 (3H, t, *J* = 7.2 Hz); ^13^C NMR (100 MHz, CDCl_3_) 172.0, 166.2, 157.1, 141.7, 130.5, 128.1, 127.31, 124.4, 123.8, 122.0, 121.3, 116.3, 115.8, 110.5, 62.9, 55.5, 54.2, 53.6, 52.5, 13.5; FT-IR (KBr, *ν̃*) 3389, 2952, 2225, 1722, 1602, 1492, 1465, 1368, 1296, 1258, 1170, 1051, 1023, 862, 824, 755 cm^−1^; LRMS-EI^+^ (*m*/*z*) 465.40 ([M + Na]^+^, 100), 443.60 (43.69), 444.70 (16.67), 445.66 (13.54); HRMS-TOF-ES^+^ (*m*/*z*) [M + H]^+^ calcd for C_23_H_24_ClN_2_O_5_ 443.1374, found 443.1373.

### Ethyl 6-chloro-3-cyano-2-(3,5-dimethoxyphenyl)-4-(2-methoxy-2-oxoethyl)-1,2,3,4-tetrahydroquinoline-3-carboxylate, 9b

White powder; mp 179–181 °C; ^1^H NMR (400 MHz, CDCl_3_), 7.08–7.06 (1H, d, *J* = 8.0 Hz), 6.99 (1H, b), 6.69 (2H, b), 6.59 (1H, d, *J* = 8.8 Hz), 6.48 (1H, m), 4.62 (1H, s), 4.29–4.27 (2H, m), 4.11–4.05 (2H, m), 3.79 (9H, b), 2.94 (1H, dd, *J* = 8.1, 17.2 Hz), 2.71 (1H, dd, *J* = 3.7, 17.2 Hz), 1.04 (3H, t, *J* = 7.2 Hz); ^13^C NMR (100 MHz, CDCl_3_) 171.8, 166.6, 161.1, 141.1, 138.0, 128.3, 127.2, 123.9, 121.5, 116.2, 115.2, 105.7, 101.8, 63.2, 62.3, 55.5, 54.8, 52.5, 41.0, 36.9, 13.7; FT-IR (KBr, *ν̃*) 3379, 2955, 2242, 1734, 1599, 1493, 1470, 1353, 1300, 1245, 1156, 1060, 850, 696 cm^−1^; LRMS-EI^+^ (*m*/*z*) 495.47 ([M + Na]^+^, 100), 473.48 ([M + H]^+^, 13.13), 493.43 (15.24), 494.28 (15.19); HRMS-TOF-ES^+^ (*m*/*z*) [M + H]^+^ calcd for C_24_H_26_ClN_2_O_6_ 473.1479, found 473.1475.

### Ethyl 6-chloro-3-cyano-4-(2-methoxy-2-oxoethyl)-2-(naphthalen-2-yl)-1,2,3,4-tetrahydroquinoline-3-carboxylate, 9c

White powder; mp 172–174 °C; white powder; ^1^H NMR (400 MHz, CDCl_3_) 8.01 (1H, s), 7.91–7.84 (3H, m), 7.69–7.65 (1H, m), 7.55–7.51 (2H, m), 7.12–7.07 (1H, m), 7.03 (1H, s), 6.64–6.60 (1H, m), 4.87 (1H, s), 4.40–4.33 (2H, m), 4.00–3.91 (2H, m), 3.80 (3H, s), 2.97 (1H, dd, *J* = 8.1, 17.2 Hz), 2.74 (1H, dd, *J* = 3.7, 17.2 Hz), 0.81 (3H, t, *J* = 7.2 Hz); ^13^C NMR (100 MHz, CDCl_3_) 171.8, 166.6, 161.1, 141.1, 138.1, 128.3, 127.2, 123.9, 121.5, 116.2, 115.2, 105.7, 101.8, 63.2, 62.3, 55.5, 54.8, 52.5, 41.0, 36.9, 13.7; FT-IR (KBr, *ν̃*) 3376, 2953, 2247, 1737, 1605, 1493, 1369, 1311, 1245, 1169, 1048, 818, 758, 672 cm^−1^; LRMS-EI^+^ (*m*/*z*) 485.43 ([M + Na]^+^, 100), 463.54 (30.42). HR-MS-EI^+^ (*m*/*z*) [M]^+^ calcd for C_26_H_23_ClN_2_O_4_ 462.1346, found 462.1347.

### Ethyl 6-chloro-3-cyano-4-(2-methoxy-2-oxoethyl)-2-(naphthalen-1-yl)-1,2,3,4-tetrahydroquinoline-3-carboxylate, 9d

White solid; mp 230–232 °C; ^1^H NMR (400 MHz, CDCl_3_) 8.23 (1H, d, *J* = 7.3 Hz), 8.03 (1H, d, *J* = 8.1 Hz), 7.93–7.88 (2H, m), 7.62–7.49 (3H, m), 7.11–7.08 (2H, m), 6.62–6.59 (1H, m), 5.65 (1H, m), 4.54–4.51 (1H, m), 4.26 (1H, b), 3.80 (3H, s), 3.68–3.52 (2H, m), 2.99 (1H, dd, *J* = 7.9, 17.1 Hz), 2.74 (1H, dd, *J* = 3.2, 17.0 Hz), 1.29–1.24 (1H, m), 0.51 (3H, t, *J* = 7.0 Hz); ^13^C NMR (100 MHz, CDCl_3_) 171.9, 166.5, 141.6, 133.8, 132.0, 131.0, 130.1, 129.0, 128.3, 127.4, 126.7, 126.1, 125.7, 125.3, 124.1, 122.3, 121.8, 116.3, 115.6, 63.1, 55.8, 54.4, 52.5, 41.5, 37.2, 13.0; FT-IR (KBr, *ν̃*) 3372, 2951, 2236, 1728, 1706, 1610, 1508, 1435, 1294, 1234, 1190, 1116, 1068, 784 cm^−1^; LRMS-EI^+^ (*m*/*z*) 485.22 ([M + Na]^+^, 100), 463.32 ([M + H]^+^, 15.47), 443.39 (18.56); HRMS-TOF-ES^+^ (*m*/*z*) [M + H]^+^ calcd for C_26_H_24_ClN_2_O_5_ 463.1425, found 463.1427.

### Ethyl 6-chloro-3-cyano-4-(2-methoxy-2-oxoethyl)-2-(pyridin-2-yl)-1,2,3,4-tetrahydroquinoline-3-carboxylate, 9e

White solid; mp 136–138 °C; ^1^H NMR (400 MHz, CDCl_3_) 8.68 (1H, d, *J* = 4.7 Hz), 7.78–7.73 (1H, m), 7.44 (1H, d, *J* = 7.7 Hz), 7.35 (1H, dd, *J* = 5.0, 6.96 Hz), 7.11 (1H, dd, *J* = 1.9, 8.6 Hz), 7.03 (1H, s), 6.74 (1H, d, *J* = 8.8 Hz), 5.00 (1H, d, *J* = 4.2 Hz), 4.87 (1H, d, *J* = 4.2 Hz), 4.29 (1H, dd, *J* = 2.8, 7.9 Hz), 4.19–4.12 (2H, m), 3.81 (3H, s), 2.95 (1H, dd, *J* = 8.1, 17.2 Hz), 2.74 (1H, dd, *J* = 3.3, 17.2 Hz), 1.12 (3H, dd, *J* = 7.2, 7.2 Hz); ^13^C NMR (100 MHz, CDCl_3_) 171.9, 161.2, 154.5, 149.6, 140.5, 137.1, 128.4, 127.0, 124.6, 124.0, 122.7, 122.4, 118.1, 114.5, 63.2, 62.2, 52.8, 52.5, 41.1, 36.6, 13.8; FT-IR (KBr, *ν̃*) 3368, 2954, 2242, 1737, 1590, 1493, 1471, 1437, 1243, 1170.3, 1049, 816, 753 cm^−1^; LRMS-EI^+^ (*m*/*z*) 436.20 ([M + Na]^+^, 100), 414.24 ([M + H]^+^ 93.14); HRMS-TOF-ES^+^ (*m*/*z*) [M + H]^+^ calcd for C_21_H_25_ClN_3_O_4_ 414.1221, found 414.1215.

### Ethyl 6-chloro-3-cyano-4-(2-methoxy-2-oxoethyl)-2-(thiophen-3-yl)-1,2,3,4-tetrahydroquinoline-3-carboxylate (9f)

White solid; mp 116–118 °C; ^1^H NMR (400 MHz, CDCl_3_) 7.86 (1H, d, *J* = 7.3 Hz), 7.36 (1H, t, *J* = 7.9 Hz), 7.09–7.01 (3H, m), 6.90–6.87 (1H, m), 6.58–6.54 (1H, m), 5.26 (1H, s), 4.38 (1H, m), 4.15 (1H, s), 4.00 (2H, q, *J* = 7.1 Hz), 3.79–3.80 (6H, b), 2.97–2.88 (1H, dd, *J* = 7.7, 16.9 Hz), 2.71 (1H, dd, *J* = 3.1, 17.1 Hz), 0.96 (3H, t, *J* = 7.2 Hz); ^13^C NMR (100 MHz, CDCl_3_) 172.0, 166.2, 157.1, 141.7, 130.6, 128.1, 127.3, 124.5, 123.8, 122.0, 1213, 116.3, 115.8, 110.5, 62.9, 55.5, 55.2, 53.6, 52.5, 41.0, 37.2, 13.5; FT-IR (KBr, *ν̃*) 3380, 2954, 2247, 1739, 1606, 1492, 1244, 1170, 1049, 814, 751, 606 cm^−1^; LRMS-EI^+^ (*m*/*z*) 441.33 ([M + Na]^+^, 100), 419.28 ([M + H]^+^ 7.96); HRMS-TOF-ES^+^ (*m*/*z*) [M + H]^+^ calcd for C_20_H_20_ClN_2_O_4_S 419.0832, found 419.0829.

### Ethyl 6-chloro-3-cyano-4-(2-methoxy-2-oxoethyl)-2-(thiophen-2-yl)-1,2,3,4-tetrahydroquinoline-3-carboxylate, 9g

White solid; ^1^H NMR (400 MHz, CDCl_3_) 7.36 (1H, d, *J* = 5.0 Hz), 7.26 (1H, m), 7.08–7.01 (3H, m), 6.59 (1H, d, *J* = 8.4 Hz), 5.03 (1H, s), 4.38 (1H, s), 4.27 (1H, dd, *J* = 3.9, 7.5 Hz), 4.16–4.05 (2H, m), 3.78 (3H, m), 2.94 (1H, dd, *J* = 7.7, 17.2 Hz), 2.74 (1H, dd, *J* = 3.9, 17.3 Hz), 1.08 (3H, t, *J* = 7.2 Hz); ^13^C NMR (100 MHz, CDCl_3_) 171.8, 166.6, 140.8, 138.0, 128.4, 127.4, 127.3, 127.0, 126.7, 124.3, 121.7, 116.4, 115.0, 63.4, 58.2, 55.8, 52.5, 40.8, 37.0, 13.7; FT-IR (KBr, *ν̃*) 2918, 2857, 2335, 1739, 1657, 1599, 1575, 1487, 1361, 1380, 1306, 1246, 1161, 1045, 815, 705 cm^−1^; LRMS-EI^+^ (*m*/*z*) 441.07 ([M + Na]^+^, 100), 419.03 ([M + H]^+^, 24.40). HRMS-TOF-ES^+^ (*m*/*z*) [M + H]^+^ calcd for C_20_H_20_ClN_2_O_4_S 419.0832, found 419.0833.

### 3-Ethyl 6-methyl 3-cyano-4-(2-methoxy-2-oxoethyl)-2-(naphthalen-2-yl)-1,2,3,4-tetrahydroquinoline-3,6-dicarboxylate, 9h

White powder; mp 196–198 °C; ^1^H NMR (400 MHz, CDCl_3_) 8.02 (1H, s), 7.99–7.80 (6H, m), 7.68–7.66 (1H, d, *J* = 8.5 Hz), 7.62–7.45 (2H, m), 6.69 (1H, d, *J* = 8.4 Hz), 5.00 (1H, s), 4.79 (1H, s), 4.41–4.34 (1H, m), 4.05–3.94 (2H, m), 3.87 (3H, s), 3.83 (3H, s), 3.07 (1H, dd, *J* = 8.1, 16.9 Hz), 2.73 (1H, dd, *J* = 3.8, 16.9 Hz), 0.85 (3H, t, *J* = 7.2 Hz); ^13^C NMR (100 MHz, CDCl_3_) 171.9, 166.8, 166.6, 146.5, 134.0, 133.0, 132.9, 130.2, 129.6, 129.0, 128.3, 127.7, 127.7, 127.1, 126.8, 124.70, 119.0, 115.0, 114.2, 63.3, 62.1, 54.5, 52.5, 51.8, 41.3, 36.5, 13.6; FT-IR (KBr, *ν̃*) 3376, 2952, 2236, 1737, 1713, 1611, 1515, 1436, 1370, 1298, 1241, 11 112, 1049, 855, 767 cm^−1^; LRMS-EI^+^ (*m*/*z*) 509.17 ([M + Na]^+^, 100), 487.26 ([M + H]^+^, 12.21), 443.31 (16.46); HRMS-TOF-ES^+^ (*m*/*z*) [M + H]^+^ calcd for C_28_H_27_N_2_O_6_ 487.1869, found 487.1865.

### 3-Ethyl 6-methyl 3-cyano-4-(2-methoxy-2-oxoethyl)-2-(naphthalen-1-yl)-1,2,3,4-tetrahydroquinoline-3,6-dicarboxylate, 9i

White solid; mp 230–232 °C; ^1^H NMR (400 MHz, CDCl_3_) 8.22 (1H, d, *J* = 7.3 Hz), 8.03 (1H, d, *J* = 8.2 Hz), 7.94–7.80 (4H, m), 7.62–7.52 (3H, m), 6.64 (1H, d, *J* = 8.3 Hz), 5.76 (1H, s), 4.70 (1H, s), 4.55 (1H, dd, *J* = 3.2, 7.7 Hz), 3.87 (3H, s), 3.82 (3H, s), 3.68–3.52 (2H, m), 3.06 (1H, dd, *J* = 8.1, 16.8 Hz), 2.73 (1H, dd, *J* = 3.4, 16.8 Hz), 0.52 (3H, t, *J* = 7.2 Hz); ^13^C NMR (100 MHz, CDCl_3_) 171.98, 166.8, 166.4, 146.8, 133.8, 131.7, 130.9, 130.3, 130.2, 129.8, 129.1, 126.2, 125.7, 125.4, 122.3, 120.5, 119.1, 115.4, 114.2, 63.2, 55.5, 53.9, 52.5, 51.9, 41.6, 36.8, 13.0; FT-IR (KBr, *ν̃*) 3372, 2951, 2236, 1728, 1707, 1610, 1508, 1435, 1334, 1293.9, 1234, 1190, 1116, 1068, 784 cm^−1^; LRMS-EI^+^ (*m*/*z*) 509.20 ([M + Na]^+^, 100), 487.25 ([M + H]^+^, 10.25), 443.23 (16.84); HRMS-TOF-ES^+^ (*m*/*z*) [M + H]^+^ calcd for C_28_H_27_N_2_O_6_ 488.1869, found 487.1876.

### 3-Ethyl 6-methyl 3-cyano-4-(2-methoxy-2-oxoethyl)-2-(2-methoxyphenyl)-1,2,3,4-tetrahydroquinoline-3,6-dicarboxylate, 9j

White solid; mp 212–215 °C; ^1^H NMR (400 MHz, CDCl_3_) 7.86 (1H, d, *J* = 8.8 Hz), 7.78–7.76 (2H, m), 7.37 (1H, dd, *J* = 7.7, 7.7 Hz), 7.06 (1H, dd, *J* = 7.5, 7.5 Hz), 6.89 (1H, d, *J* = 8.4 Hz), 6.60 (1H, d, *J* = 8.8 Hz), 5.39 (1H, s), 4.55 (1H, s), 4.38 (1H, dd, *J* = 2.8, 7.9 Hz), 4.11–3.92 (2H, m), 3.85 (3H, s), 3.82 (3H, s), 3.79 (3H, s), 3.01 (1H, dd, *J* = 8.1, 16.7 Hz), 2.70 (1H, dd, *J* = 3.3, 16.7 Hz), 0.99 (3H, t, *J* = 7.2 Hz); ^13^C NMR (100 MHz, CDCl_3_) 172.0, 166.9, 166.1, 157.1, 147.0, 130.7, 130.0, 129.6, 128.0, 124.1, 121.3, 120.2, 119.2, 115.6, 114.5, 110.6, 63.0, 55.5, 53.8, 53.2, 52.4, 51.7, 41.1, 36.8, 13.5; FT-IR (KBr, *ν̃*) 3374, 2945, 2396, 2242, 1739, 1709, 1611, 1436, 1298, 1241, 1113, 767 cm^−1^; LRMS-EI^+^ (*m*/*z*): 489.16 ([M + Na]^+^, 100), 467.23 ([M + H]^+^, 8.41); HRMS-TOF-ES^+^ (*m*/*z*) [M + H]^+^ calcd for C_25_H_27_N_2_O_7_ 467.1818, found 467.1816.

### Ethyl 3-cyano-2-(3,5-dimethoxyphenyl)-4-(2-methoxy-2-oxoethyl)-1,2,3,4-tetrahydroquinoline-3-carboxylate, 9k

White powder; mp 110–112 °C; ^1^H NMR (400 MHz, CDCl_3_) 7.81–7.78 (2H, m), 6.69 (2H, s), 6.64 (1H, d, *J* = 8.4 Hz), 6.48 (1H, s), 4.73 (1H, s), 4.31 (1H, dd, *J* = 3.0, 7.6 Hz), 4.18–4.04 (2H, m), 3.86–3.79 (12H, m), 3.05 (1H, dd, *J* = 8.0, 16.9 Hz), 2.72 (1H, dd, *J* = 3.6, 16.9 Hz), 1.12–1.06 (3H, t, *J* = 7.12 Hz); ^13^C NMR (100 MHz, CDCl_3_) 171.8, 166.8, 66.5, 161.2, 146.4, 137.7, 130.2, 129.6, 120.5, 118.9, 115.0, 114.2, 105.7, 101.9, 63.3, 62.0, 55.5, 54.3, 52.5, 51.8, 41.2, 36.5, 13.7; FT-IR (KBr, *ν̃*) 2954, 2841, 2242, 1737, 1709, 1610, 1514, 1466, 1436, 1299, 1240, 1205, 1113, 992, 850, 768 cm^−1^; LRMS-EI^+^ (*m*/*z*) 519.55 ([M + Na]^+^, 100), 577.00 (2.36), 581.12 (5.13). HRMS-TOF-ES^+^ (*m*/*z*) [M + H]^+^ calcd for C_26_H_29_N_2_O_8_ 497.1924, found 497.1921.

### Ethyl 3-cyano-2-(naphthalen-2-yl)-4-(2-oxo-2-phenylethyl)-1,2,3,4-tetrahydroquinoline-3-carboxylate, 9l

White solid; mp 196–198 °C; ^1^H NMR (400 MHz, CDCl_3_) 8.09–8.07 (3H, m), 7.90–7.85 (3H, m), 7.74–7.72 (1H, d, *J* = 8.2 Hz), 7.63 (1H, t, *J* = 7.3 Hz), 7.54–7.50 (4H, m), 7.11 (1H, t, *J* = 7.5 Hz), 6.87–6.85 (1H, d, *J* = 7.8 Hz), 6.72 (2H, dd, *J* = 8.1, 16.5 Hz), 4.98 (1H, s), 4.82–4.77 (1H, m), 4.39 (1H, s), 3.92 (2H, q, *J* = 7.2 Hz), 3.80 (1H, dd, *J* = 7.7, 18.3 Hz), 3.30 (1H, dd, *J* = 2.9, 18.3 Hz), 0.79 (3H, t, *J* = 7.2 Hz); ^13^C NMR (100 MHz, CDCl_3_) 196.9, 166.9, 142.8, 136.1, 133.9, 133.8, 133.1, 128.9, 128.8, 128.4, 128.3, 128.1, 127.7, 126.8, 126.6, 125.1, 120.6, 119.3, 116.1, 115.1, 63.0, 62.3, 55.7, 42.0, 39.9, 13.6; FT-IR (KBr, *ν̃*): 3377, 3057, 2247, 1739, 1686, 1607, 1485, 1366, 1318, 12 445, 1052, 820, 751, 639 cm^−1^; LRMS-EI^+^ (*m*/*z*) 497.19 ([M + Na]^+^, 100), 475.21 ([M + H]^+^ 21.10); HRMS-TOF-ES^+^ (*m*/*z*) [M + H]^+^ calcd for C_31_H_27_N_2_O_3_ 475.2022, found 475.2021.

### Ethyl 3-cyano-2-(2-methoxyphenyl)-4-(2-oxo-2-phenylethyl)-1,2,3,4-tetrahydroquinoline-3-carboxylate, 9m

White solid; mp 86–88 °C; ^1^H NMR (400 MHz, CDCl_3_) 8.07 (2H, m, *J* = 7.2 Hz), 7.93 (1H, d, *J* = 7.6 Hz), 7.62 (1H, t, *J* = 7.2 Hz), 7.52 (2H, m), 7.36 (1H, t, *J* = 7.9 Hz), 7.09–7.04 (2H, m), 6.86 (2H, dd, *J* = 8.1, 26.8 Hz), 6.69 (1H, t, *J* = 7.3 Hz), 6.63 (1H, d, *J* = 7.9 Hz), 5.39 (1H, s), 4.83 (1H, d, *J* = 7.7 Hz), 4.15 (1H, s), 4.02–3.88 (2H, m), 3.81–3.73 (4H, m), 3.25 (1H, dd, *J* = 2.2, 18.3 Hz), 0.93 (3H, t, *J* = 7.2 Hz); ^13^C NMR (100 MHz, CDCl_3_) 196.9, 189.4, 166.5, 157.2, 143.2, 136.3, 133.6, 130.3, 128.8, 128.7, 128.4, 128.2, 127.7, 125.1, 121.3, 121.0, 119.2, 116.8, 115.2, 110.6, 62.7, 55.5, 54.4, 54.2, 42.2, 39.8, 13.5; FT-IR (KBr, *ν̃*) 3382, 2923, 2346, 1740, 1687, 1602, 1493, 1289, 1248, 1052 753 cm^−1^; LRMS-EI^+^ (*m*/*z*) 477.17 ([M + Na]^+^, 100), 455.30 ([M + H]^+^, 15.55); HRMS-TOF-ES^+^ (*m*/*z*) [M + H]^+^ calcd for C_28_H_27_N_2_O_4_ 455.1971, found 455.1972.

### Ethyl 3-cyano-2-(4-nitrophenyl)-4-(2-oxo-2-phenylethyl)-1,2,3,4-tetrahydroquinoline-3-carboxylate (major), 9n

Yellow crystal; mp 156–158 °C; ^1^H NMR (400 MHz, CDCl_3_) 8.31–8.29 (1H, d, *J* = 7.2 Hz), 8.12–8.09 (3H, m), 7.93–7.88 (2H, dd, *J* = 8.3, 11.6 Hz), 7.65–7.49 (6H, m), 7.13–7.09 (2H, m), 6.89 (1H, d, *J* = 7.6 Hz), 6.76–6.67 (2H, m), 5.78 (1H, b), 5.00 (1H, b), 4.27 (1H, b), 3.83 (1H, dd, *J* = 7.9, 18.3 Hz), 3.66–3.53 (2H, m), 3.30–3.26 (1H, m), 0.49 (3H, t, *J* = 7.2 Hz); ^13^C NMR (100 MHz, CDCl_3_) 196.9, 166.8, 143.1, 136.1, 133.8, 132.6, 131.1, 129.9, 128.9, 128.0, 127.9, 126.7, 126.0, 125.7, 125.3, 122.6, 120.9, 119.5, 116.6, 115.2, 63.0, 55.9, 55.1, 42.2, 40.3, 13.0; FT-IR (KBr, *ν̃*) 3382, 3056, 2242, 1737, 1686, 1597, 1481, 1365, 1240, 1052, 781, 752, 690 cm^−1^; LRMS-EI^+^ (*m*/*z*) 497.32 ([M + Na]^+^, 100), 475.38 ([M + H]^+^, 4.05); HRMS-TOF-ES^+^ (*m*/*z*) [M + H]^+^ calcd for C_31_H_27_N_2_O_3_ 475.2022, found 475.2020.

### Ethyl 3-cyano-4-(2-oxo-2-phenylethyl)-2-(thiophen-2-yl)-1,2,3,4-tetrahydroquinoline-3-carboxylate, 9o

White powder; mp 170–172 °C; ^1^H NMR (400 MHz, CDCl_3_) 8.08–8.06 (2H, d, *J* = 7.5 Hz), 7.63 (1H, t, *J* = 7.3 Hz), 7.52 (2H, t, *J* = 7.5 Hz), 7.36–7.30 (2H, m), 7.11–7.03 (2H, m), 6.84–6.82 (1H, d, *J* = 7.7 Hz), 6.74–6.68 (2H, m), 5.15 (1H, s), 4.72 (1H, dd, *J* = 2.6, 7.0 Hz), 4.39 (1H, s), 4.14–4.02 (2H, m), 3.77 (1H, dd, *J* = 7.5, 18.5 Hz), 3.34 (1H, dd, *J* = 3.1, 18.5 Hz), 1.05 (3H, t, *J* = 7.2 Hz); ^13^C NMR (100 MHz, CDCl_3_) 196.8, 166.8, 142.3, 138.8, 136.1, 133.7, 128.9, 128.4, 128.1, 127.6, 127.3, 126.9, 126.5, 120.7, 119.7, 115.9, 115.3, 63.1, 58.1, 56.6, 42.1, 39.5, 13.7; FT-IR (KBr, *ν̃*) 3369, 2929, 2242, 1737, 1685, 1607, 1482, 1365, 1242, 1052, 971, 855, 751, 690 cm^−1^; LRMS-EI^+^ (*m*/*z*) 453.20 ([M + Na]^+^, 100), 451.36 (21.10), 431.33 ([M + H]^+^, 1.34); HRMS-TOF-ES^+^ (*m*/*z*) [M + H]^+^ calcd for C_25_H_23_N_2_O_3_S 431.1429, found 431.1414.

### Ethyl 3-cyano-2-(3,5-dimethoxyphenyl)-4-(2-oxo-2-phenylethyl)-1,2,3,4-tetrahydroquinoline-3-carboxylate, 9p

White solid; mp 130–132 °C; ^1^H NMR (400 MHz, CDCl_3_) 8.07 (2H, d, *J* = 7.3 Hz), 7.63 (1H, t, *J* = 7.3 Hz), 7.52 (2H, t, *J* = 7.7 Hz), 7.09 (1H, t, *J* = 7.5 Hz), 6.82–6.65 (5H, m), 6.48 (1H, t, *J* = 2.0 Hz), 4.74 (1H, b), 4.29 (1H, s), 4.10–3.98 (2H, m), 3.80 (6H, m), 3.76 (1H, dd, *J* = 3.3, 10.5 Hz), 3.25 (1H, dd, *J* = 2.8, 18.5 Hz), 1.01 (3H, t, *J* = 7.2 Hz); ^13^C NMR (100 MHz, CDCl_3_) 196.8, 166.9, 161.0, 142.6, 138.7, 136.1, 133.8, 128.9, 128.0, 127.7, 120.5, 119.3, 116.1, 115.0, 105.7, 101.7, 63.0, 62.2, 55.5, 41.9, 39.8, 13.8; FT-IR (KBr, *ν̃*) 3374, 2923, 1597, 2835, 2247, 1737, 1685, 1597, 1473, 1347, 1202, 1243, 1158, 1059, 987, 933, 749, 694, 634 535 cm^−1^; LRMS-EI^+^ (*m*/*z*) 507.48 ([M + Na]^+^, 100), 485.52, ([M + H]^+^, 4.80); HRMS-TOF-ES^+^ (*m*/*z*) [M + H]^+^ calcd for C_29_H_29_N_2_O_5_ 485.2076, found 485.2075.

### Ethyl 3-cyano-2-(4-nitrophenyl)-4-(2-oxo-2-phenylethyl)-1,2,3,4-tetrahydroquinoline-3-carboxylate, 9q

Yellow crystal; mp 198–200 °C; ^1^H NMR (400 MHz, CDCl_3_) 8.27 (2H, d, *J* = 8.6 Hz), 8.06 (2H, d, *J* = 7.3 Hz), 7.84 (2H, d, *J* = 8.6 Hz), 7.63 (1H, t, *J* = 7.2 Hz), 7.54–7.50 (2H, m), 7.11 (1H, t, *J* = 7.3 Hz), 6.87–6.85 (1H, m), 6.78–6.68 (2H, m), 4.96 (1H, s), 4.76–4.72 (1H, m), 4.26 (1H, s), 4.01 (2H, q, *J* = 7.1 Hz), 3.77 (1H, dd, *J* = 7.3, 18.7 Hz), 3.38–3.30 (1H, dd, *J* = 3.2, 18.6 Hz), 0.97 (3H, t, *J* = 7.2 Hz); ^13^C NMR (100 MHz, CDCl_3_) 196.6, 166.5, 148.8, 143.5, 142.2, 135.9, 133.9, 129.3, 128.9, 128.4, 128.3, 127.7, 124.0, 120.6, 120.0, 115.4, 115.4, 63.4, 61.2, 55.5, 42.0, 39.7, 13.8; FT-IR (KBr, *ν̃*) 3376, 2929, 2242, 1739, 1686, 1608, 1524, 1488, 1347, 1246, 1109, 659, 750, 690 cm^−1^; LRMS-EI^+^ (*m*/*z*) 492.50 ([M + Na]^+^, 100), 470.56 ([M + H]^+^, 15.16), 443.7 (11.79); HRMS-TOF-ES^+^ (*m*/*z*) [M + H]^+^ calcd for C_27_H_24_N_3_O_5_ 470.1716, found 470.1719.

### Ethyl 3-cyano-4-(2-oxo-2-phenylethyl)-2-(2,4,6-trimethoxyphenyl)-1,2,3,4-tetrahydroquinoline-3-carboxylate, 9r

White powder; mp 188–190 °C; ^1^H NMR (400 MHz, CDCl_3_) 8.13–8.11 (2H, m), 7.66–7.52 (3H, m), 7.13–7.09 (1H, m), 6.88–6.75 (3H, m), 6.15 (2H, b), 5.36 (1H, d, *J* = 7.7 Hz), 4.91–4.70 (2H, m), 4.06–3.78 (12H, m), 3.25 (1H, dd, *J* = 1.8, 16.6 Hz), 0.94 (3H, t, *J* = 7.2 Hz); ^13^C NMR (100 MHz, CDCl_3_) 197.3; 167.4, 161.8, 160.1, 143.4, 136.4, 133.6, 128.8, 128.4, 127.7, 127.6, 123.5, 120.2, 118.2, 117.5, 104.5, 90.7, 62.3, 55.7, 55.4, 52.2, 42.1, 41.8, 13.6; FT-IR (KBr, *ν̃*) 3392, 2938, 2236, 1737, 1686, 1602, 1583, 1468, 1231, 1155, 1137, 1106, 972, 816, 748, 690 cm^−1^; LRMS-EI^+^ (*m*/*z*) 514.55 ([M + H]^+^, 100), 537.15 ([M + Na]^+^, 55.28), 538.1 (29.63); HRMS-TOF-ES^+^ (*m*/*z*) [M + H]^+^ calcd for C_30_H_31_N_2_O_6_ 515.2182, found 515.2181.

## Conflicts of interest

There are no conflicts to declare.

## Supplementary Material

RA-010-D0RA01264E-s001

RA-010-D0RA01264E-s002

RA-010-D0RA01264E-s003

## References

[cit1] Nicolaou K. C., Edmonds D. J., Bulger P. G. (2006). Angew. Chem., Int. Ed..

[cit2] Witherup K. M., Ransom R. W., Graham A. C., Bernard A. M., Salvatore M. J., Lumma W. C., Anderson P. S., Pitzenberger S. M., Varga S. L. (1995). J. Am. Chem. Soc..

[cit3] Rakotoson J. H., Fabre N., Jacquemond-Collet I., Hannedouche S., Fourasté I., Moulis C. (1998). Planta Med..

[cit4] Jacquemond-Collet I., Benoit-Vical F., Mustofa M. K. A., Valentin A., Stanislas E., Mallié M., Fourasté I. (2002). Planta Med..

[cit5] Satyanarayana G., Pflästerer D., Helmchen G. (2011). Eur. J. Org. Chem..

[cit6] Maillard M. C., Perlman M. E., Amitay O., Baxter D., Berlove D., Connaughton S., Fischer J. B., Guo J. Q., Hu L.-Y., McBurney R. N., Nagy P. I., Subbarao K., Yost E. A., Zhang L., Durant G. J. (1998). J. Med. Chem..

[cit7] Gore V. K., Ma V. V., Yin R., Ligutti J., Immke D., Doherty E. M., Norman M. H. (2010). Bioorg. Med. Chem. Lett..

[cit8] Grønlien J. H., Haakerud M., Wenn H., Thorin-Hagene K., Briggs C. A., Gopalakrishnan M., Malysz J. (2007). Mol. Pharmacol..

[cit9] Faghih R., Gopalakrishnan M., Briggs C. A. (2008). J. Med. Chem..

[cit10] Bologa C. G., Revankar C. M., Young S. M., Edwards B. S., Arterburn J. B., Kiselyov A. S., Parker M. A., Tkachenko S. E., Savchuck N. P., Sklar L. A., Oprea T. I., Prossnitz E. R. (2006). Nat. Chem. Biol..

[cit11] Su D. S., Lim J. J., Tinney E., Wan B. L., Young M. B., Anderson K. D., Rudd D., Munshi V., Bahnck C., Felock P. J., Lu M., Lai M. T., Touch S., Moyer G., DiStefano D. J., Flynn J. A., Liang Y., Sanchez R., Prasad S., Yan Y., Perlow-Poehnelt R., Torrent M., Miller M., Vacca J. P., Williams T. M., Anthony N. J. (2009). Bioorg. Med. Chem. Lett..

[cit12] Asberom T., Bara T. A., Clader J. W., Greenlee W. J., Guzik H. S., Josien H. B., Li W., Parker E. M., Pissarnitski D. A., Song L., Zhang L., Zhao Z. (2007). Bioorg. Med. Chem. Lett..

[cit13] Nallan L., Bauer K. D., Bendale P., Rivas K., Yokoyama K., Hornéy C. P., Pendyala P. R., Floyd D., Lombardo L. J., Williams D. K., Hamilton A., Sebti S., Windsor W. T., Weber P. C., Buckner F. S., Chakrabarti D., Gelb M. H., Van Voorhis W. C. (2005). J. Med. Chem..

[cit14] Wang X.-F., Wang S.-B., Ohkoshi E., Wang L.-T., Hamel E., Qian K., Morris-Natschke S. L., Lee K.-H., Xie L. (2013). Eur. J. Med. Chem..

[cit15] Escribano A., Mateo A. I., Martin de la Nava E. M., Mayhugh D. R., Cockerham S. L., Beyer T. P., Schmidt R. J., Cao G., Zhang Y., Jones T. M., Borel A. G., Sweetana S. A., Cannady E. A., Mantlo N. B. (2012). Bioorg. Med. Chem. Lett..

[cit16] Leeson P. D., Carling R. W., Moore K. W., Moseley A. M., Smith J. D., Stevenson G., Chan T., Baker R., Foster A. C. (1992). J. Med. Chem..

[cit17] Snider B. B., Ahn Y., O'Hare S. M. (2001). Org. Lett..

[cit18] Sridharan V., Suryavanshi P. A., Menéndez J. C. (2011). Chem. Rev..

[cit19] Nammalwar B., Bunce R. (2014). Molecules.

[cit20] Katritzky A. R., Rachwal S., Rachwal B. (1996). Tetrahedron.

[cit21] Muthukrishnan I., Sridharan V., Menéndez J. C. (2019). Chem. Rev..

[cit22] Hayashi H., Nakatani T., Inoue Y., Nakayama M., Nozaki H. (1997). Biosci., Biotechnol., Biochem..

[cit23] Bunce R. A., Herron D. M., Johnson L. B., Kotturi S. V. (2001). J. Org. Chem..

[cit24] Povarov L. S. (1967). Russ. Chem. Rev..

[cit25] Sridharan V., Avendaño C., Menéndez J. C. (2008). Synthesis.

[cit26] Rui T. H., Fen N. H., Jun C., Jian W. (2012). Chem.–Eur. J..

[cit27] Patra A., Mukherjee S., Das T. K., Jain S., Gonnade R. G., Biju A. T. (2017). Angew. Chem., Int. Ed..

[cit28] Sharif S. A. I., Calder E. D. D., Delolo F. G., Sutherland A. (2016). J. Org. Chem..

[cit29] Ying A.-g., Liu L., Wu G.-f., Chen X.-z., Ye W.-d., Chen J.-h., Zhang K.-y. (2008). Chem. Res. Chin. Univ..

[cit30] Yeom C.-E., Kim M. J., Kim B. M. (2007). Tetrahedron.

