# Supplementary material for: Synthesis of highly substituted tetrahydroquinolines using ethyl cyanoacetate via aza-Michael–Michael addition
Source: RSC Adv. 2020 Apr 3;10(23):13591–600. doi: 10.1039/d0ra01264e (PMC9051536; doi:10.1039/d0ra01264e)
Supplement: RA-010-D0RA01264E-s001 [file RA-010-D0RA01264E-s001.pdf]

Electronic Supplementary Material (ESI) for RSC Advances.
This journal is © The Royal Society of Chemistry 2020

Synthesis of highly substituted tetrahydroquinolines using ethyl cyanoacetate via aza-Michael-Michael addition

A three-component cascade reaction of 2-alkenyl aniline, aldehydes, and ethyl cyanoacetate mediated by DBU to synthesize highly substituted 1,2,3,4-tetrahydroquinolines. The reaction proceeded through the Knoevenagel condensation of ethyl cyanoacetate with aldehydes followed by the aza–Michael–Michael addition with 2-alkenyl anilines to prepare the tetrahydroquinoline scaffolds.

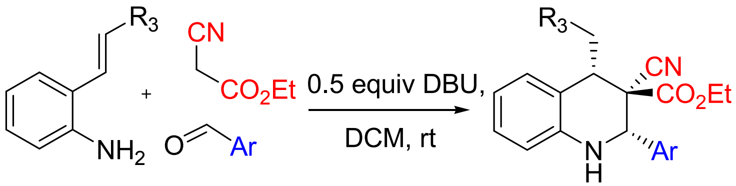

One pot -three component
No metals or strong oxidants
Simple reaction conditions
Verstaile to different
Michael Acceptors

dr up to 93:7
yields upto 96%,
27 examples
