# Supplementary material for: Synthesis of highly substituted tetrahydroquinolines using ethyl cyanoacetate via aza-Michael–Michael addition
Source: RSC Adv. 2020 Apr 3;10(23):13591–600. doi: 10.1039/d0ra01264e (PMC9051536; doi:10.1039/d0ra01264e)

Electronic Supplementary Material (ESI) for RSC Advances.
This journal is © The Royal Society of Chemistry 2020

Cascade synthesis of highly substituted tetrahydroquinolines using ethyl cyanoacetate via aza-Michael-Michael addition

Arunan Palanimuthu,[b] Chinpiao Chen*[a, b] and Gene-Hsian Lee[c]

[a] *Department of Nursing, Tzu Chi University of Science and Technology, Hualien 970, Taiwan, Fax: 886 3 856 1097; Tel: 886 3 857 2158 ext.2624; E-mail: chinpiao@ems.tcust.edu.tw*

[b] *Department of Chemistry, National Dong Hwa University, Soufeng, Hualien 974, Taiwan.*

[c] *Instrumentation Center, College of Science, National Taiwan University, Taipei 106, Taiwan.*

Table of Contents

General procedure for synthesis of substituted *ortho*-amino cinnamates…………….. S2

General Procedure for preparing benzylidene amino phenyl acrylate…....................S4

General procedure for synthesis Knoevenagel intermediate……………………… S4

Crystallographic data of **3a**……………………………………….........................S5

Crystallographic data of **4a**……………………………………….........................S18

^{1}H and ^{13}C NMR Spectra …………………..…………………………….……….S40

General procedure for synthesis of substituted *ortho*-amino cinnamates

Methyl (*E*)-4-amino-3-(3-methoxy-3-oxoprop-1-en-1-yl)benzoate (i).

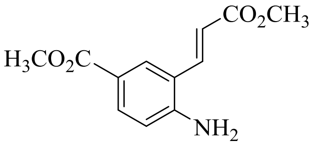

To the stirred solution of methyl 4-amino-3-iodobenzoate (3.61 mmol), palladium acetate (0.036 mmol), triphenylphosphine (0.072 mmol), potassium carbonate (7.22 mmol), and tetrabutylammonium bromide (0.072 mmol) in *N,N′*-dimethylformamide (10 mL) added methyl acrylate (7.22 mmol). The reaction mixture was stirred at 80 °C for 2 h in sealed tube. The mixture was cooled to room temperature, diluted with water (100 mL), and extracted with ethyl acetate (3 × 30 mL). The organic layer was washed with brine (10 mL), dried using $MgSO_4$, filtered, and concentrated in vacuo. The residue was purified by column chromatography (diethyl ether/petroleum ether, 1:4) to give (*E*)-methyl 4-amino-3-(3-methoxy-3-oxoprop-1-en-1-yl) benzoate (2.51 mmol) as light brown solid. Mp 174–176 °C; ^{1}H NMR (400 MHz, DMSO) δ 7.98 (1H, m), 7.88–7.84 (1H, d, J = 15.6 Hz)), 7.64 (1H, dd, J = 1.7, 8.6 Hz), 6.74–6.70 (1H, d, J = 15.6 Hz), 6.45 (2H, b), 6.40–6.36 (1H, d, J = 15.7 Hz), 3.76 (3H, s), 3.72 (3H, s); ^{13}C NMR (100 MHz, $CDCl_3$) δ 167.2, 166.4, 15267, 140.3, 132.6, 129.8, 117.1, 116.9, 116.1, 51.9, 51.8; FT-IR (KBr) $\tilde{\nu}$ 3446, 3360, 3254, 1687, 1659, 1606, 1563, 1505, 1428, 1342, 1298, 1258, 1174, 1106, 980, 754, 484 cm^{-1}; LRMS-EI$^+$ (*m/z*) 258.45 ([M+Na]$^+$, 100), 236.27 ([M+H]$^+$, 42) 189.98 (11.71); HRMS-TOF-ES$^+$ (*m/*z) [M+Na]$^+$ calcd for $C_{12}H_{12}NNaO_4$, 258.0742 found 258.0742.

Methyl (*E*)-3-(2-amino-5-chlorophenyl) acrylate (ii).

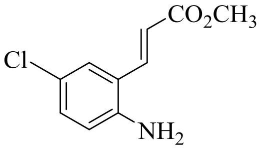

The reaction was carried out as described for the synthesis of (*E*)-methyl 4-amino-3-(3-methoxy-3-oxoprop-1-en-1-yl) benzoate (**i**) using 4-chloro-2-iodoaniline (3.95 mmol). The residue was purified by column chromatography (diethyl ether/petroleum ether, 1:4) to give (*E*)-methyl 4-amino-3-(3-methoxy-3-oxoprop-1-en-1-yl)benzoate (2.51 mmol) as yellow solid. Mp 182–184°C; ^{1}H NMR (400 MHz, $CDCl_3$) δ 7.73 (1H, d, J = 15.8 Hz), 7.34 (1H, d, J = 2.2 Hz), 7.12 (1H, dd, J = 2.2, 8.6 Hz), 6.65–6.62 (1H, d, J = 8.6 Hz), 6.35 (1H, d, J = 15.8 Hz), 3.94 (3H, m), 3.81 (3H, m); ^{13}C NMR (100 MHz, $CDCl_3$) δ 167.2, 144.0, 138.8, 131.0, 127.4, 123.8, 121.2, 119.1, 117.9, 51.7; LRMS-EI$^+$ (*m/z*) 211.0 ([M] $^+$); FT-IR (KBr) $\tilde{\nu}$ 3412, 3340, 2929, 1717, 1636, 1493, 1436, 1376, 1302, 1204, 1174, 1063, 823, 648, 5415 cm^{-1}.

tert-Butyl (E)-3-(2-aminophenyl)acrylate (iii).

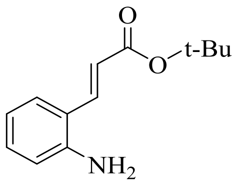

The reaction was carried out as described for the synthesis of (*E*)-methyl 4-amino-3-(3-methoxy-3-oxoprop-1-en-1-yl) benzoate **(i)** using 2-iodoaniline (9.17 mmol).The residue was purified by column chromatography (diethyl ether/petroleum ether, 1:4) to give (*E*)-*tert*-butyl 3-(2-aminophenyl) acrylate (6.85 mmol) as yellow solid. Mp 82–84 °C; ^{1}H NMR (400 MHz, $CDCl_3$) δ 7.72 (1H, d, J = 15.8 Hz), 7.37 (1H, d, J = 7.8 Hz), 7.16 (1H, t, J = 7.6 Hz), 6.76 (1H, t, J = 7.5 Hz), 6.69 (1H, d, J = 8.1 Hz), 6.29 (1H, d, J = 15.7 Hz), 3.94 (2H, b), 1.54 (9H, s); ^{13}C NMR (100 MHz, $CDCl_3$) δ 166.6, 145.4, 139.0, 131.0, 128.2, 120.3, 120.2, 118.9, 116.6, 80.4, 28.2. EI$^+$ (*m/z*) 219.1 ([M]$^+$); FT-IR (KB,) $\tilde{\nu}$ 3375, 2978, 2931, 1697, 1623, 1573, 1490, 1459, 1367, 1256, 1206, 1169, 981, 865, 750, 598 cm^{-1}.

(E)-Methyl 3-(2-aminophenyl) acrylate (iv).

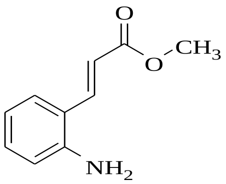

The reaction was carried out as described for the synthesis of (*E*)-methyl 4-amino-3-(3-methoxy-3-oxoprop-1-en-1-yl) benzoate **(i)** using 2-iodoaniline (9.17 mmol). Yellow solid; Mp 62–65 °C; H^1 NMR (400 MHz, $CDCl_3$) δ 7.83 (1H, d, J = 15.8 Hz), 7.39–7.36 (1H, m), 7.19–7.15 (1H, m), 6.78–6.69 (2H, m), 6.35 (1H, d, J = 15.8 Hz), 3.99 (2H, b), 3.80 (3H, m); ^{13}C NMR (100 MHz, $CDCl_3$) δ 167.8, 145.0, 140.3, 131.4, 128.1, 119.8, 118.9, 117.6, 116.8, 51.7. EI$^+$ (*m/z*) 177.07 ([M]$^+$); FT-IR (KBr) $\tilde{\nu}$ 3377, 3240, 2951, 1703, 1623, 1573, 1490, 1460, 1434, 1265, 1198, 11697, 1035, 9817, 860, 758, 598 cm^{-1}.

(E)-3-(2-Aminophenyl)-1-phenylprop-2-en-1-one (**v**)[1]

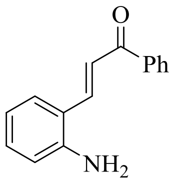

General Procedure for preparing benzylidene amino phenyl acrylate[2]

To the stirred solution of *(E)*-2-aminophenyl acrylate derivatives (1.0 equiv) and aldehyde (1.0 equiv) in anhydrous toluene (10 mL) added 4 Å molecular sieves (5.0 equiv). The reaction

mixture was stirred at 110 °C for 18 h. After completion of the reaction molecular sieves were filtered and solvent was removed to obtain the substituted benzylidene amino phenyl acrylate.

(*E*)-*tert*-Butyl 3-(2-((*E*)-(4-nitrobenzylidene)amino)phenyl)acrylate (vi)

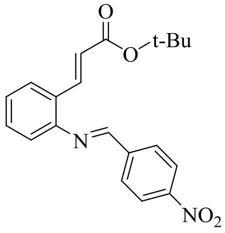

Yellow solid; Mp 143–145 °C; ¹H NMR (400 MHz, $CDCl_3$) δ 8.49 (1H, s), 8.34 (2H, d, *J* = 8.7 Hz), 8.20–8.11 (3H, m), 7.67 (1H, d, *J* = 7.2 Hz), 7.45–7.40 (1H, t, *J* = 7.5 Hz), 7.30 (1H, t, *J* = 7.5 Hz), 7.04 (1H, d, *J* = 7.4 Hz), 6.40 (1H, d, *J* = 16.1 Hz), 1.52 (9H, m); ^{13}C NMR (100 MHz, $CDCl_3$) δ 166.3, 157.9, 149.9, 149.5, 141.3, 139.6, 131.0, 130.0, 129.1, 124.1, 121.3, 118.2, 80.5, 28.2.

(*E*)-Methyl 3-(2-((*E*)-(4-nitrobenzylidene) amino) phenyl) acrylate (vii)

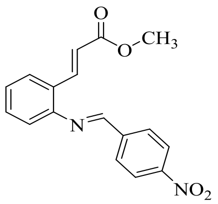

Yellow solid; Mp 62–65 °C; ¹H NMR (400 MHz, $CDCl_3$) δ 8.50 (1H, s), 8.35 (2H, d, *J* = 8.6 Hz), 8.20 (1H, d, *J* = 16.2 Hz), 8.12 (2H, d, *J* = 8.5 Hz), 7.67 (1H, d, *J* = 7.6 Hz), 7.44 (1H, t, *J* = 7.3 Hz), 7.32 (1H, t, *J* = 7.4 Hz), 7.04 (1H, *J* = 7.8 Hz), 6.47 (1H, d, *J* = 16.1 Hz), 3.80 (3H, s); ^{13}C NMR (100 MHz, $CDCl_3$) δ 167.5, 158.1, 150.2, 149.5, 141.2, 141.1, 131.3, 129.7, 128.7, 128.9, 127.6, 127.2, 124.1, 119.3, 118.4, 51.7.

General procedure for synthesis Knoevenagel intermediate

Ethyl (*Z*)-2-cyano-3-(2,4,6-trimethoxyphenyl)acrylate (5a1): To the stirred solution of ethyl cyanoacetate (1.02 mmol) and 2,4,6 trimethoxybenzaldehyde (1.02 mmol) in anhydrous DCM (10 mL) added (0.51 mmol) of DBU. The reaction mixture was stirred at room temperature and monitored by TLC for completion. Reaction mixture was concentrated using reduced pressure and purified using silica gel column chromatography to yield substituted Knoevenagel product. Yellow solid; Mp 101–102 °C; ¹H NMR (400 MHz, $CDCl_3$) δ 8.41 (1H, s), 6.09 (2H, s), 4.33 (2H, q, *J* = 7.0 Hz), 3.88 (6H, s), 3.86 (3H, s), 1.36 (3H, t, *J* = 7.0 Hz); ^{13}C NMR (100 MHz, $CDCl_3$) δ 165.6, 164.0, 16.2, 147.4, 116.1, 104.6, 103.4, 90.4, 62.1, 55.6, 55.4, 14.3. LRMS-EI$^+$ (*m/z*) 292.32 ($[M+H]^+$, 100), ($[M+H]^+$ 246.37 (38.35); HRMS-TOF-ES$^+$ (*m/z*) $[M+Na]^+$ calcd for $C_{15}H_{17}NNaO_5$, 314.1004, found 314.1004.

Reference.

[1] G. T. Cin, S. Demirel and A. Cakici, Synthesis of novel ferrocenyl-containing pyrazolo[4,3-c]quinolines. *J. Organometallic Chem.* 2011**, 696** , 613.

[2] A. Patra, S. Mukherjee, T. K. Das, S. Jain, R. G. Gonnade, A. T. Biju, *N*-Heterocyclic-Carbene-Catalyzed Umpolung of Imines. *Angew. Chem. Int. Ed.* 2017**, 56**, 2730.

Crystallographic data of 3a

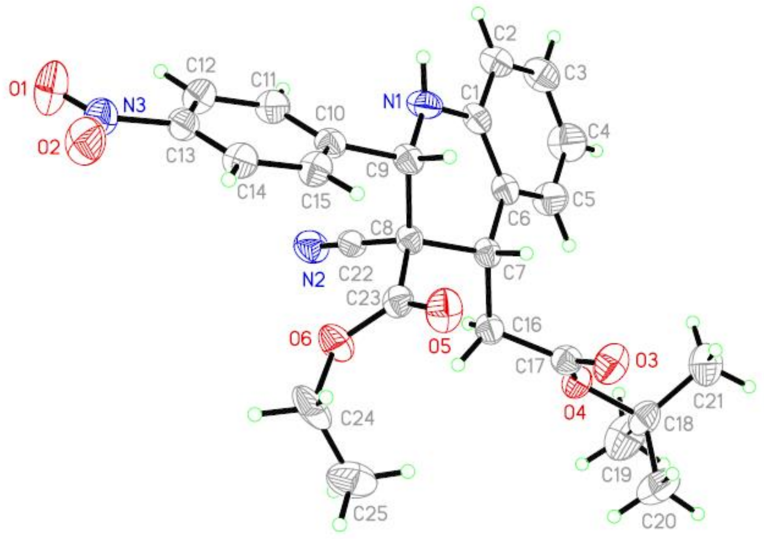

Fig. : The molecular structure of IC18499, thermal ellipsoids drawn at the 50% probability level.

Table 1. Crystal data and structure refinement for ic18499.

Identification code	ic18499	
Empirical formula	C25 H27 N3 O6	
Formula weight	465.49	
Temperature	200(2) K	
Wavelength	1.54178 Å	
Crystal system	Monoclinic	
Space group	$P2_1/c$	
Unit cell dimensions	a = 11.8148(3) Å	= 90°.

	b = 9.6634(2) Å	= 98.5727(10)°.
	c = 20.8234(5) Å	= 90°.
Volume	2350.87(10) $Å^3$	
Z	4	
Density (calculated)	1.315 Mg/m^3	
Absorption coefficient	0.784 mm^{-1}	
F(000)	984	
Crystal size	0.159 x 0.045 x 0.042 mm^3	
Theta range for data collection	4.294 to 69.990°.	
Index ranges	-14<=h<=14, -9<=k<=11, -24<=l<=25	
Reflections collected	11420	
Independent reflections	4394 [R(int) = 0.0208]	
Completeness to theta = 67.679°	98.8 %	
Absorption correction	Semi-empirical from equivalents	
Max. and min. transmission	0.7533 and 0.6024	
Refinement method	Full-matrix least-squares on F^2	
Data / restraints / parameters	4394 / 16 / 323	
Goodness-of-fit on F^2	1.037	
Final R indices [I>2sigma(I)]	R1 = 0.0392, wR2 = 0.0947	
R indices (all data)	R1 = 0.0481, wR2 = 0.1040	
Extinction coefficient	n/a	
Largest diff. peak and hole	0.248 and -0.174 e.$Å^{-3}$	

Table 2. Atomic coordinates (x 10^4) and equivalent isotropic displacement parameters ($Å^2$x 10^3)

for ic18499. U(eq) is defined as one third of the trace of the orthogonalized U^{ij} tensor.

	x	y	z	U(eq)
O(1)	9079(1)	3230(2)	1826(1)	60(1)
O(2)	10128(1)	4781(2)	2361(1)	55(1)
O(3)	3844(1)	12926(1)	1363(1)	45(1)
O(4)	2813(1)	12513(1)	374(1)	33(1)
O(5)	6564(1)	10539(1)	1915(1)	45(1)
O(6)	6956(1)	9505(1)	1013(1)	43(1)
N(1)	4210(1)	7188(2)	1979(1)	38(1)
N(2)	4930(1)	7391(2)	344(1)	40(1)
N(3)	9204(1)	4350(2)	2098(1)	40(1)
C(1)	3173(1)	7820(2)	1748(1)	31(1)
C(2)	2154(1)	7126(2)	1809(1)	36(1)
C(3)	1112(1)	7671(2)	1548(1)	43(1)
C(4)	1061(1)	8911(2)	1218(1)	51(1)
C(5)	2060(1)	9613(2)	1162(1)	42(1)
C(6)	3127(1)	9095(2)	1426(1)	29(1)
C(7)	4220(1)	9888(2)	1386(1)	27(1)
C(8)	5240(1)	8866(2)	1410(1)	26(1)
C(9)	5289(1)	7919(2)	2023(1)	30(1)

C(10)	6293(1)	6916(2)	2070(1)	30(1)
C(11)	6178(1)	5597(2)	1805(1)	35(1)
C(12)	7126(1)	4738(2)	1824(1)	37(1)
C(13)	8184(1)	5230(2)	2105(1)	33(1)
C(14)	8324(1)	6519(2)	2385(1)	35(1)
C(15)	7369(1)	7355(2)	2370(1)	34(1)
C(16)	4158(1)	10842(2)	792(1)	31(1)
C(17)	3595(1)	12218(2)	888(1)	31(1)
C(18)	2181(1)	13841(2)	320(1)	40(1)
C(19)	1508(2)	13728(2)	-358(1)	65(1)
C(20)	3005(2)	15043(2)	363(1)	58(1)
C(21)	1400(2)	13883(2)	837(1)	59(1)
C(22)	5093(1)	8019(2)	812(1)	28(1)
C(23)	6337(1)	9731(2)	1477(1)	31(1)
C(24)	8045(5)	10212(7)	979(3)	56(1)
C(25)	7800(3)	11555(5)	618(2)	57(1)
C(24')	7978(8)	10401(11)	1150(4)	56(1)
C(25')	8292(5)	10842(7)	515(2)	57(1)

Table 3. Bond lengths [Å] and angles [°] for ic18499.

O(1)-N(3)	1.220(2)
O(2)-N(3)	1.2197(19)
O(3)-C(17)	1.2028(19)
O(4)-C(17)	1.3355(17)
O(4)-C(18)	1.4807(19)
O(5)-C(23)	1.1998(19)
O(6)-C(23)	1.3144(18)
O(6)-C(24)	1.468(6)
O(6)-C(24')	1.478(8)
N(1)-C(1)	1.3894(19)
N(1)-C(9)	1.4477(19)
N(2)-C(22)	1.140(2)
N(3)-C(13)	1.4767(19)
C(1)-C(6)	1.399(2)
C(1)-C(2)	1.401(2)
C(2)-C(3)	1.374(2)
C(3)-C(4)	1.378(3)
C(4)-C(5)	1.382(2)
C(5)-C(6)	1.392(2)
C(6)-C(7)	1.5136(19)
C(7)-C(16)	1.5360(19)
C(7)-C(8)	1.5532(18)

C(8)-C(22)	1.4779(19)
C(8)-C(23)	1.5313(19)
C(8)-C(9)	1.5648(19)
C(9)-C(10)	1.5235(19)
C(10)-C(11)	1.388(2)
C(10)-C(15)	1.396(2)
C(11)-C(12)	1.390(2)
C(12)-C(13)	1.383(2)
C(13)-C(14)	1.375(2)
C(14)-C(15)	1.384(2)
C(16)-C(17)	1.513(2)
C(18)-C(20)	1.510(3)
C(18)-C(19)	1.517(2)
C(18)-C(21)	1.520(3)
C(24)-C(25)	1.506(7)
C(24')-C(25')	1.488(7)

C(17)-O(4)-C(18)	121.84(12)
C(23)-O(6)-C(24)	123.1(3)
C(23)-O(6)-C(24')	106.8(3)
C(1)-N(1)-C(9)	121.96(13)
O(2)-N(3)-O(1)	123.29(14)
O(2)-N(3)-C(13)	118.44(15)
O(1)-N(3)-C(13)	118.27(14)

N(1)-C(1)-C(6)	121.52(13)
N(1)-C(1)-C(2)	118.90(14)
C(6)-C(1)-C(2)	119.50(13)
C(3)-C(2)-C(1)	120.79(16)
C(2)-C(3)-C(4)	120.05(15)
C(3)-C(4)-C(5)	119.64(15)
C(4)-C(5)-C(6)	121.67(16)
C(5)-C(6)-C(1)	118.32(14)
C(5)-C(6)-C(7)	121.73(14)
C(1)-C(6)-C(7)	119.93(12)
C(6)-C(7)-C(16)	114.18(11)
C(6)-C(7)-C(8)	109.92(12)
C(16)-C(7)-C(8)	110.50(11)
C(22)-C(8)-C(23)	111.79(11)
C(22)-C(8)-C(7)	109.44(11)
C(23)-C(8)-C(7)	107.34(12)
C(22)-C(8)-C(9)	110.39(12)
C(23)-C(8)-C(9)	108.44(11)
C(7)-C(8)-C(9)	109.35(10)
N(1)-C(9)-C(10)	111.30(13)
N(1)-C(9)-C(8)	107.99(11)
C(10)-C(9)-C(8)	111.02(11)
C(11)-C(10)-C(15)	119.04(14)
C(11)-C(10)-C(9)	121.94(13)

C(15)-C(10)-C(9)	118.98(14)
C(10)-C(11)-C(12)	120.43(14)
C(13)-C(12)-C(11)	118.69(15)
C(14)-C(13)-C(12)	122.39(14)
C(14)-C(13)-N(3)	118.60(13)
C(12)-C(13)-N(3)	119.01(15)
C(13)-C(14)-C(15)	118.18(14)
C(14)-C(15)-C(10)	121.22(15)
C(17)-C(16)-C(7)	113.06(11)
O(3)-C(17)-O(4)	126.27(15)
O(3)-C(17)-C(16)	123.35(13)
O(4)-C(17)-C(16)	110.38(12)
O(4)-C(18)-C(20)	110.38(13)
O(4)-C(18)-C(19)	101.39(14)
C(20)-C(18)-C(19)	110.77(17)
O(4)-C(18)-C(21)	108.76(14)
C(20)-C(18)-C(21)	113.05(17)
C(19)-C(18)-C(21)	111.85(16)
N(2)-C(22)-C(8)	176.82(15)
O(5)-C(23)-O(6)	125.46(14)
O(5)-C(23)-C(8)	121.07(13)
O(6)-C(23)-C(8)	113.46(12)
O(6)-C(24)-C(25)	108.7(5)
O(6)-C(24')-C(25')	107.5(6)

Symmetry transformations used to generate equivalent atoms:

Table 4. Anisotropic displacement parameters ($Å^2 x 10^3$) for ic18499. The anisotropic displacement factor exponent takes the form: $-2\pi^2[h^2 a^{*2}U^{11} + ... + 2 h k a^* b^* U^{12}]$

	U^{11}	U^{22}	U^{33}	U^{23}	U^{13}	U^{12}
O(1)	50(1)	52(1)	78(1)	-16(1)	3(1)	17(1)
O(2)	29(1)	57(1)	77(1)	3(1)	3(1)	7(1)
O(3)	56(1)	40(1)	35(1)	-5(1)	-5(1)	6(1)
O(4)	32(1)	31(1)	33(1)	2(1)	-1(1)	5(1)
O(5)	37(1)	46(1)	51(1)	-16(1)	1(1)	-5(1)
O(6)	27(1)	54(1)	49(1)	-2(1)	13(1)	-3(1)
N(1)	29(1)	45(1)	40(1)	18(1)	9(1)	4(1)
N(2)	47(1)	41(1)	33(1)	-4(1)	10(1)	-7(1)
N(3)	36(1)	42(1)	44(1)	5(1)	8(1)	9(1)
C(1)	28(1)	40(1)	26(1)	0(1)	7(1)	2(1)
C(2)	34(1)	42(1)	34(1)	3(1)	8(1)	-3(1)
C(3)	27(1)	54(1)	47(1)	0(1)	6(1)	-6(1)
C(4)	25(1)	61(1)	64(1)	10(1)	0(1)	4(1)
C(5)	30(1)	43(1)	51(1)	9(1)	3(1)	4(1)
C(6)	26(1)	36(1)	26(1)	-1(1)	4(1)	4(1)
C(7)	26(1)	32(1)	24(1)	0(1)	4(1)	4(1)
C(8)	24(1)	31(1)	24(1)	0(1)	4(1)	2(1)
C(9)	28(1)	38(1)	25(1)	4(1)	4(1)	4(1)
C(10)	29(1)	36(1)	25(1)	7(1)	4(1)	4(1)

C(11)	30(1)	38(1)	37(1)	3(1)	1(1)	0(1)
C(12)	37(1)	33(1)	40(1)	0(1)	3(1)	2(1)
C(13)	30(1)	37(1)	33(1)	6(1)	6(1)	7(1)
C(14)	29(1)	40(1)	34(1)	4(1)	0(1)	2(1)
C(15)	34(1)	35(1)	31(1)	0(1)	-1(1)	3(1)
C(16)	31(1)	34(1)	27(1)	2(1)	5(1)	5(1)
C(17)	31(1)	34(1)	29(1)	4(1)	4(1)	1(1)
C(18)	36(1)	33(1)	48(1)	4(1)	-1(1)	9(1)
C(19)	64(1)	60(1)	60(1)	7(1)	-21(1)	22(1)
C(20)	60(1)	35(1)	77(1)	12(1)	2(1)	1(1)
C(21)	48(1)	56(1)	75(1)	2(1)	18(1)	17(1)
C(22)	27(1)	30(1)	29(1)	4(1)	7(1)	1(1)
C(23)	25(1)	32(1)	34(1)	2(1)	1(1)	4(1)
C(24)	28(1)	81(3)	66(4)	-11(2)	26(2)	-10(2)
C(25)	57(2)	63(3)	51(2)	0(2)	8(1)	-25(2)
C(24')	28(1)	81(3)	66(4)	-11(2)	26(2)	-10(2)
C(25')	57(2)	63(3)	51(2)	0(2)	8(1)	-25(2)

Table 5. Hydrogen coordinates (x 10^4) and isotropic displacement parameters ($Å^2$x 10^3) for ic18499.

	x	y	z	U(eq)
H(1)	4161(16)	6590(20)	2286(10)	46(5)
H(2A)	2183	6267	2033	44
H(3)	427	7194	1596	51
H(4)	342	9281	1030	61
H(5)	2017	10471	937	50
H(7)	4372	10485	1782	33
H(9)	5384	8516	2420	36
H(11)	5446	5279	1609	42
H(12)	7049	3830	1647	45
H(14)	9057	6827	2584	42
H(15)	7448	8244	2566	40
H(16A)	4943	11010	698	37
H(16B)	3724	10369	411	37
H(19A)	2035	13551	-671	97
H(19B)	1096	14596	-471	97
H(19C)	958	12966	-372	97
H(20A)	3418	15110	806	87
H(20B)	2580	15901	251	87
H(20C)	3553	14897	59	87

H(21A)	902	13067	794	88
H(21B)	929	14722	782	88
H(21C)	1864	13889	1268	88
H(24A)	8541	9621	750	67
H(24B)	8448	10396	1422	67
H(25A)	8514	12074	624	86
H(25B)	7261	12104	828	86
H(25C)	7465	11362	168	86
H(24C)	8618	9885	1403	67
H(24D)	7807	11219	1405	67
H(25D)	9020	11346	588	86
H(25E)	7692	11447	292	86
H(25F)	8370	10025	246	86

Crystallographic data of 4a

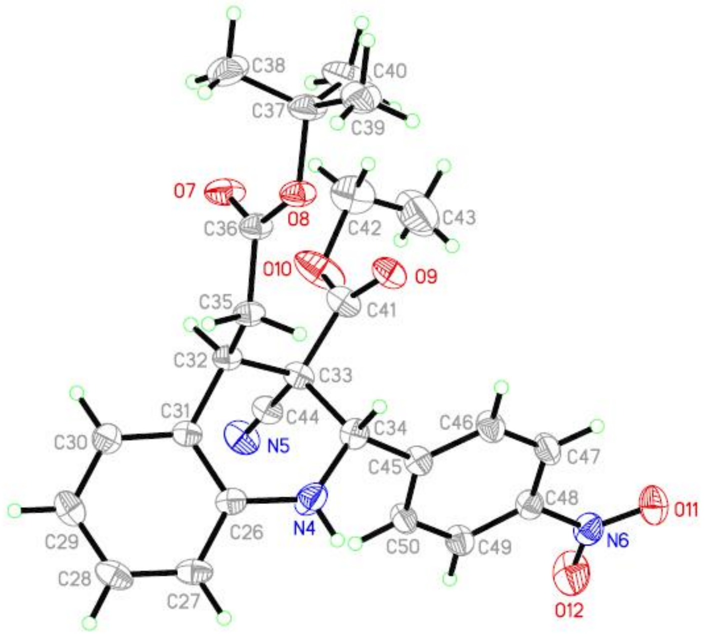

Fig. 2: The molecular structure of IC18555, thermal ellipsoids drawn at the 30% probability level.

Table 1. Crystal data and structure refinement for ic18555.

Identification code	ic18555	
Empirical formula	C25 H27 N3 O6	
Formula weight	465.49	
Temperature	200(2) K	
Wavelength	1.54178 Å	
Crystal system	Triclinic	
Space group	P-1	
Unit cell dimensions	a = 9.4969(2) Å	= 89.5889(12)°.
	b = 12.7230(3) Å	= 87.0347(11)°.
	c = 20.1047(5) Å	= 88.2917(12)°.
Volume	2424.86(10) Å^3	

Z	4
Density (calculated)	1.275 Mg/m^3
Absorption coefficient	0.760 mm^{-1}
F(000)	984
Crystal size	0.131 x 0.100 x 0.086 mm^3
Theta range for data collection	3.475 to 69.998°.
Index ranges	-11<=h<=11, -15<=k<=13, -24<=l<=24
Reflections collected	15799
Independent reflections	9164 [R(int) = 0.0158]
Completeness to theta = 67.679°	99.4 %
Absorption correction	Semi-empirical from equivalents
Max. and min. transmission	0.7533 and 0.6439
Refinement method	Full-matrix least-squares on F^2
Data / restraints / parameters	9164 / 6 / 644
Goodness-of-fit on F^2	1.029
Final R indices [I>2sigma(I)]	R1 = 0.0468, wR2 = 0.1169
R indices (all data)	R1 = 0.0559, wR2 = 0.1267
Extinction coefficient	n/a
Largest diff. peak and hole	0.474 and -0.507 e.$Å^{-3}$

Table 2. Atomic coordinates (x 10^4) and equivalent isotropic displacement parameters (Å^{2}x 10^3)

for ic18555. U(eq) is defined as one third of the trace of the orthogonalized U^{ij} tensor.

	x	y	z	U(eq)
O(1)	8547(2)	2594(2)	1016(1)	69(1)
O(2)	6354(1)	2236(1)	1410(1)	51(1)
O(3)	8697(1)	820(1)	2180(1)	51(1)
O(4)	10961(2)	934(1)	1819(1)	76(1)
O(5)	10604(2)	-2381(1)	4764(1)	57(1)
O(6)	12589(2)	-1652(1)	4894(1)	63(1)
N(1)	9689(2)	2938(1)	3706(1)	59(1)
N(2)	12747(2)	2591(1)	2639(1)	60(1)
N(3)	11411(2)	-1650(1)	4676(1)	44(1)
C(1)	9633(2)	3945(1)	3449(1)	45(1)
C(2)	9782(2)	4802(2)	3870(1)	55(1)
C(3)	9847(2)	5803(2)	3618(1)	62(1)
C(4)	9756(2)	5987(2)	2944(1)	60(1)
C(5)	9578(2)	5145(2)	2527(1)	51(1)
C(6)	9511(2)	4122(1)	2768(1)	41(1)
C(7)	9345(2)	3219(1)	2296(1)	40(1)
C(8)	10059(2)	2206(1)	2593(1)	38(1)
C(9)	9415(2)	2022(1)	3316(1)	40(1)

C(10)	7797(2)	3096(2)	2126(1)	43(1)
C(11)	7637(2)	2606(2)	1455(1)	46(1)
C(12)	5910(2)	1764(2)	787(1)	59(1)
C(13)	4411(3)	1467(3)	971(1)	88(1)
C(14)	5924(3)	2588(2)	243(1)	80(1)
C(15)	6839(4)	812(3)	614(2)	106(1)
C(16)	9817(2)	1233(2)	2179(1)	43(1)
C(17)	10906(5)	63(3)	1357(2)	63(1)
C(18)	11439(8)	-839(4)	1785(3)	103(2)
C(17')	10787(10)	-238(6)	1648(5)	63(1)
C(18')	12116(12)	-728(8)	1446(8)	103(2)
C(19)	11584(2)	2403(1)	2616(1)	42(1)
C(20)	9990(2)	1039(1)	3650(1)	37(1)
C(21)	9185(2)	144(1)	3682(1)	41(1)
C(22)	9645(2)	-746(1)	4014(1)	43(1)
C(23)	10929(2)	-728(1)	4301(1)	37(1)
C(24)	11771(2)	131(2)	4262(1)	46(1)
C(25)	11287(2)	1023(2)	3938(1)	47(1)
O(7)	6283(1)	7883(1)	1067(1)	60(1)
O(8)	8522(1)	7368(1)	1262(1)	49(1)
O(9)	6209(1)	5922(1)	2095(1)	55(1)
O(10)	3959(2)	6430(2)	1875(1)	50(1)
O(10')	3953(6)	5935(5)	2220(3)	50(1)
O(11)	3805(2)	2609(1)	4626(1)	69(1)

O(12)	2181(2)	3657(1)	5022(1)	82(1)
N(4)	6072(2)	7819(1)	3797(1)	60(1)
N(5)	2458(2)	7785(1)	3132(1)	56(1)
N(6)	3251(2)	3485(1)	4682(1)	51(1)
C(26)	5820(2)	8862(1)	3641(1)	39(1)
C(27)	5778(2)	9623(2)	4139(1)	46(1)
C(28)	5527(2)	10666(2)	3989(1)	50(1)
C(29)	5294(2)	10973(2)	3343(1)	52(1)
C(30)	5338(2)	10218(1)	2847(1)	45(1)
C(31)	5617(2)	9169(1)	2984(1)	36(1)
C(32)	5699(2)	8350(1)	2443(1)	37(1)
C(33)	5074(2)	7311(1)	2744(1)	39(1)
C(34)	5926(2)	6948(1)	3354(1)	43(1)
C(35)	7205(2)	8204(2)	2133(1)	40(1)
C(36)	7250(2)	7793(2)	1430(1)	44(1)
C(37)	8868(2)	6995(2)	576(1)	59(1)
C(38)	8834(3)	7927(2)	104(1)	84(1)
C(39)	10364(2)	6557(2)	623(1)	70(1)
C(40)	7894(3)	6130(3)	403(1)	87(1)
C(41)	5157(2)	6433(2)	2226(1)	50(1)
C(42)	3910(3)	5639(2)	1341(1)	61(1)
C(43)	3431(5)	4662(3)	1636(3)	91(1)
C(42')	4084(11)	5002(8)	1805(5)	61(1)
C(43')	2801(15)	4821(15)	1449(10)	91(1)

C(44)	3588(2)	7562(1)	2958(1)	42(1)
C(45)	5244(2)	6030(1)	3718(1)	41(1)
C(46)	5741(2)	5010(2)	3596(1)	50(1)
C(47)	5092(2)	4169(1)	3908(1)	52(1)
C(48)	3940(2)	4367(1)	4339(1)	42(1)
C(49)	3441(2)	5368(1)	4483(1)	46(1)
C(50)	4114(2)	6202(1)	4173(1)	45(1)

Table 3. Bond lengths [Å] and angles [°] for ic18555.

O(1)-C(11)	1.203(2)
O(2)-C(11)	1.327(2)
O(2)-C(12)	1.481(2)
O(3)-C(16)	1.200(2)
O(4)-C(16)	1.321(2)
O(4)-C(17)	1.455(4)
O(4)-C(17')	1.548(7)
O(5)-N(3)	1.228(2)
O(6)-N(3)	1.223(2)
N(1)-C(1)	1.378(3)
N(1)-C(9)	1.447(2)
N(2)-C(19)	1.140(2)
N(3)-C(23)	1.464(2)
C(1)-C(6)	1.397(3)
C(1)-C(2)	1.399(3)
C(2)-C(3)	1.369(3)
C(3)-C(4)	1.381(3)
C(4)-C(5)	1.385(3)
C(5)-C(6)	1.387(3)
C(6)-C(7)	1.512(2)
C(7)-C(10)	1.539(2)
C(7)-C(8)	1.567(2)

C(8)-C(19)	1.480(2)
C(8)-C(16)	1.526(2)
C(8)-C(9)	1.566(2)
C(9)-C(20)	1.516(2)
C(10)-C(11)	1.507(2)
C(12)-C(15)	1.509(4)
C(12)-C(14)	1.509(3)
C(12)-C(13)	1.510(3)
C(17)-C(18)	1.520(7)
C(17')-C(18')	1.431(15)
C(20)-C(25)	1.388(2)
C(20)-C(21)	1.391(2)
C(21)-C(22)	1.381(3)
C(22)-C(23)	1.377(2)
C(23)-C(24)	1.374(2)
C(24)-C(25)	1.382(3)
O(7)-C(36)	1.204(2)
O(8)-C(36)	1.337(2)
O(8)-C(37)	1.479(2)
O(9)-C(41)	1.194(2)
O(10)-C(41)	1.369(3)
O(10)-C(42)	1.480(3)
O(10')-C(41)	1.324(6)
O(10')-C(42')	1.456(8)

O(11)-N(6)	1.222(2)
O(12)-N(6)	1.210(2)
N(4)-C(26)	1.378(2)
N(4)-C(34)	1.442(2)
N(5)-C(44)	1.140(2)
N(6)-C(48)	1.466(2)
C(26)-C(31)	1.396(2)
C(26)-C(27)	1.396(2)
C(27)-C(28)	1.375(3)
C(28)-C(29)	1.379(3)
C(29)-C(30)	1.388(3)
C(30)-C(31)	1.382(2)
C(31)-C(32)	1.508(2)
C(32)-C(35)	1.537(2)
C(32)-C(33)	1.569(2)
C(33)-C(44)	1.480(2)
C(33)-C(41)	1.532(2)
C(33)-C(34)	1.563(2)
C(34)-C(45)	1.518(2)
C(35)-C(36)	1.509(2)
C(37)-C(40)	1.513(4)
C(37)-C(38)	1.515(3)
C(37)-C(39)	1.518(3)
C(42)-C(43)	1.449(5)

C(42')-C(43')	1.470(9)
C(45)-C(50)	1.386(2)
C(45)-C(46)	1.387(2)
C(46)-C(47)	1.383(3)
C(47)-C(48)	1.378(3)
C(48)-C(49)	1.373(2)
C(49)-C(50)	1.383(3)
C(11)-O(2)-C(12)	121.45(14)
C(16)-O(4)-C(17)	119.9(2)
C(16)-O(4)-C(17')	106.6(4)
C(1)-N(1)-C(9)	122.66(16)
O(6)-N(3)-O(5)	122.97(16)
O(6)-N(3)-C(23)	118.62(15)
O(5)-N(3)-C(23)	118.40(14)
N(1)-C(1)-C(6)	120.98(16)
N(1)-C(1)-C(2)	119.67(17)
C(6)-C(1)-C(2)	119.24(18)
C(3)-C(2)-C(1)	120.8(2)
C(2)-C(3)-C(4)	120.52(19)
C(3)-C(4)-C(5)	118.97(19)
C(4)-C(5)-C(6)	121.73(19)
C(5)-C(6)-C(1)	118.70(17)
C(5)-C(6)-C(7)	120.34(16)

C(1)-C(6)-C(7)	120.95(16)
C(6)-C(7)-C(10)	111.83(14)
C(6)-C(7)-C(8)	108.67(13)
C(10)-C(7)-C(8)	114.50(14)
C(19)-C(8)-C(16)	110.80(14)
C(19)-C(8)-C(9)	109.67(14)
C(16)-C(8)-C(9)	108.50(14)
C(19)-C(8)-C(7)	107.12(13)
C(16)-C(8)-C(7)	111.95(13)
C(9)-C(8)-C(7)	108.77(13)
N(1)-C(9)-C(20)	109.98(14)
N(1)-C(9)-C(8)	107.78(14)
C(20)-C(9)-C(8)	113.83(13)
C(11)-C(10)-C(7)	113.34(14)
O(1)-C(11)-O(2)	125.33(17)
O(1)-C(11)-C(10)	123.60(17)
O(2)-C(11)-C(10)	111.02(14)
O(2)-C(12)-C(15)	109.64(17)
O(2)-C(12)-C(14)	109.31(19)
C(15)-C(12)-C(14)	113.1(2)
O(2)-C(12)-C(13)	102.36(17)
C(15)-C(12)-C(13)	111.9(3)
C(14)-C(12)-C(13)	110.0(2)
O(3)-C(16)-O(4)	125.44(17)

O(3)-C(16)-C(8)	122.52(15)
O(4)-C(16)-C(8)	112.02(15)
O(4)-C(17)-C(18)	100.5(4)
C(18')-C(17')-O(4)	111.0(8)
N(2)-C(19)-C(8)	177.6(2)
C(25)-C(20)-C(21)	119.32(16)
C(25)-C(20)-C(9)	121.39(15)
C(21)-C(20)-C(9)	119.26(15)
C(22)-C(21)-C(20)	120.65(15)
C(23)-C(22)-C(21)	118.34(15)
C(24)-C(23)-C(22)	122.54(16)
C(24)-C(23)-N(3)	118.33(15)
C(22)-C(23)-N(3)	119.12(15)
C(23)-C(24)-C(25)	118.52(16)
C(24)-C(25)-C(20)	120.57(16)
C(36)-O(8)-C(37)	120.80(14)
C(41)-O(10)-C(42)	116.93(18)
C(41)-O(10')-C(42')	111.6(6)
C(26)-N(4)-C(34)	125.29(16)
O(12)-N(6)-O(11)	122.71(17)
O(12)-N(6)-C(48)	118.91(16)
O(11)-N(6)-C(48)	118.35(17)
N(4)-C(26)-C(31)	120.45(16)
N(4)-C(26)-C(27)	120.13(16)

C(31)-C(26)-C(27)	119.42(16)
C(28)-C(27)-C(26)	120.64(17)
C(27)-C(28)-C(29)	120.34(17)
C(28)-C(29)-C(30)	119.13(18)
C(31)-C(30)-C(29)	121.55(17)
C(30)-C(31)-C(26)	118.89(15)
C(30)-C(31)-C(32)	121.74(15)
C(26)-C(31)-C(32)	119.37(15)
C(31)-C(32)-C(35)	111.81(13)
C(31)-C(32)-C(33)	107.97(13)
C(35)-C(32)-C(33)	113.58(14)
C(44)-C(33)-C(41)	110.56(14)
C(44)-C(33)-C(34)	110.41(14)
C(41)-C(33)-C(34)	108.76(14)
C(44)-C(33)-C(32)	106.66(14)
C(41)-C(33)-C(32)	110.85(14)
C(34)-C(33)-C(32)	109.59(13)
N(4)-C(34)-C(45)	110.94(15)
N(4)-C(34)-C(33)	109.94(14)
C(45)-C(34)-C(33)	111.56(14)
C(36)-C(35)-C(32)	113.26(13)
O(7)-C(36)-O(8)	125.38(16)
O(7)-C(36)-C(35)	123.94(15)
O(8)-C(36)-C(35)	110.61(14)

O(8)-C(37)-C(40)	110.23(18)
O(8)-C(37)-C(38)	108.89(18)
C(40)-C(37)-C(38)	113.8(2)
O(8)-C(37)-C(39)	102.38(16)
C(40)-C(37)-C(39)	110.3(2)
C(38)-C(37)-C(39)	110.6(2)
O(9)-C(41)-O(10')	116.7(3)
O(9)-C(41)-O(10)	125.32(17)
O(9)-C(41)-C(33)	122.96(16)
O(10')-C(41)-C(33)	111.1(3)
O(10)-C(41)-C(33)	111.01(15)
C(43)-C(42)-O(10)	108.5(3)
O(10')-C(42')-C(43')	112.4(10)
N(5)-C(44)-C(33)	177.76(19)
C(50)-C(45)-C(46)	119.44(16)
C(50)-C(45)-C(34)	120.44(15)
C(46)-C(45)-C(34)	120.12(16)
C(47)-C(46)-C(45)	120.39(17)
C(48)-C(47)-C(46)	118.55(16)
C(49)-C(48)-C(47)	122.48(16)
C(49)-C(48)-N(6)	118.19(16)
C(47)-C(48)-N(6)	119.28(16)
C(48)-C(49)-C(50)	118.26(16)
C(49)-C(50)-C(45)	120.82(16)

Symmetry transformations used to generate equivalent atoms:

Table 4. Anisotropic displacement parameters ($Å^2 x 10^3$) for ic18555. The anisotropic displacement factor exponent takes the form: -2 ^{2}[$h^2 a^{*2}U^{11}$ + ... + 2 h k a* b* U^{12}]

	U^{11}	U^{22}	U^{33}	U^{23}	U^{13}	U^{12}
O(1)	47(1)	122(1)	37(1)	-13(1)	4(1)	-12(1)
O(2)	42(1)	75(1)	36(1)	-6(1)	-6(1)	-9(1)
O(3)	45(1)	56(1)	53(1)	-11(1)	-3(1)	-11(1)
O(4)	43(1)	94(1)	90(1)	-54(1)	4(1)	1(1)
O(5)	74(1)	46(1)	51(1)	3(1)	-7(1)	-8(1)
O(6)	60(1)	61(1)	69(1)	-6(1)	-23(1)	9(1)
N(1)	101(1)	43(1)	32(1)	-5(1)	-1(1)	-1(1)
N(2)	41(1)	64(1)	74(1)	10(1)	-8(1)	-10(1)
N(3)	50(1)	44(1)	37(1)	-10(1)	-2(1)	3(1)
C(1)	46(1)	43(1)	45(1)	-6(1)	-5(1)	0(1)
C(2)	60(1)	51(1)	56(1)	-14(1)	-14(1)	3(1)
C(3)	56(1)	45(1)	87(2)	-17(1)	-20(1)	2(1)
C(4)	50(1)	40(1)	92(2)	1(1)	-14(1)	-1(1)
C(5)	41(1)	50(1)	61(1)	8(1)	-6(1)	-1(1)
C(6)	33(1)	45(1)	45(1)	-2(1)	-3(1)	-3(1)
C(7)	35(1)	49(1)	34(1)	1(1)	1(1)	-4(1)
C(8)	33(1)	45(1)	35(1)	-4(1)	0(1)	-5(1)
C(9)	42(1)	43(1)	35(1)	-4(1)	4(1)	-2(1)
C(10)	36(1)	59(1)	36(1)	-4(1)	-1(1)	-1(1)

C(11)	38(1)	66(1)	35(1)	-1(1)	-3(1)	-1(1)
C(12)	56(1)	82(2)	42(1)	-10(1)	-16(1)	-6(1)
C(13)	74(2)	132(3)	64(2)	1(2)	-23(1)	-44(2)
C(14)	72(2)	122(2)	49(1)	12(1)	-23(1)	-18(2)
C(15)	112(2)	103(2)	108(2)	-52(2)	-49(2)	20(2)
C(16)	38(1)	52(1)	39(1)	-8(1)	-2(1)	-1(1)
C(17)	67(2)	72(3)	50(2)	-24(2)	0(2)	9(2)
C(18)	117(5)	60(2)	134(6)	-19(3)	-30(3)	5(3)
C(17')	67(2)	72(3)	50(2)	-24(2)	0(2)	9(2)
C(18')	117(5)	60(2)	134(6)	-19(3)	-30(3)	5(3)
C(19)	39(1)	46(1)	43(1)	2(1)	-2(1)	-5(1)
C(20)	36(1)	42(1)	34(1)	-4(1)	4(1)	-6(1)
C(21)	29(1)	49(1)	45(1)	1(1)	-1(1)	-7(1)
C(22)	37(1)	44(1)	48(1)	-2(1)	1(1)	-10(1)
C(23)	36(1)	41(1)	33(1)	-6(1)	3(1)	1(1)
C(24)	37(1)	56(1)	46(1)	-2(1)	-9(1)	-10(1)
C(25)	47(1)	48(1)	47(1)	1(1)	-10(1)	-18(1)
O(7)	46(1)	95(1)	38(1)	-14(1)	-7(1)	13(1)
O(8)	39(1)	73(1)	36(1)	-6(1)	3(1)	7(1)
O(9)	50(1)	56(1)	58(1)	-14(1)	4(1)	6(1)
O(10)	42(1)	55(1)	55(1)	-15(1)	-10(1)	-5(1)
O(10')	42(1)	55(1)	55(1)	-15(1)	-10(1)	-5(1)
O(11)	90(1)	37(1)	81(1)	6(1)	-2(1)	-7(1)
O(12)	72(1)	67(1)	104(1)	19(1)	28(1)	-13(1)

N(4)	92(1)	47(1)	45(1)	8(1)	-25(1)	-25(1)
N(5)	39(1)	59(1)	68(1)	-12(1)	6(1)	-4(1)
N(6)	57(1)	43(1)	54(1)	4(1)	-9(1)	-13(1)
C(26)	36(1)	45(1)	36(1)	-1(1)	1(1)	-14(1)
C(27)	40(1)	63(1)	36(1)	-8(1)	6(1)	-17(1)
C(28)	35(1)	60(1)	56(1)	-22(1)	6(1)	-7(1)
C(29)	42(1)	44(1)	71(1)	-12(1)	-6(1)	2(1)
C(30)	42(1)	48(1)	46(1)	-1(1)	-5(1)	1(1)
C(31)	29(1)	43(1)	35(1)	-4(1)	2(1)	-5(1)
C(32)	33(1)	45(1)	33(1)	-3(1)	-1(1)	-3(1)
C(33)	31(1)	44(1)	42(1)	-7(1)	1(1)	-4(1)
C(34)	38(1)	43(1)	48(1)	2(1)	-3(1)	-7(1)
C(35)	34(1)	54(1)	33(1)	-3(1)	0(1)	-4(1)
C(36)	37(1)	59(1)	36(1)	-4(1)	2(1)	1(1)
C(37)	52(1)	85(2)	38(1)	-12(1)	5(1)	15(1)
C(38)	87(2)	118(2)	44(1)	11(1)	18(1)	37(2)
C(39)	56(1)	94(2)	58(1)	-5(1)	13(1)	21(1)
C(40)	72(2)	114(2)	75(2)	-48(2)	1(1)	8(2)
C(41)	40(1)	51(1)	58(1)	-17(1)	3(1)	-6(1)
C(42)	52(1)	70(2)	61(2)	-10(1)	-9(1)	-9(1)
C(43)	81(3)	75(2)	119(4)	-27(2)	-2(2)	-16(2)
C(42')	52(1)	70(2)	61(2)	-10(1)	-9(1)	-9(1)
C(43')	81(3)	75(2)	119(4)	-27(2)	-2(2)	-16(2)
C(44)	37(1)	44(1)	45(1)	-7(1)	0(1)	-6(1)

C(45)	37(1)	39(1)	48(1)	-2(1)	-1(1)	-3(1)
C(46)	45(1)	45(1)	57(1)	-1(1)	11(1)	6(1)
C(47)	61(1)	34(1)	59(1)	-4(1)	5(1)	4(1)
C(48)	42(1)	38(1)	47(1)	1(1)	-5(1)	-8(1)
C(49)	41(1)	43(1)	51(1)	0(1)	6(1)	0(1)
C(50)	48(1)	34(1)	52(1)	-4(1)	6(1)	2(1)

Table 5. Hydrogen coordinates (x 10^4) and isotropic displacement parameters ($Å^2$x 10^3) for ic18555.

	x	y	z	U(eq)
H(1)	9590(20)	2855(19)	4127(13)	64(7)
H(2)	9840	4688	4335	66
H(3)	9956	6376	3910	74
H(4A)	9815	6681	2769	73
H(5A)	9499	5271	2064	61
H(7)	9887	3390	1872	48
H(9)	8371	1963	3294	48
H(10A)	7323	2655	2473	52
H(10B)	7316	3797	2135	52
H(13A)	4407	953	1336	133
H(13B)	3994	1161	584	133
H(13C)	3858	2097	1113	133
H(14A)	5362	3207	395	120
H(14B)	5523	2302	-155	120
H(14C)	6898	2790	135	120
H(15A)	7787	1038	473	159
H(15B)	6440	427	252	159
H(15C)	6895	353	1006	159

H(17A)	11531	169	954	76
H(17B)	9932	-47	1223	76
H(18A)	11458	-1493	1529	155
H(18B)	12394	-696	1916	155
H(18C)	10811	-909	2183	155
H(17C)	10377	-610	2042	76
H(17D)	10129	-291	1284	76
H(18D)	11978	-1467	1340	155
H(18E)	12516	-366	1051	155
H(18F)	12763	-685	1808	155
H(21)	8310	143	3474	49
H(22)	9090	-1355	4043	51
H(24)	12666	114	4452	55
H(25)	11847	1630	3913	56
H(4)	6220(30)	7653(19)	4200(13)	67(7)
H(27)	5923	9418	4586	55
H(28)	5514	11177	4331	60
H(29)	5106	11692	3240	63
H(30)	5172	10428	2403	54
H(32)	5079	8600	2084	44
H(34)	6891	6711	3184	51
H(35A)	7676	8887	2134	48
H(35B)	7742	7708	2413	48
H(38A)	7879	8242	114	127

H(38B)	9106	7692	-349	127
H(38C)	9496	8450	241	127
H(39A)	10958	7101	793	105
H(39B)	10736	6337	181	105
H(39C)	10364	5951	927	105
H(40A)	7861	5605	762	131
H(40B)	8245	5795	-13	131
H(40C)	6944	6429	348	131
H(42A)	3255	5887	1001	73
H(42B)	4859	5530	1120	73
H(43A)	3392	4129	1288	137
H(43B)	4087	4420	1969	137
H(43C)	2488	4776	1851	137
H(42C)	4290	4382	2088	73
H(42D)	4889	5078	1477	73
H(43D)	2939	4187	1177	137
H(43E)	2004	4730	1772	137
H(43F)	2603	5426	1161	137
H(46)	6533	4889	3294	60
H(47)	5433	3469	3828	62
H(49)	2654	5484	4787	55
H(50)	3797	6901	4273	54

^{1}H, ^{13}C and IR Spectra

Racemate of ethyl 4-(2-(*tert*-butoxy)-2-oxoethyl)-3-cyano-2-(4-nitrophenyl)-1,2,3,4-tetrahydroquinoline-3-carboxylate (**3a**, major isomer)

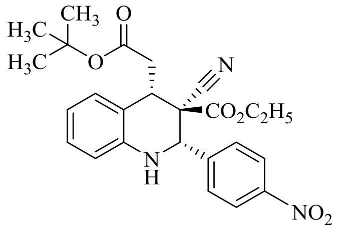

3a

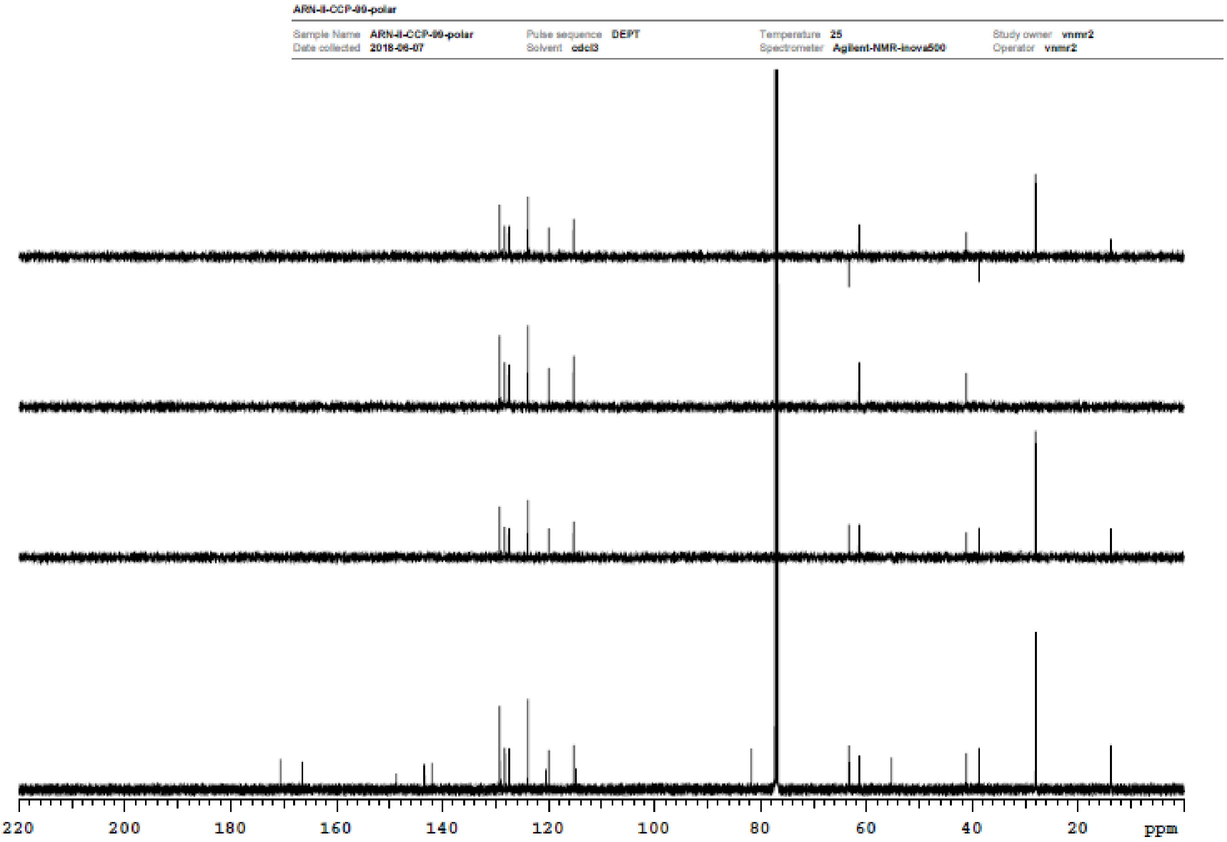

Racemate of ethyl 4-(2-(*tert*-butoxy)-2-oxoethyl)-3-cyano-2-(4-methoxyphenyl)-1,2,3,4-tetrahydroquinoline-3-carboxylate **(3b)**

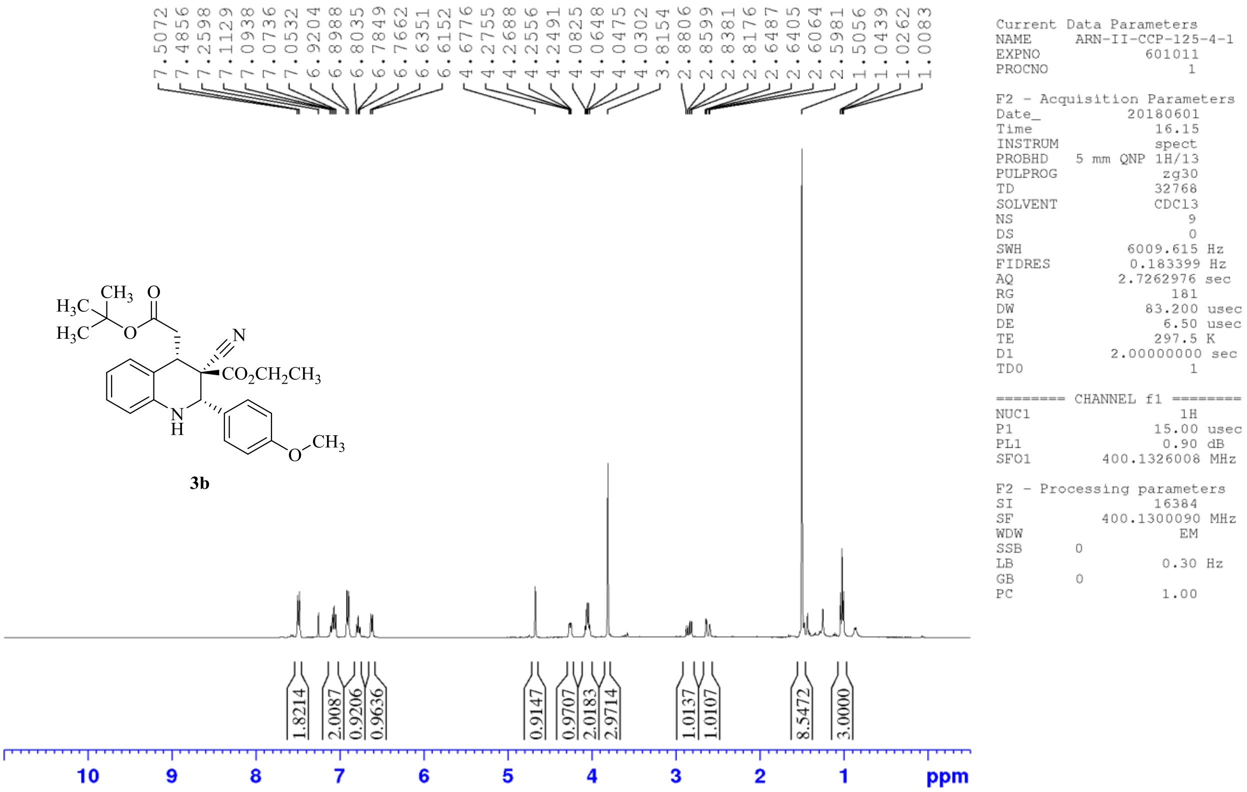

Racemate of ethyl 4-(2-(*tert*-butoxy)-2-oxoethyl)-3-cyano-2-(4-methoxyphenyl)-1,2,3,4-tetrahydroquinoline-3-carboxylate (**3b**)

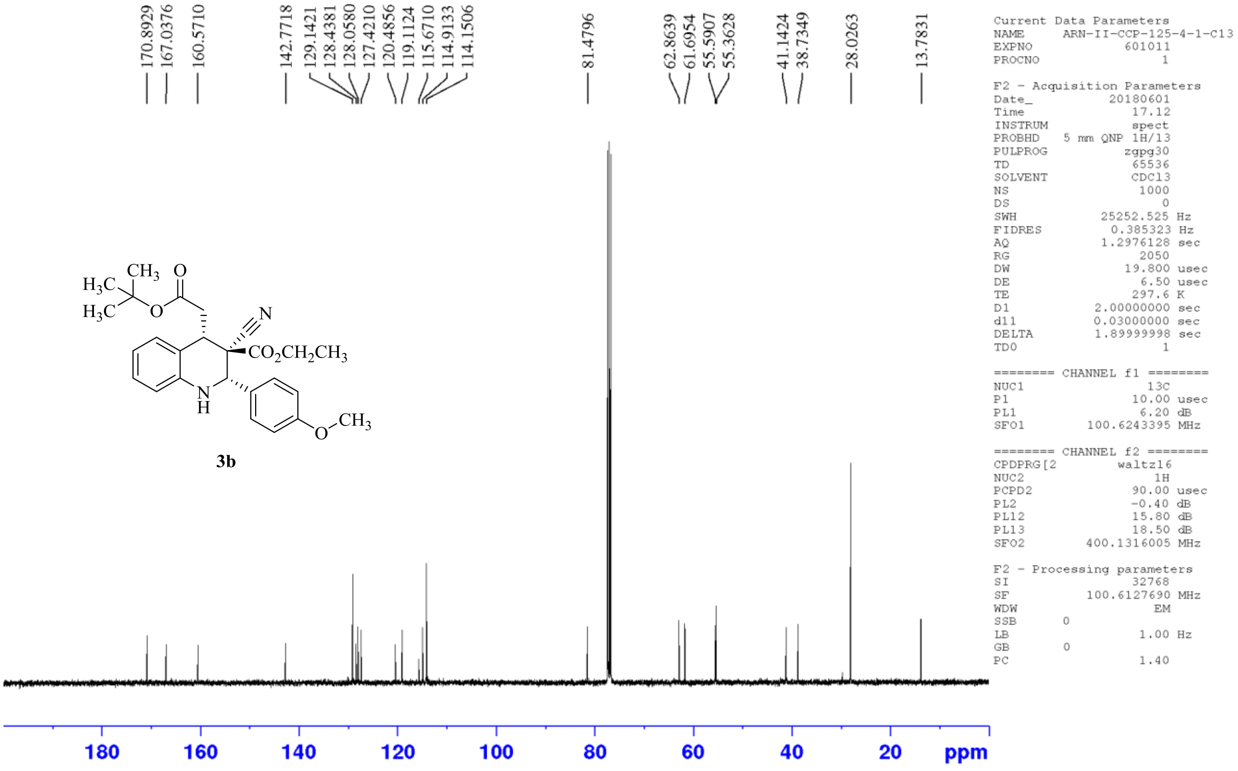

Racemate of ethyl 4-(2-(*tert*-butoxy)-2-oxoethyl)-3-cyano-2-phenyl-1,2,3,4-tetrahydroquinoline-3-carboxylate (**3c**)

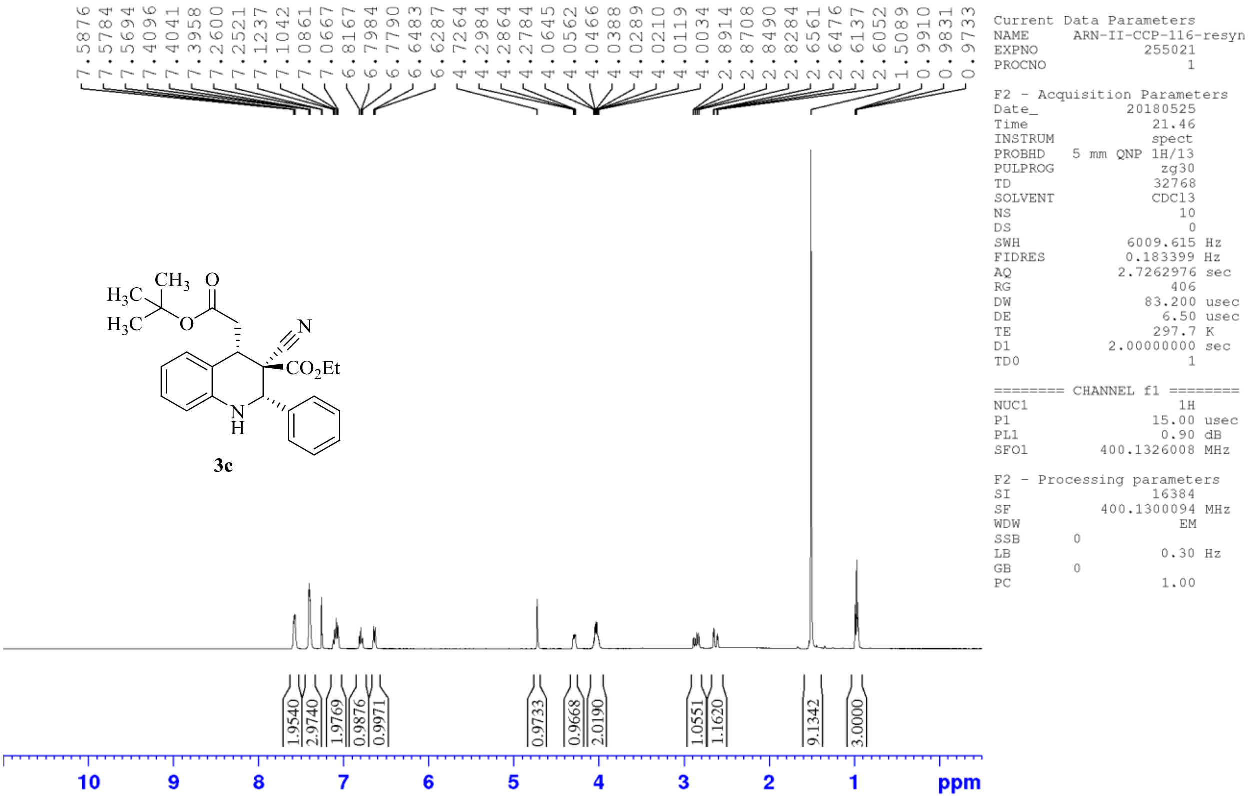

Racemate of ethyl 4-(2-(*tert*-butoxy)-2-oxoethyl)-3-cyano-2-phenyl-1,2,3,4-tetrahydroquinoline-3-carboxylate (**3c**)

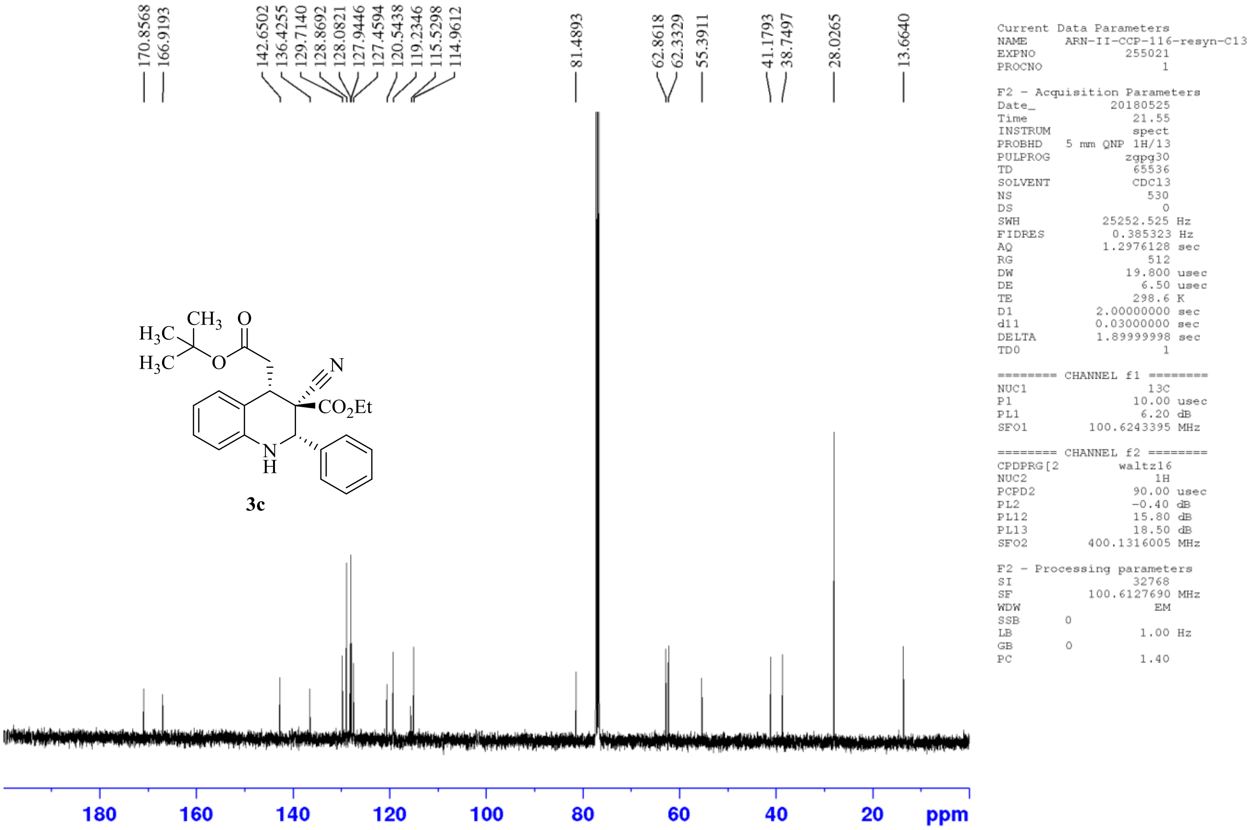

Ethyl 3-cyano-4-(2-methoxy-2-oxoethyl)-2-(4-nitrophenyl)-1,2,3,4-tetrahydroquinoline-3-carboxylate (**3d/4d,** mixture**)**

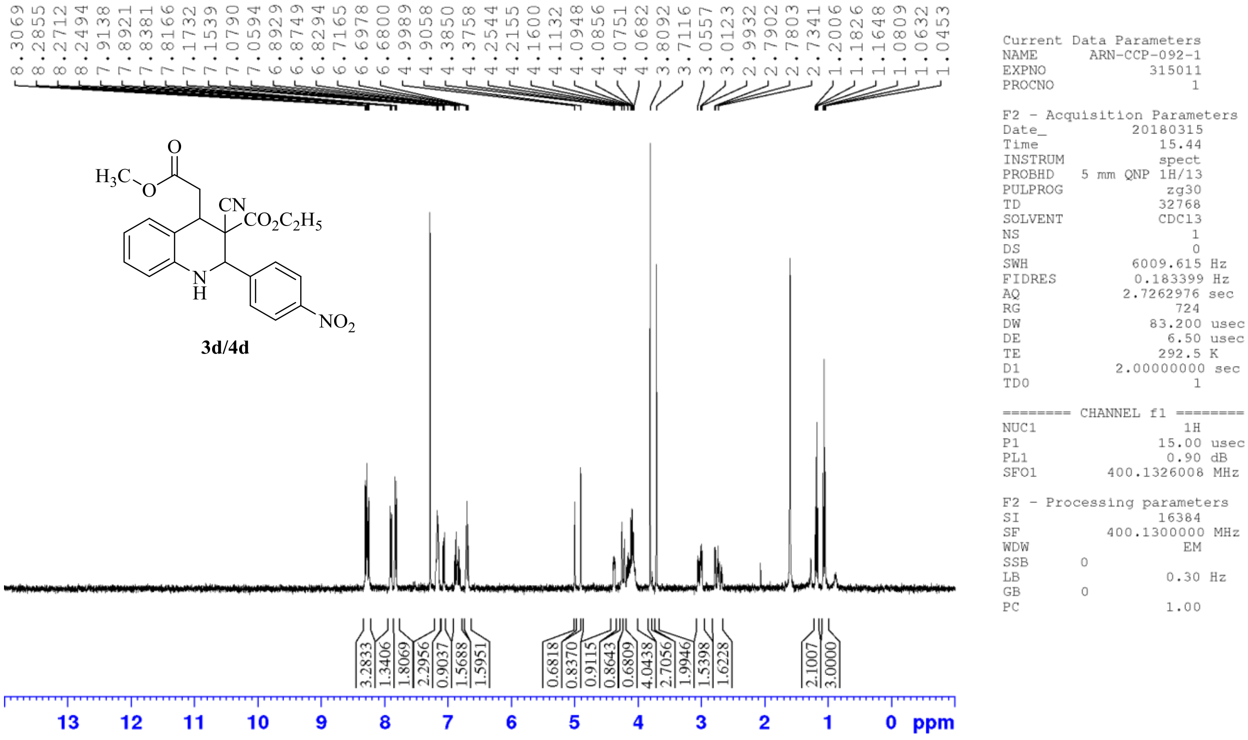

Racemate of ethyl 3-cyano-4-(2-methoxy-2-oxoethyl)-2-(4-nitrophenyl)-1,2,3,4-tetrahydroquinoline-3-carboxylate (**3d**, major isomer)

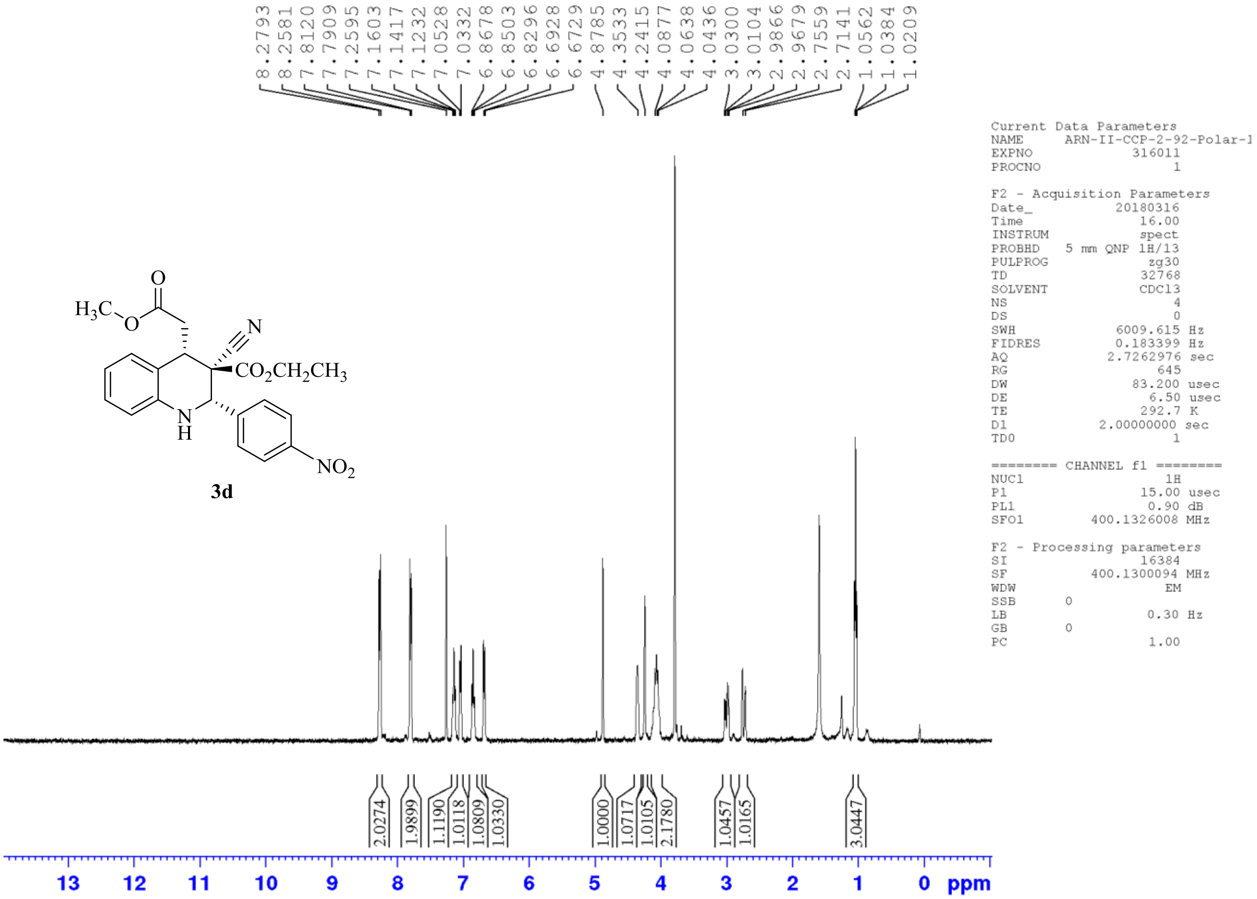

Racemate of ethyl 3-cyano-4-(2-methoxy-2-oxoethyl)-2-(4-nitrophenyl)-1,2,3,4-tetrahydroquinoline-3-carboxylate (**3d**, major isomer)

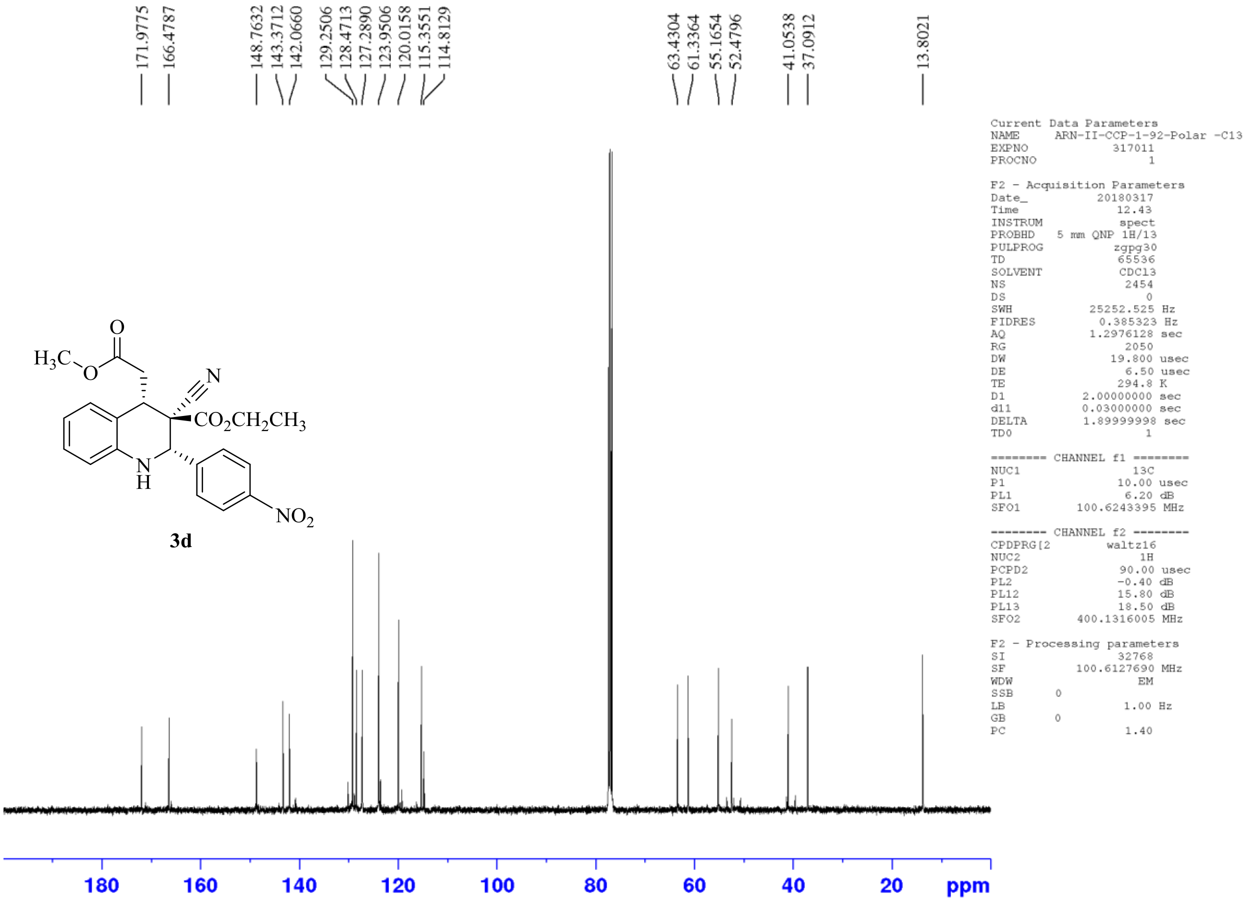

Racemate of ethyl 3-cyano-4-(2-methoxy-2-oxoethyl)-2-(2-methoxyphenyl)-1,2,3,4-tetrahydroquinoline-3-carboxylate (**3e**)

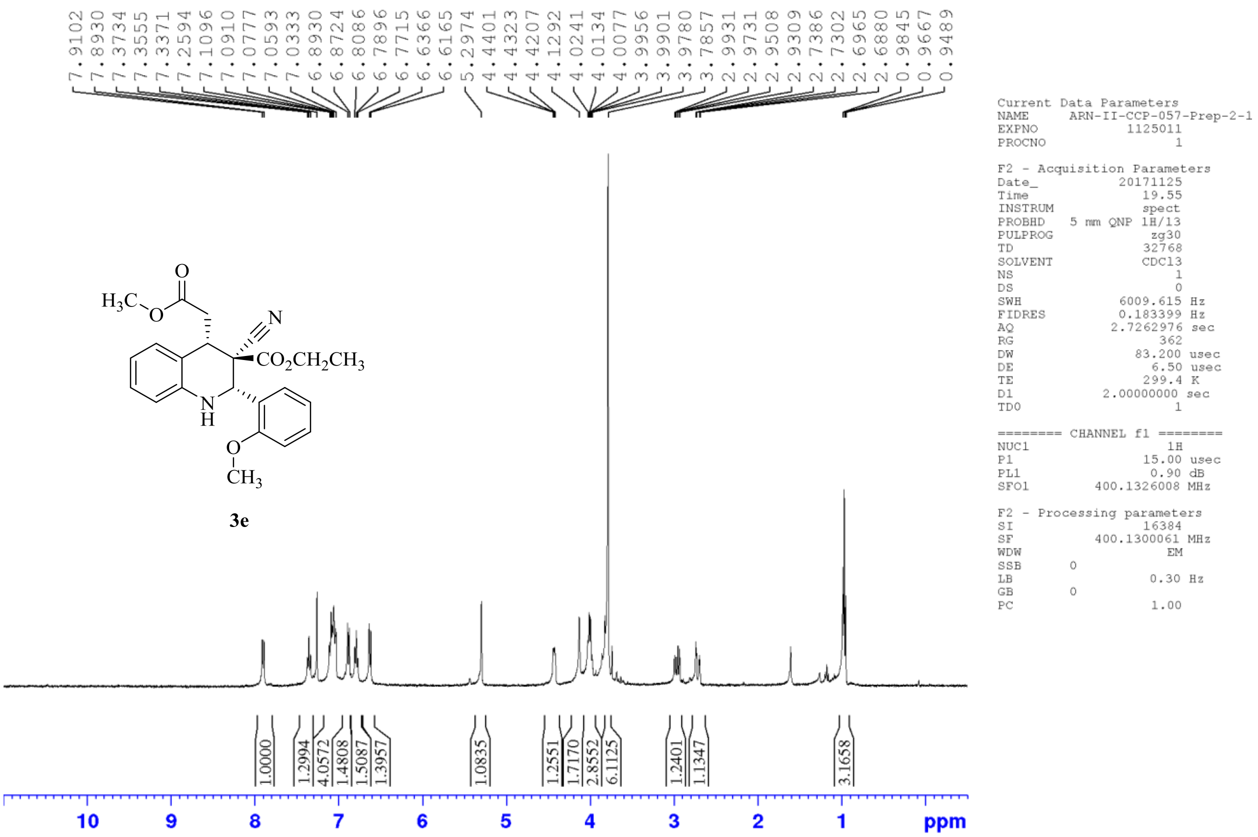

3e

Racemate of ethyl 3-cyano-4-(2-methoxy-2-oxoethyl)-2-(2-methoxyphenyl)-1,2,3,4-tetrahydroquinoline-3-carboxylate (**3e**)

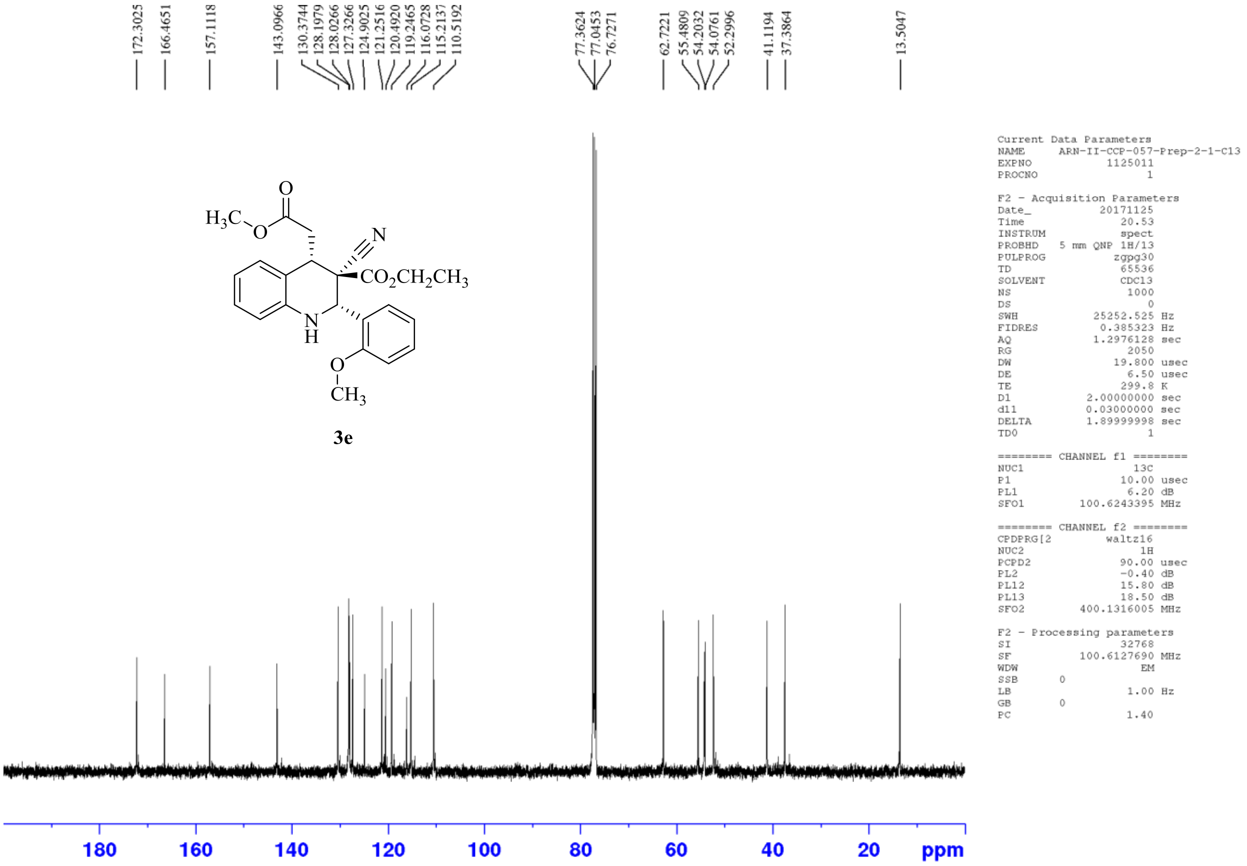

Racemate of 3-ethyl 4-methyl-3-cyano-2-(3,5-dimethoxyphenyl)-1,2,3,4-tetrahydroquinoline-3,4-dicarboxylate (**3f**)

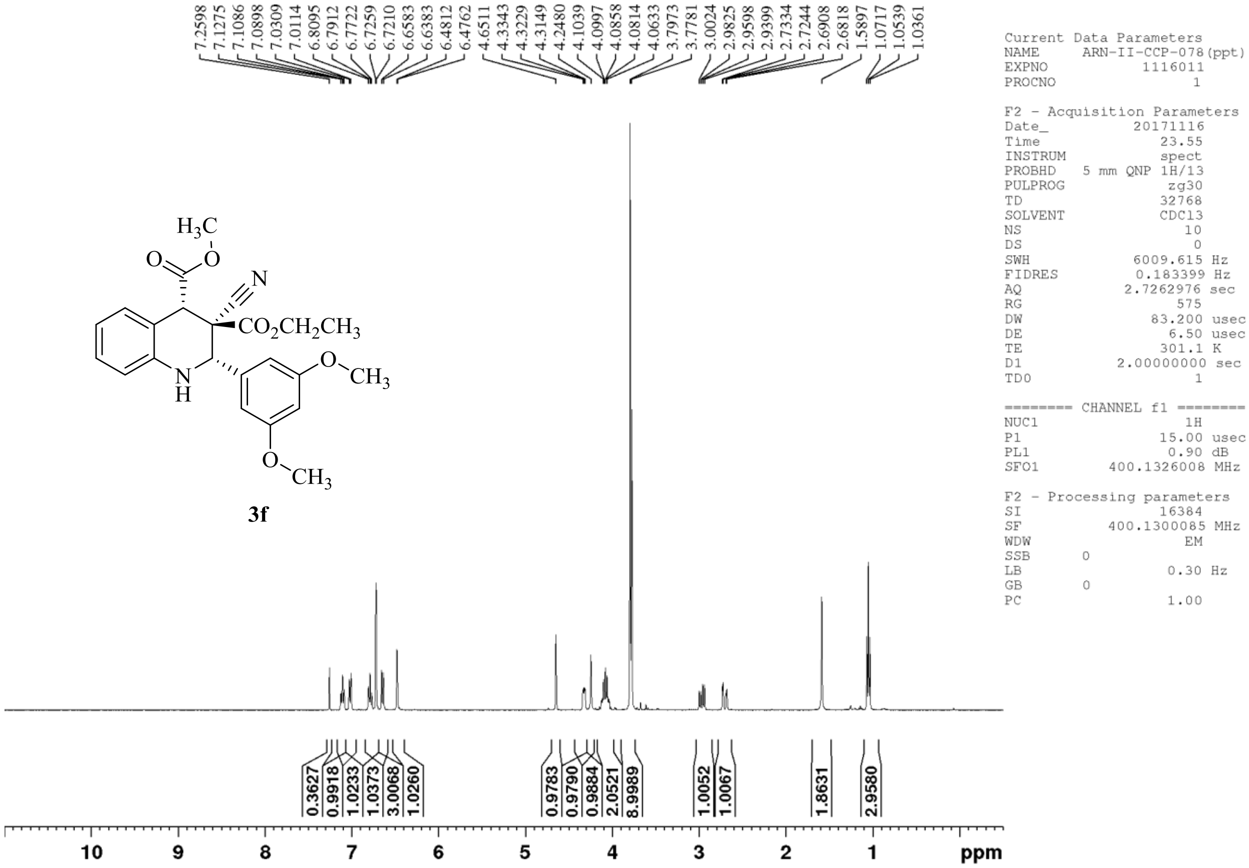

Racemate of 3-ethyl 4-methyl 3-cyano-2-(3,5-dimethoxyphenyl)-1,2,3,4-tetrahydroquinoline-3,4-dicarboxylate (**3f**)

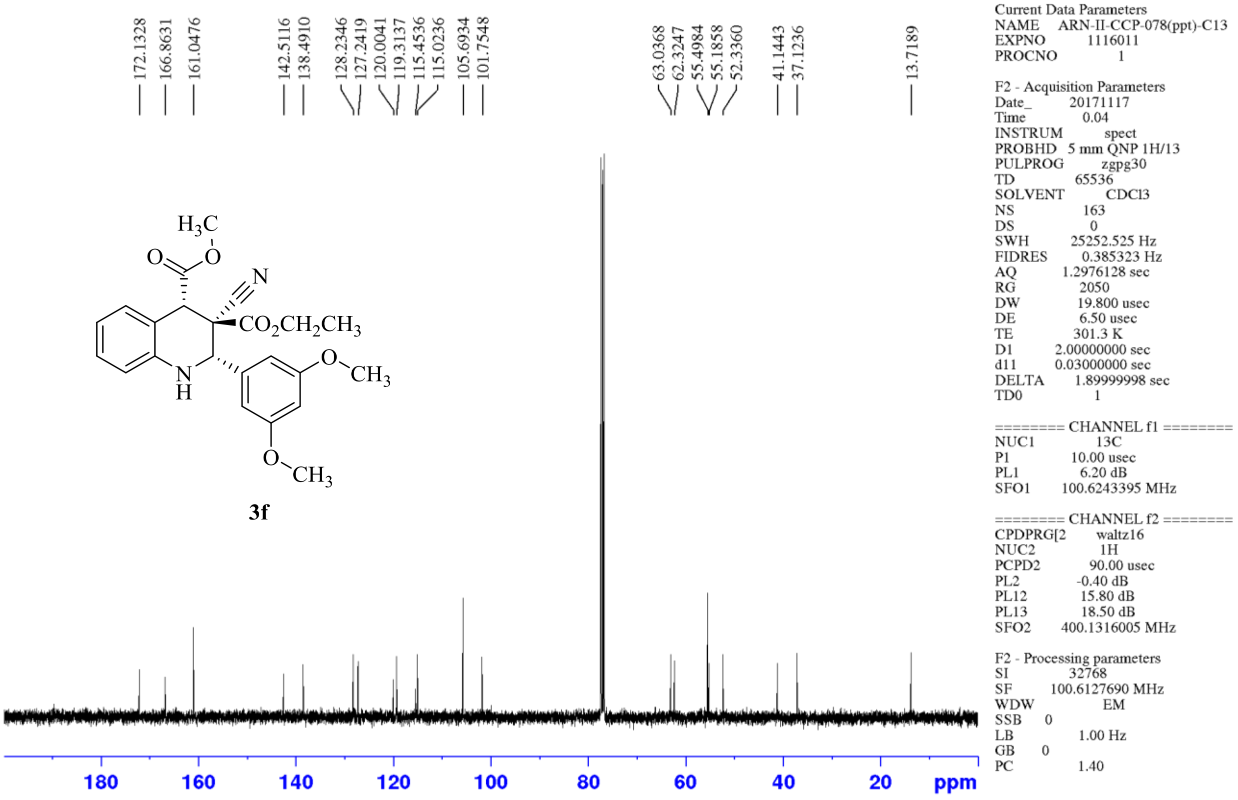

Racemate of ethyl 4-(2-(*tert*-butoxy)-2-oxoethyl)-3-cyano-2-(4-nitrophenyl)-1,2,3,4-tetrahydroquinoline-3-carboxylate (**4a**, minor isomer)

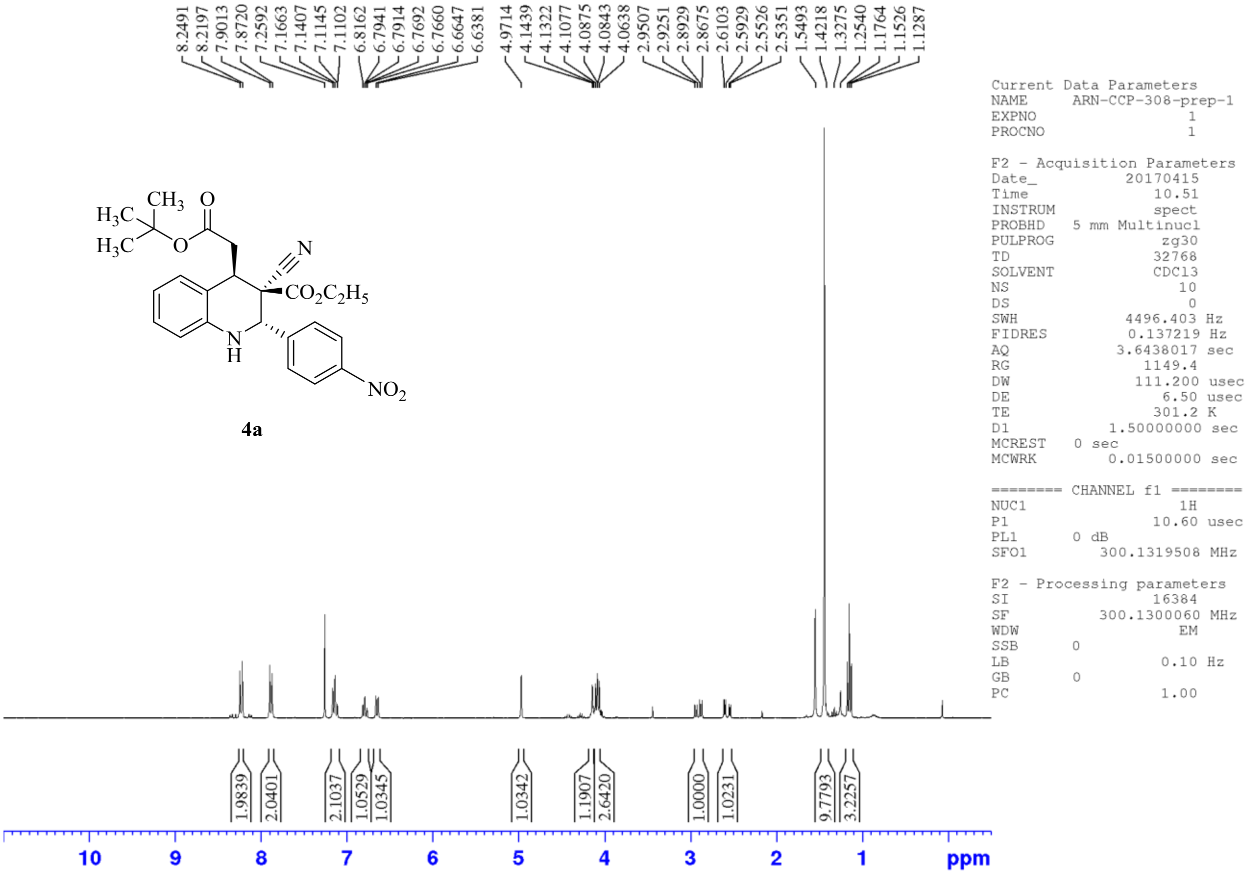

4a

Racemate of ethyl 4-(2-(*tert*-butoxy)-2-oxoethyl)-3-cyano-2-(4-nitrophenyl)-1,2,3,4-tetrahydroquinoline-3-carboxylate (**4a**, minor)

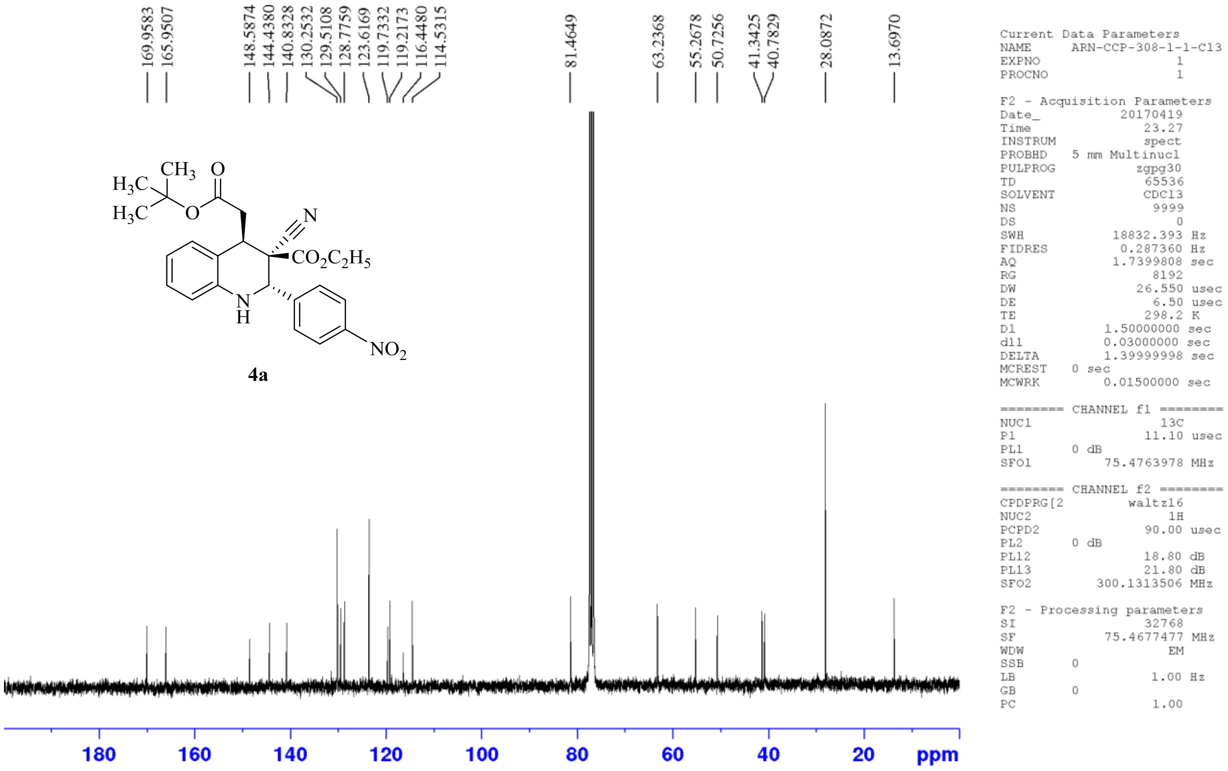

Racemate of ethyl 4-(2-(*tert*-butoxy)-2-oxoethyl)-3-cyano-2-(4-nitrophenyl)-1,2,3,4-tetrahydroquinoline-3-carboxylate (**4a**, minor)

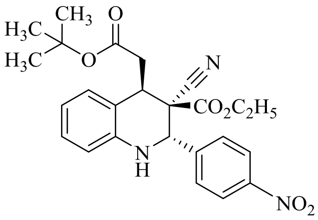

4a

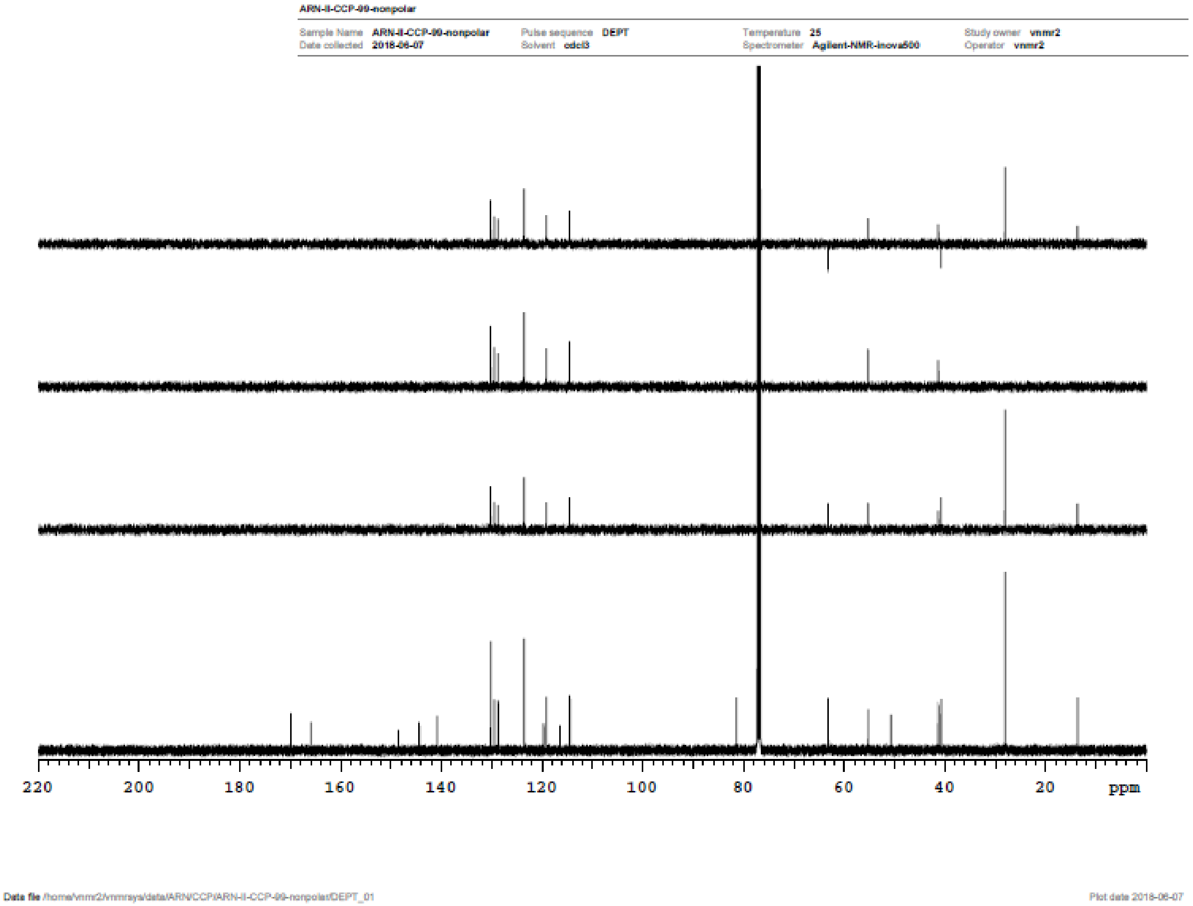

Ethyl 3-cyano-4-(2-methoxy-2-oxoethyl)-2-(4-nitrophenyl)-1,2,3,4-tetrahydroquinoline-3-carboxylate (**4d**, minor isomer)

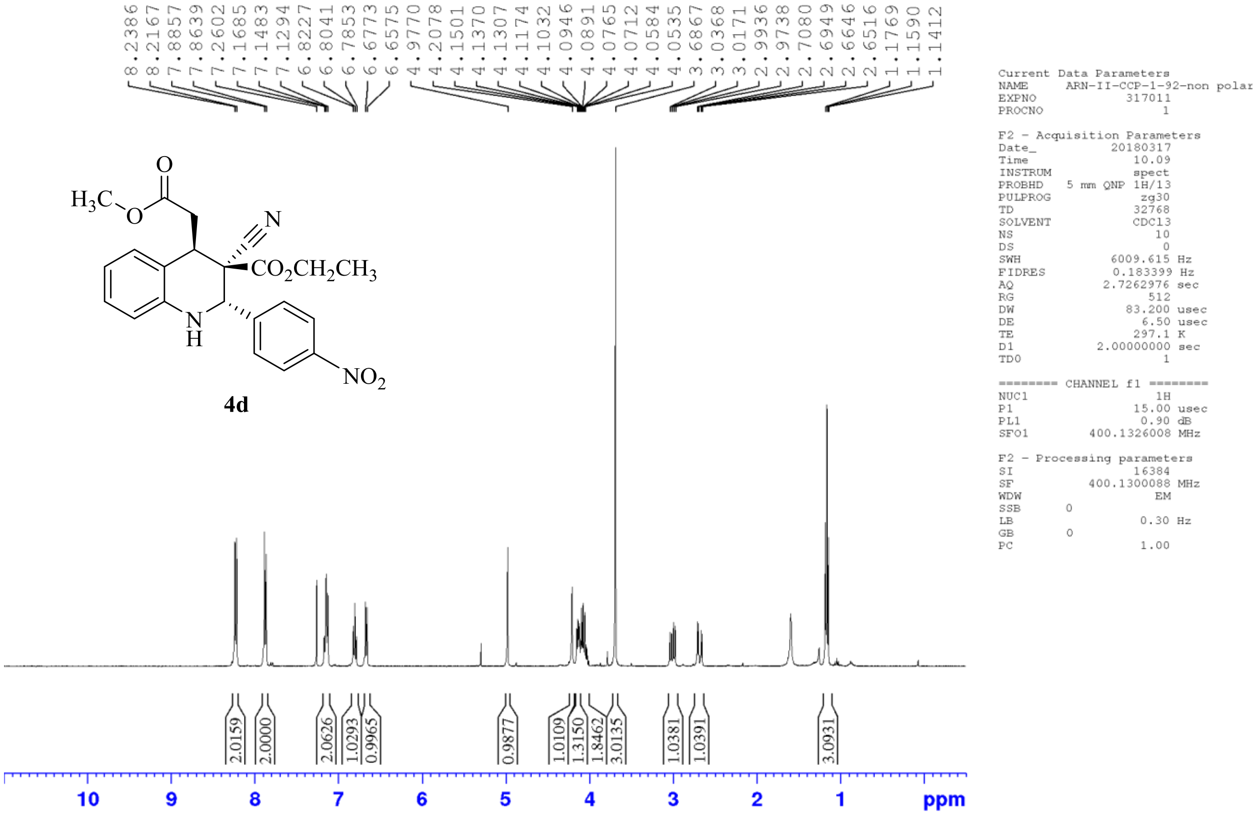

Ethyl 3-cyano-4-(2-methoxy-2-oxoethyl)-2-(4-nitrophenyl)-1,2,3,4-tetrahydroquinoline-3-carboxylate (**4d**, minor isomer)

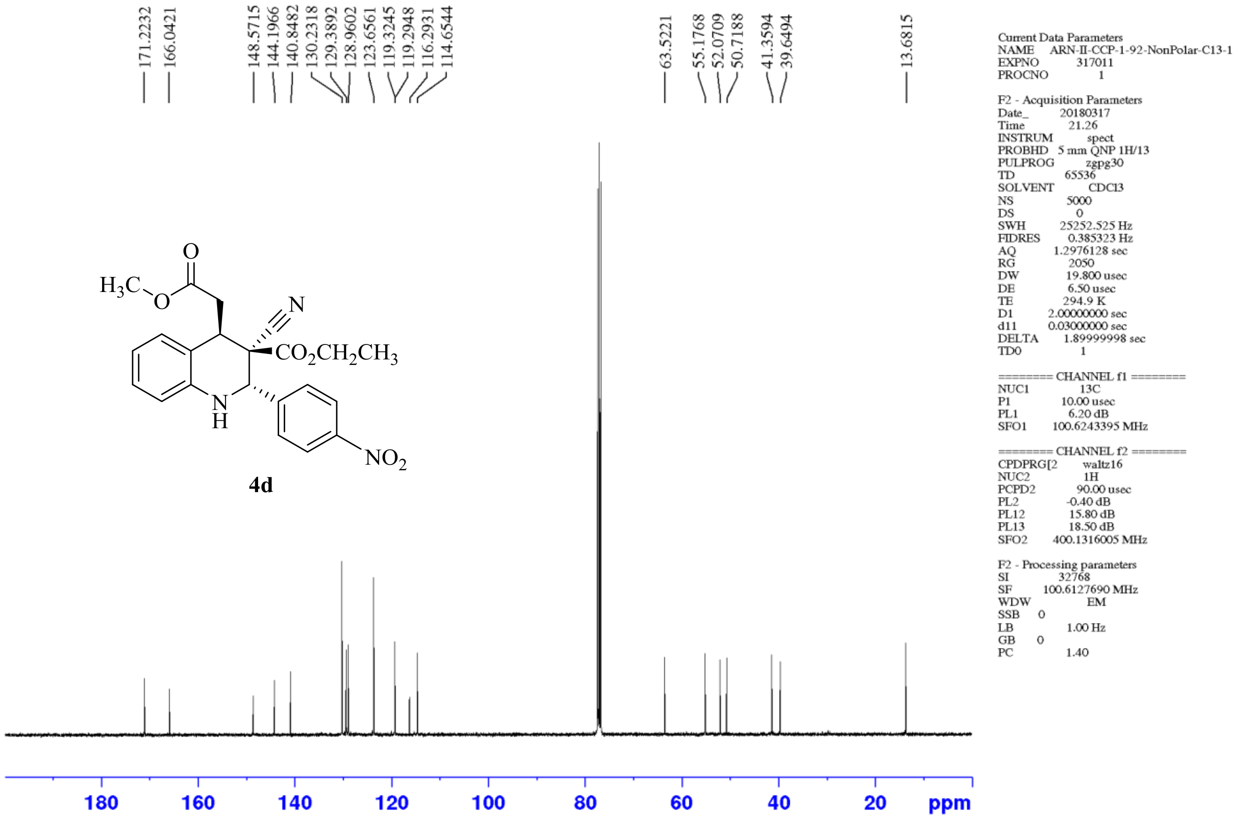

Racemate of ethyl 3-cyano-4-(2-methoxy-2-oxoethyl)-2-(2,4,6-trimethoxyphenyl)-1,2,3,4-tetrahydroquinoline-3-carboxylate (**5a**)

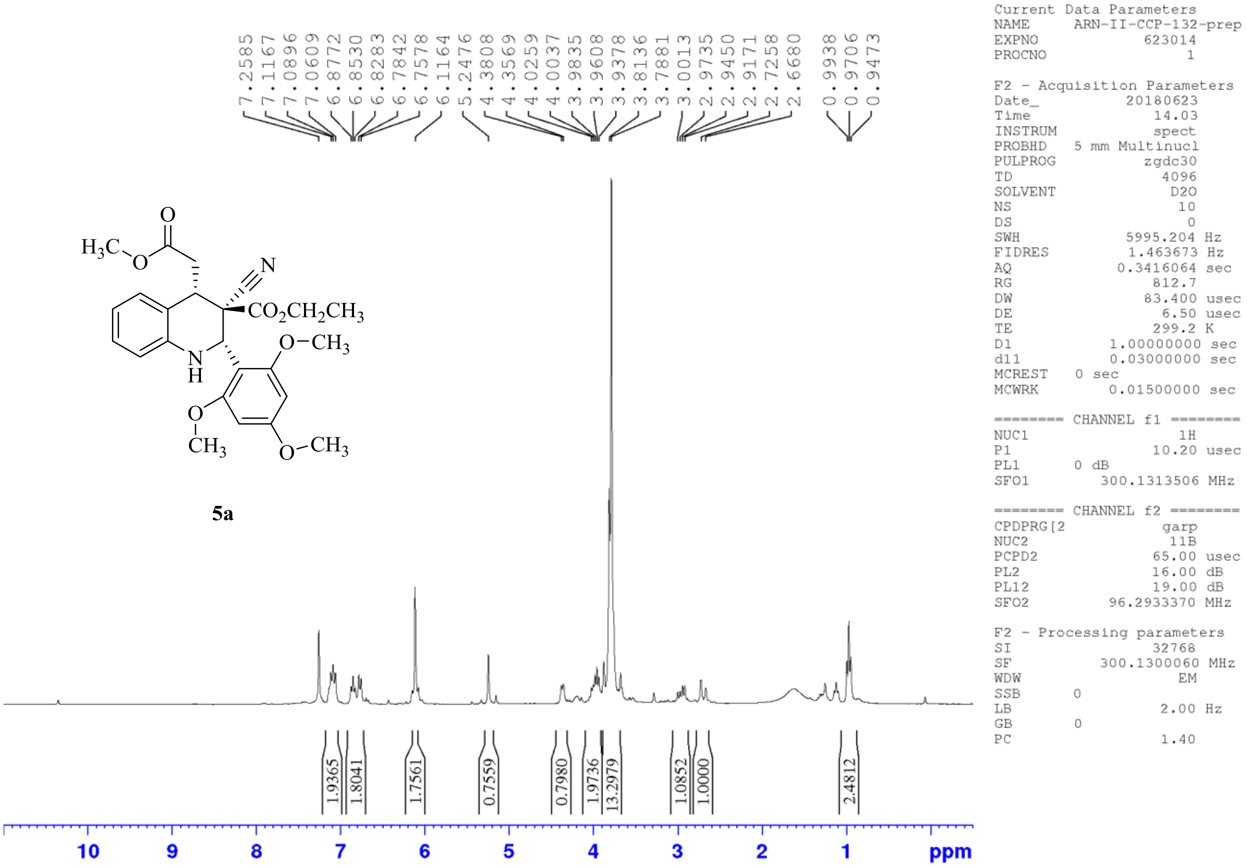

Racemate of methyl 2-((3-cyano-3-((ethylperoxy)-l2-methyl)-2-(2,4,6-trimethoxyphenyl)-1,2,3,4-tetrahydroquinolin-4-yl)acetate (**5a**)

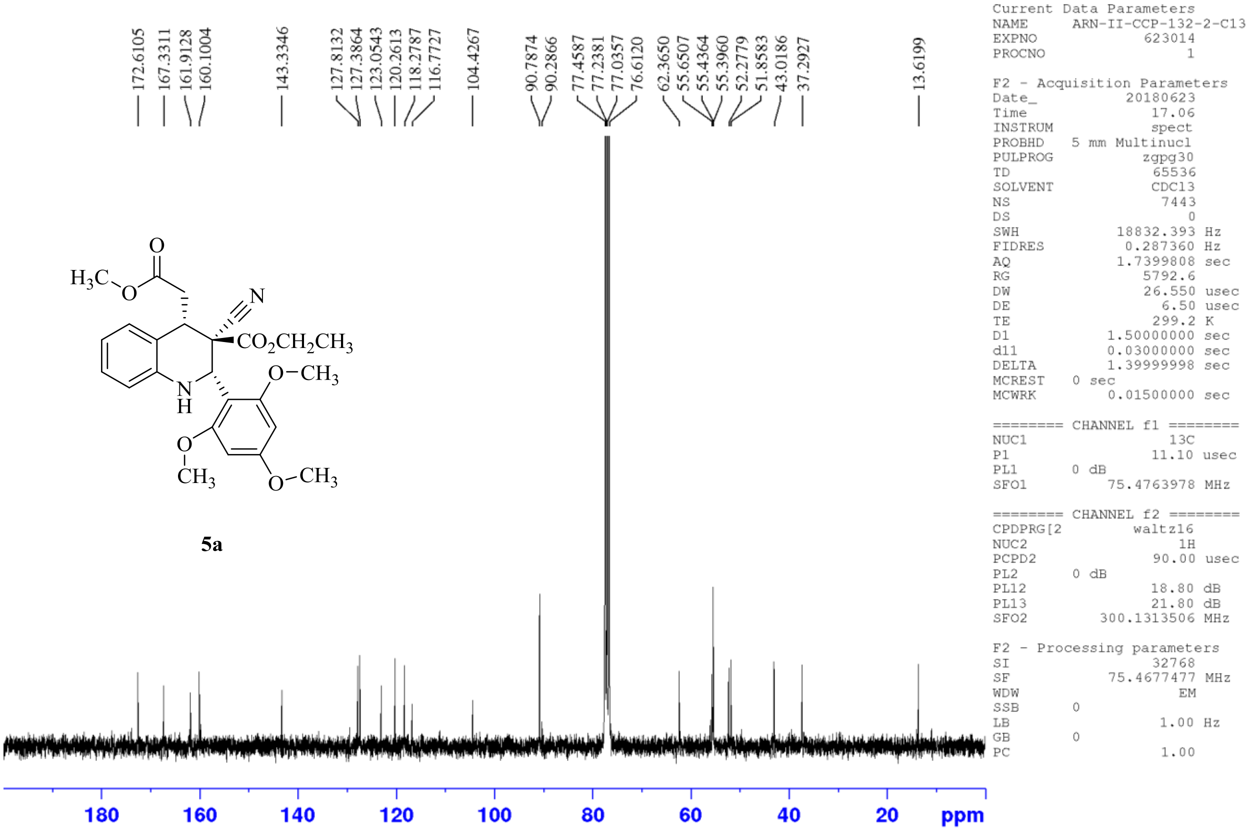

Ethyl (*Z*)-2-cyano-3-(2,4,6-trimethoxyphenyl)acrylate (**5a1**)

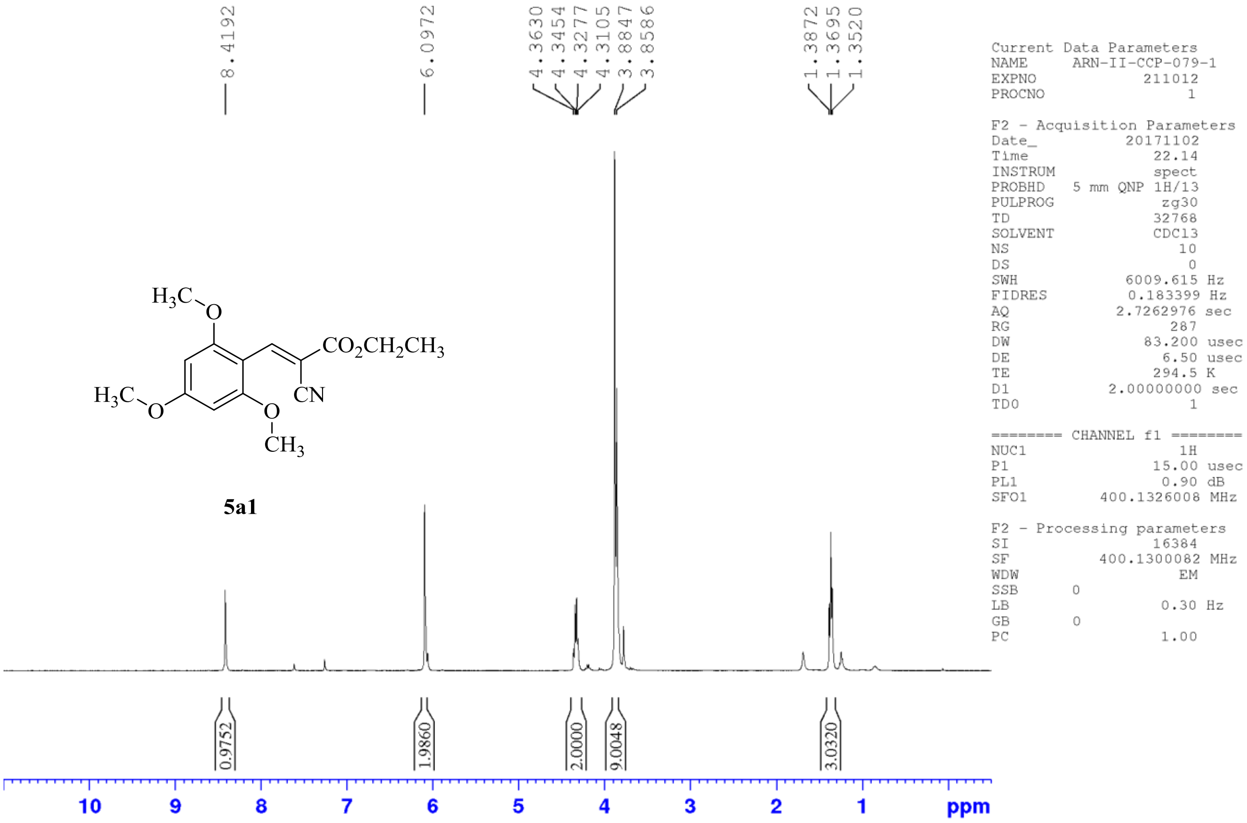

Ethyl (*Z*)-2-cyano-3-(2,4,6-trimethoxyphenyl)acrylate (**5a1**)

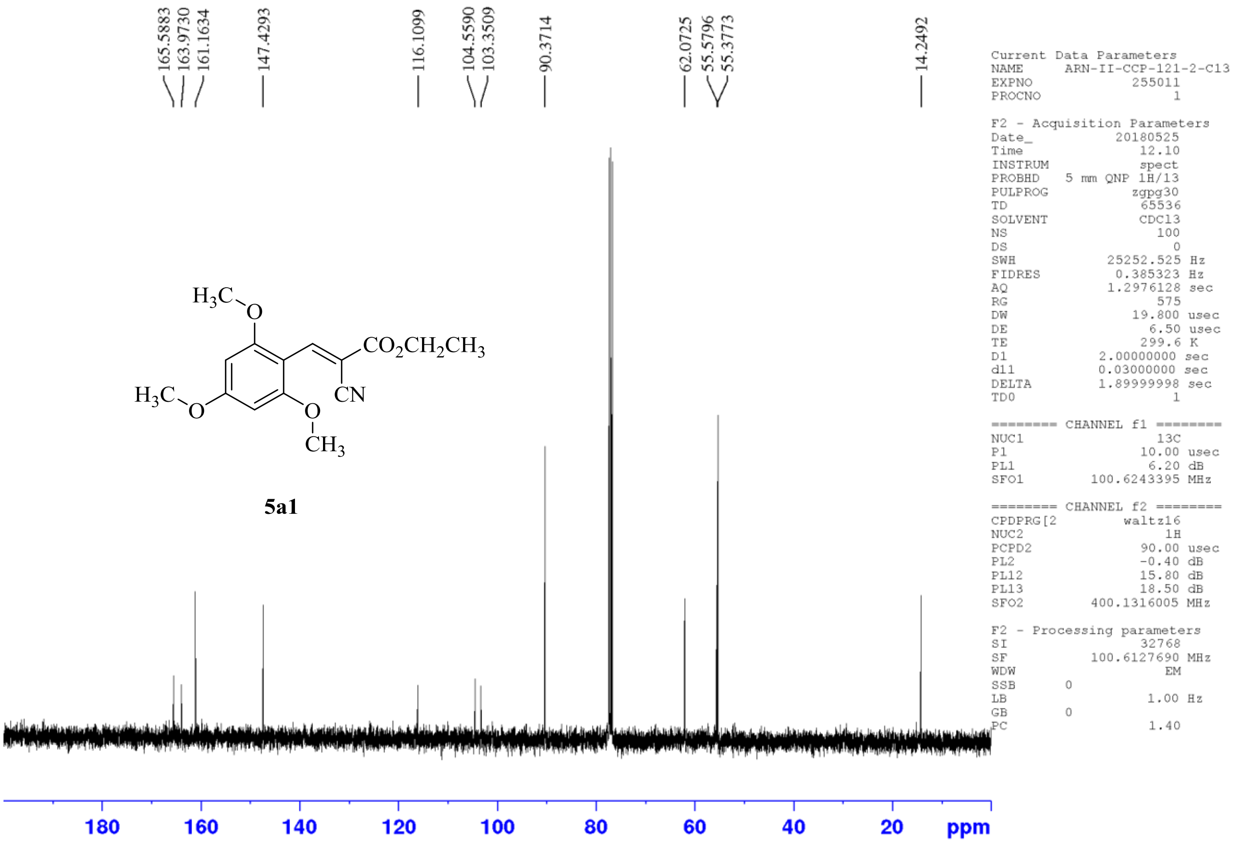

5a1

Racemate ofethyl 3-cyano-4-(2-methoxy-2-oxoethyl)-2-(naphthalen-1-yl)-1,2,3,4-tetrahydroquinoline-3-carboxylate (**5b**)

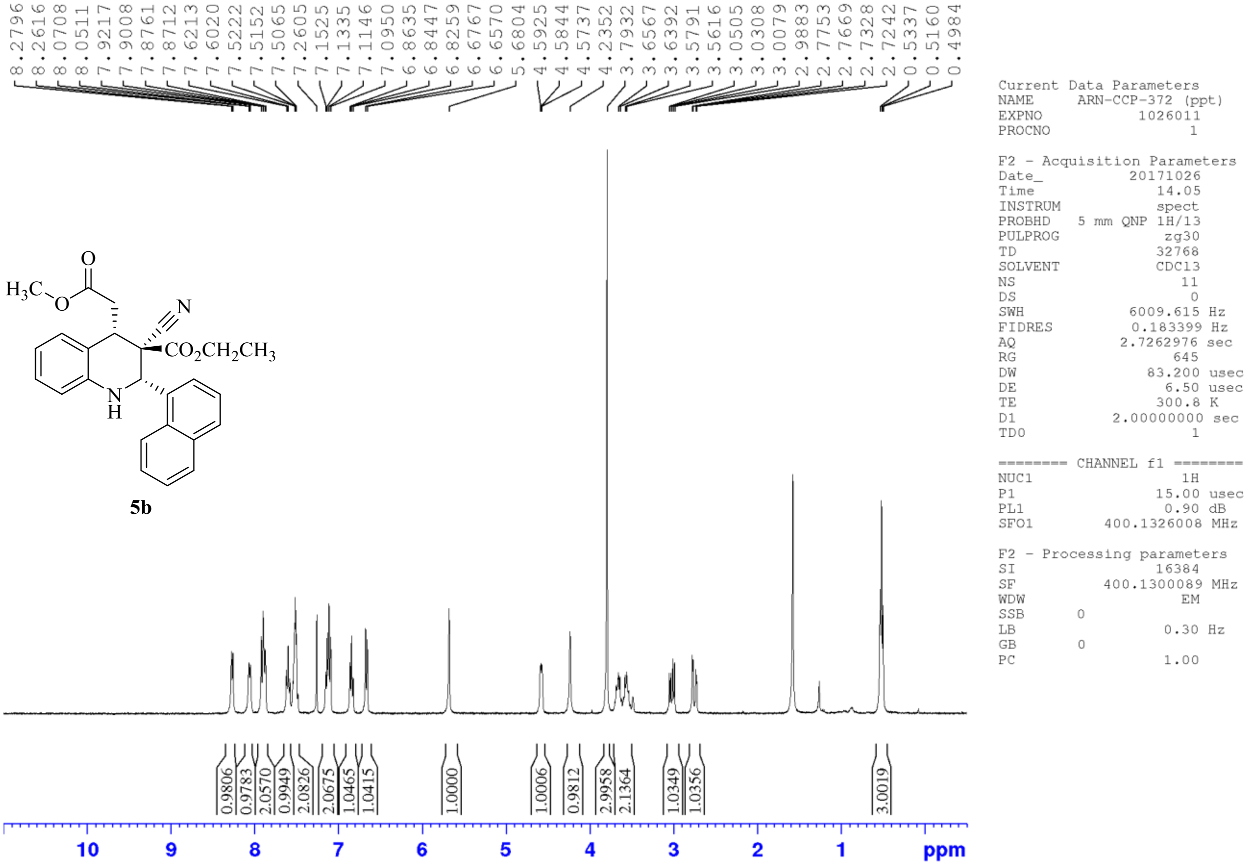

Racemate of ethyl 3-cyano-4-(2-methoxy-2-oxoethyl)-2-(naphthalen-1-yl)-1,2,3,4-tetrahydroquinoline-3-carboxylate (**5b**)

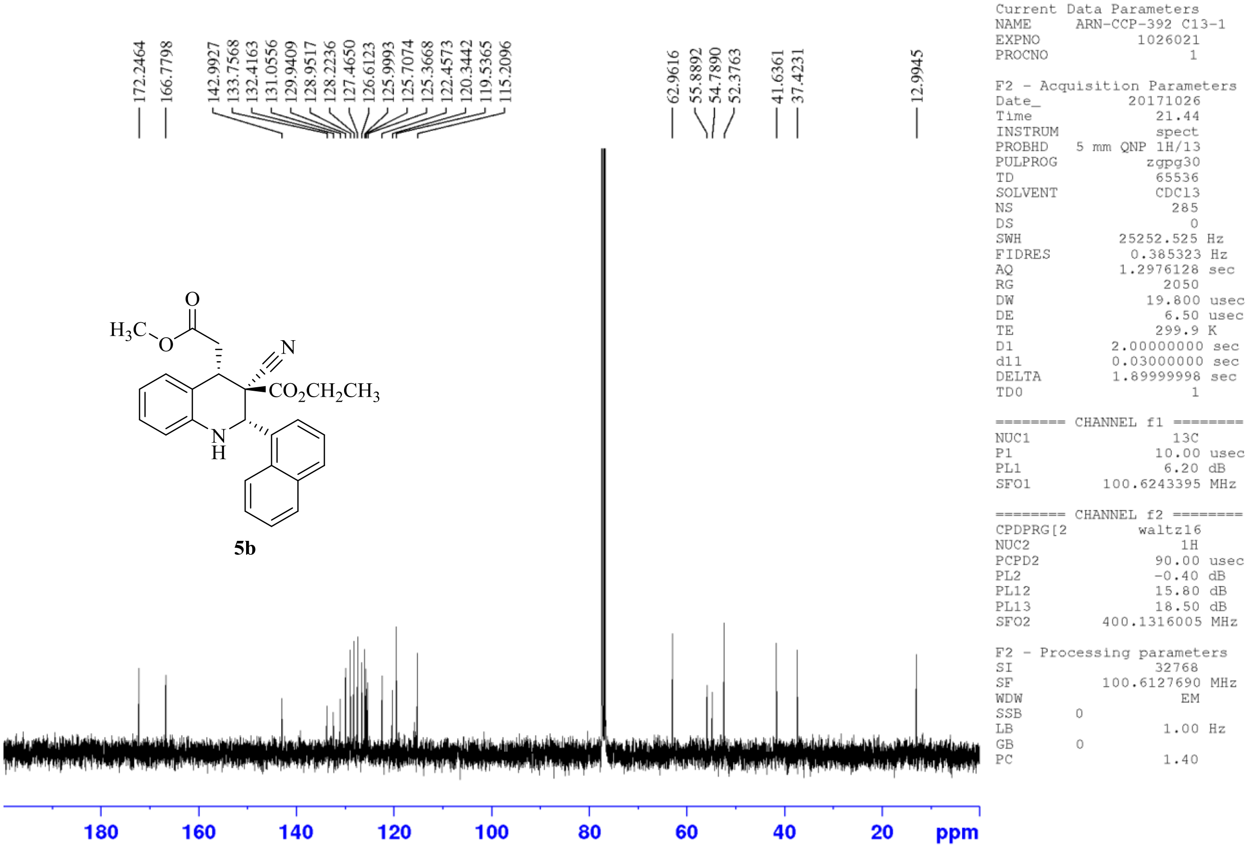

Racemate of ethyl 3-cyano-4-(2-methoxy-2-oxoethyl)-2-(naphthalen-2-yl)-1,2,3,4-tetrahydroquinoline-3-carboxylate (**5c**)

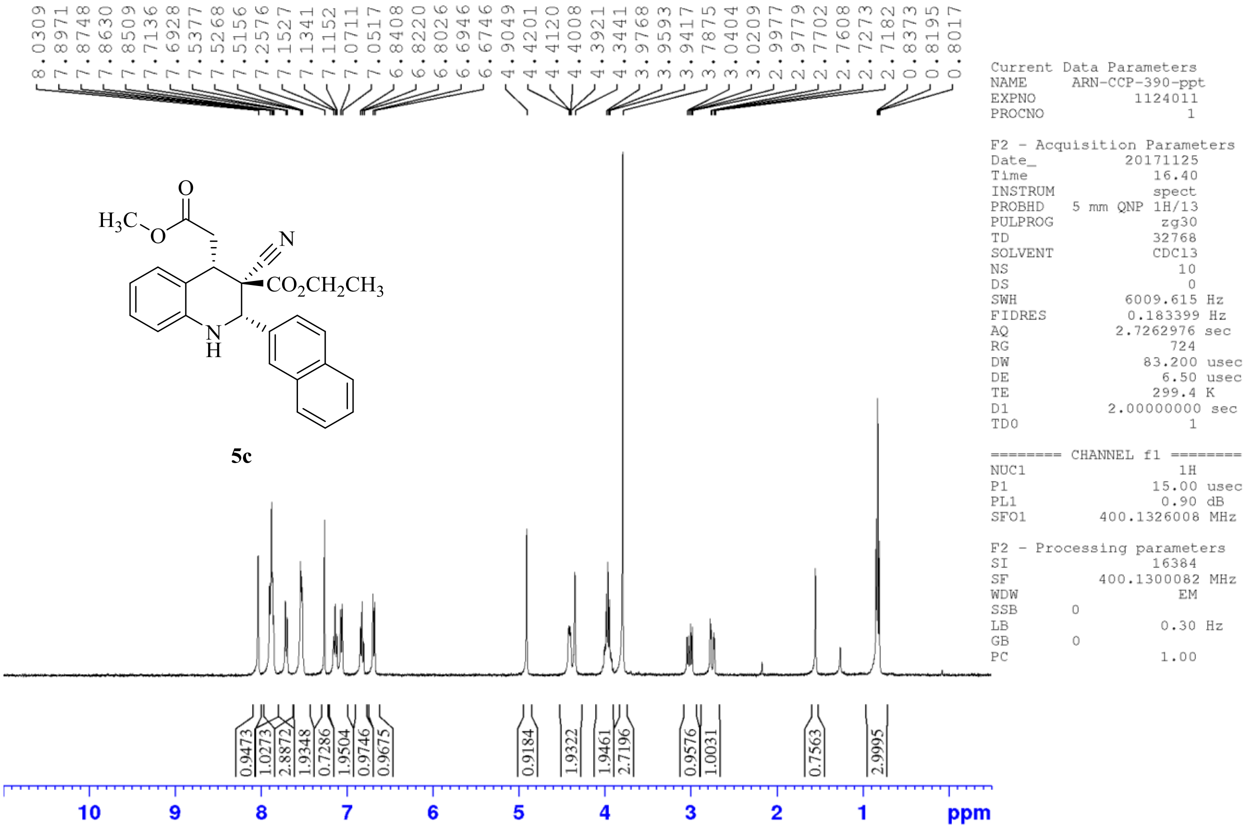

Racemate of ethyl 3-cyano-4-(2-methoxy-2-oxoethyl)-2-(naphthalen-2-yl)-1,2,3,4-tetrahydroquinoline-3-carboxylate (**5c**)

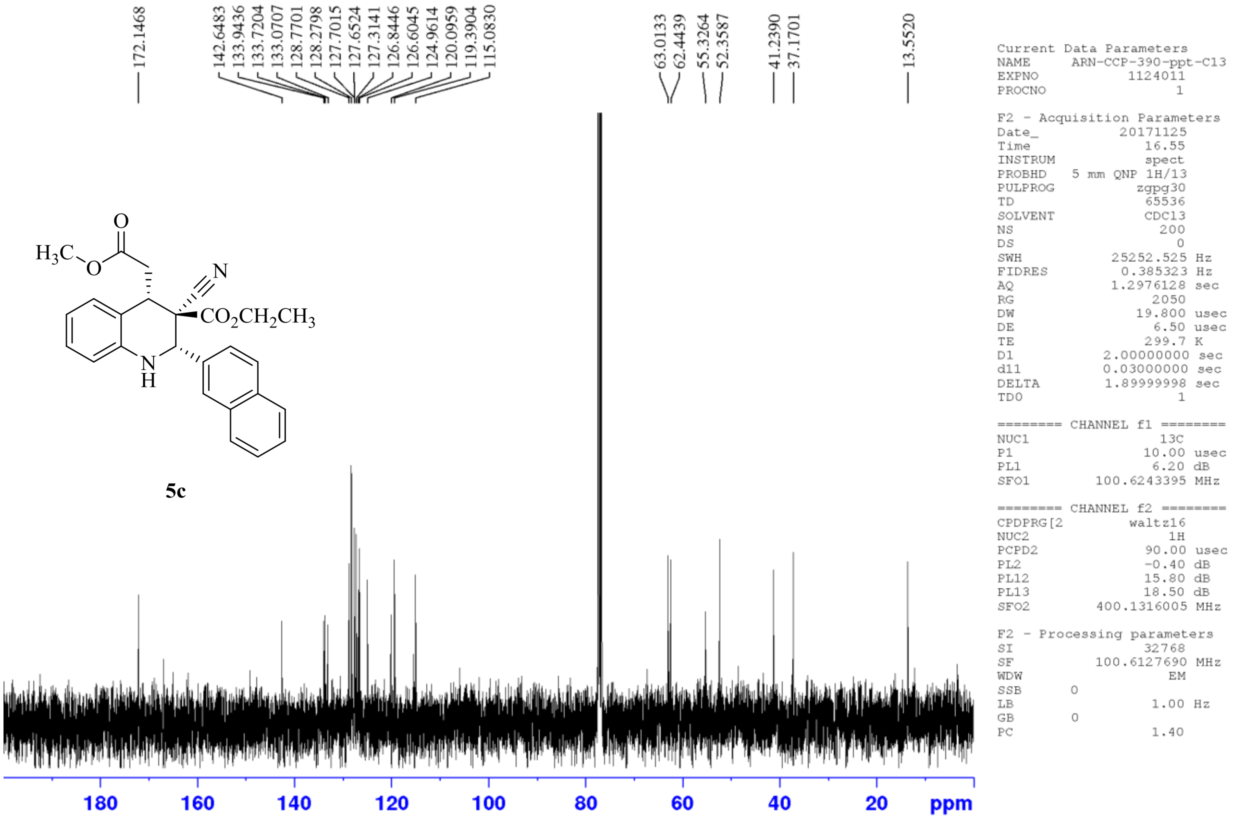

Racemate of ethyl 3-cyano-4-(2-methoxy-2-oxoethyl)-2-(4-methoxyphenyl)-1,2,3,4-tetrahydroquinoline-3-carboxylate (**5d**)

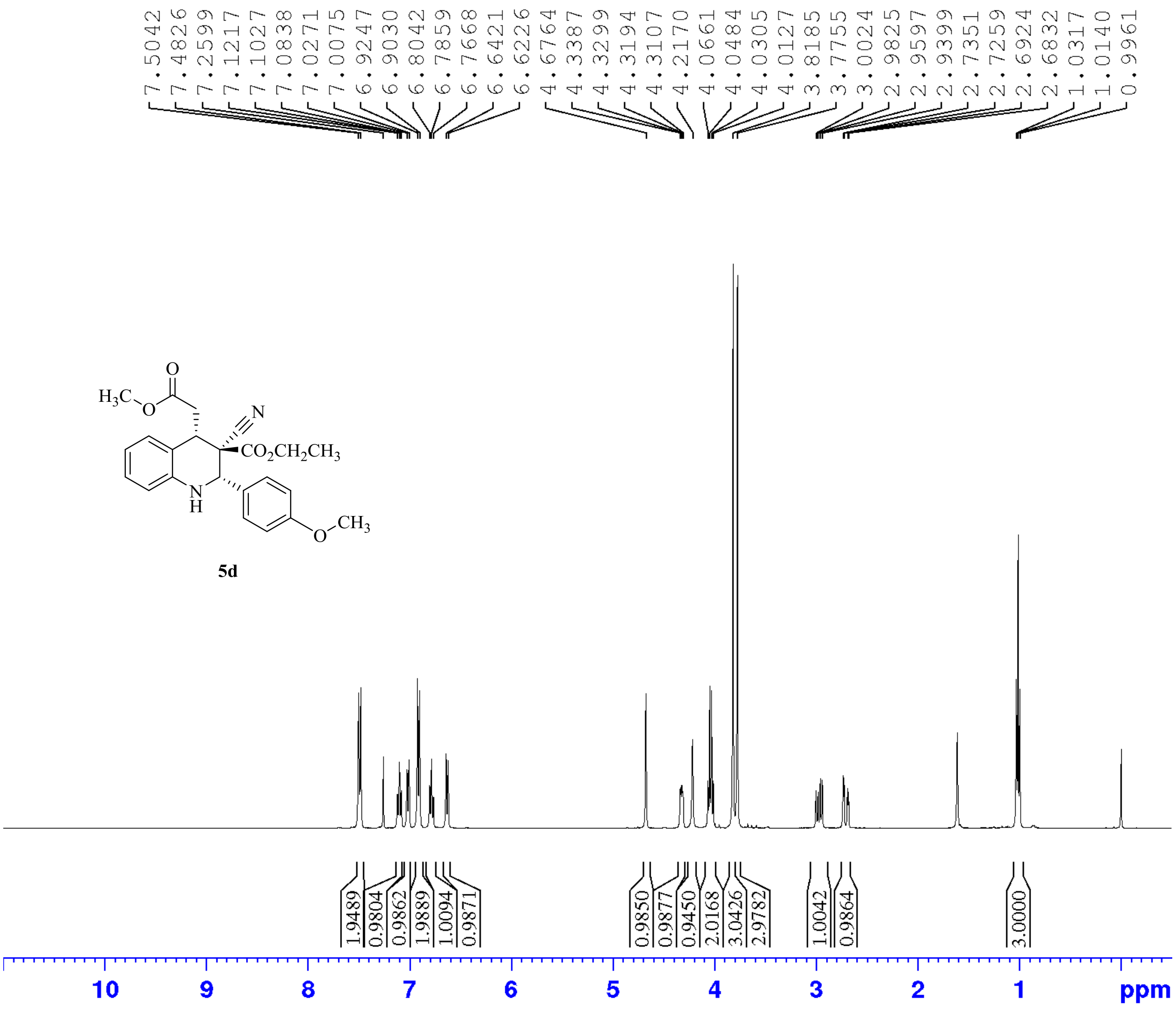

Racemate of ethyl 3-cyano-4-(2-methoxy-2-oxoethyl)-2-(4-methoxyphenyl)-1,2,3,4-tetrahydroquinoline-3-carboxylate (**5d**)

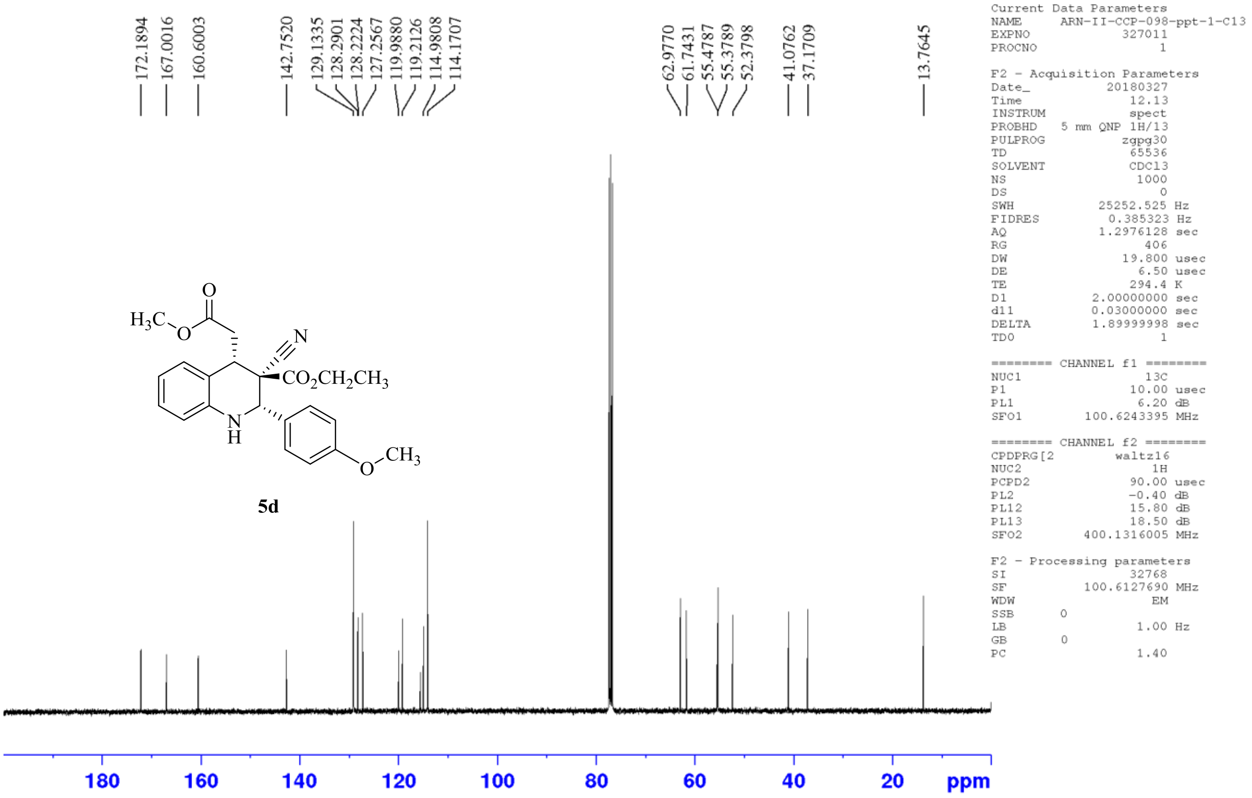

Ethyl (*Z*)-2-cyano-3-(4-nitrophenyl)acrylate (**7a**)

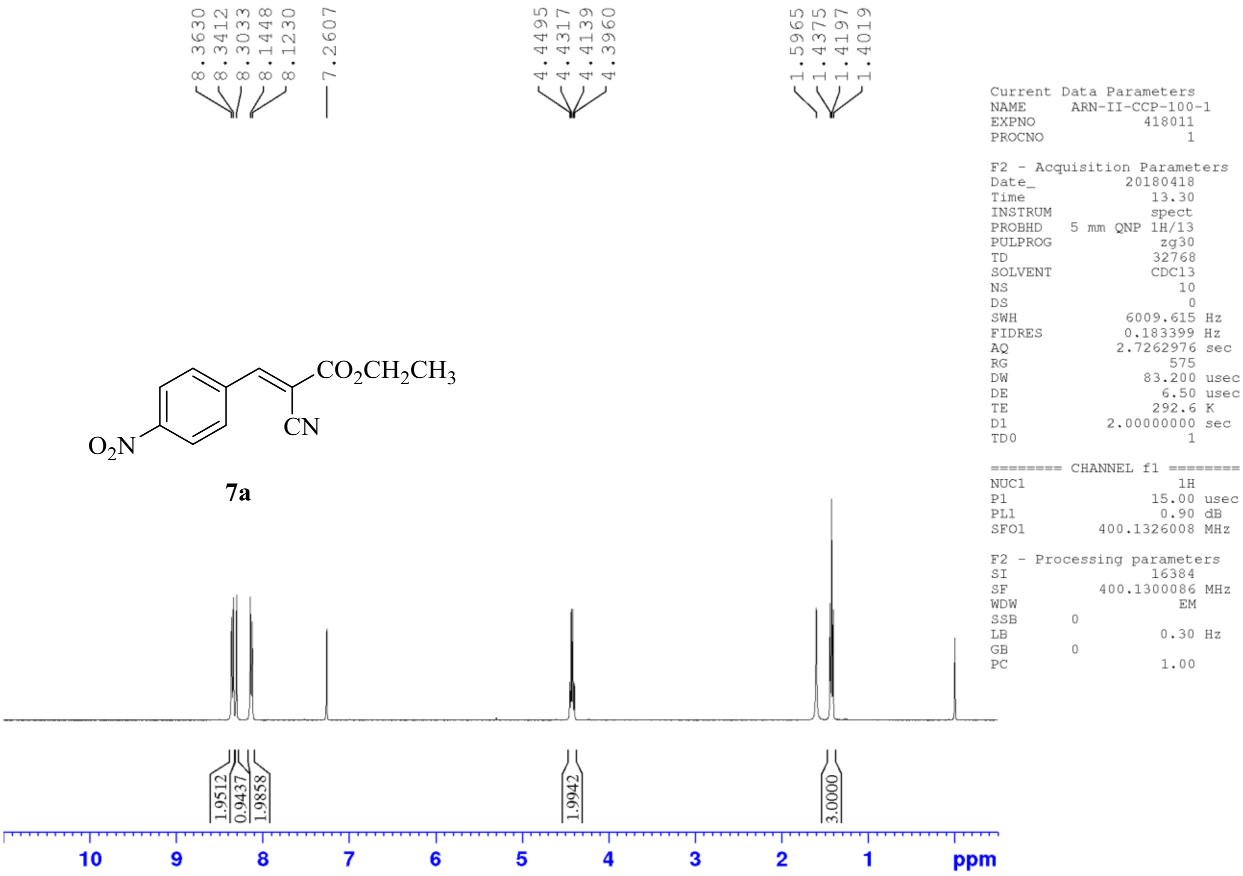

Ethyl (*Z*)-2-cyano-3-(4-nitrophenyl)acrylate (**7a**)

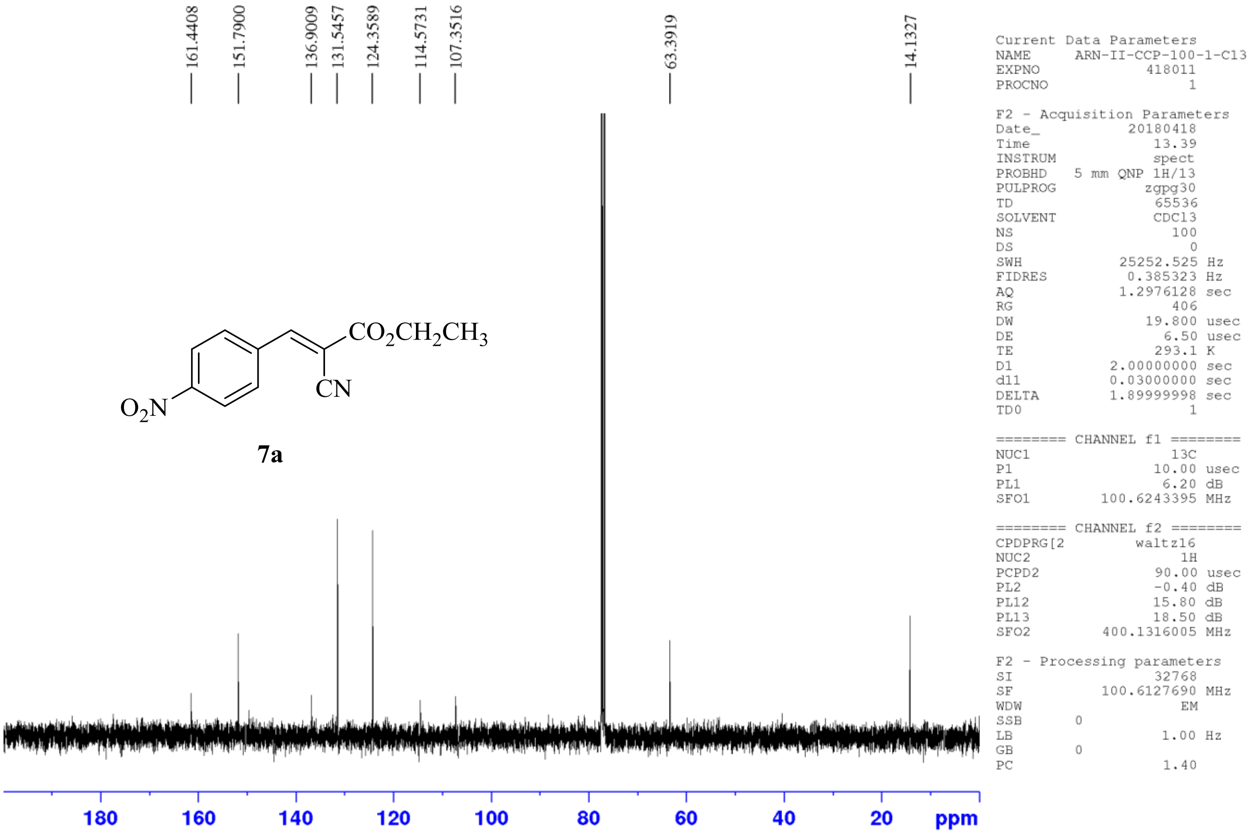

Ethyl (*E*)-2-cyano-3-phenylacrylate (**7b**)

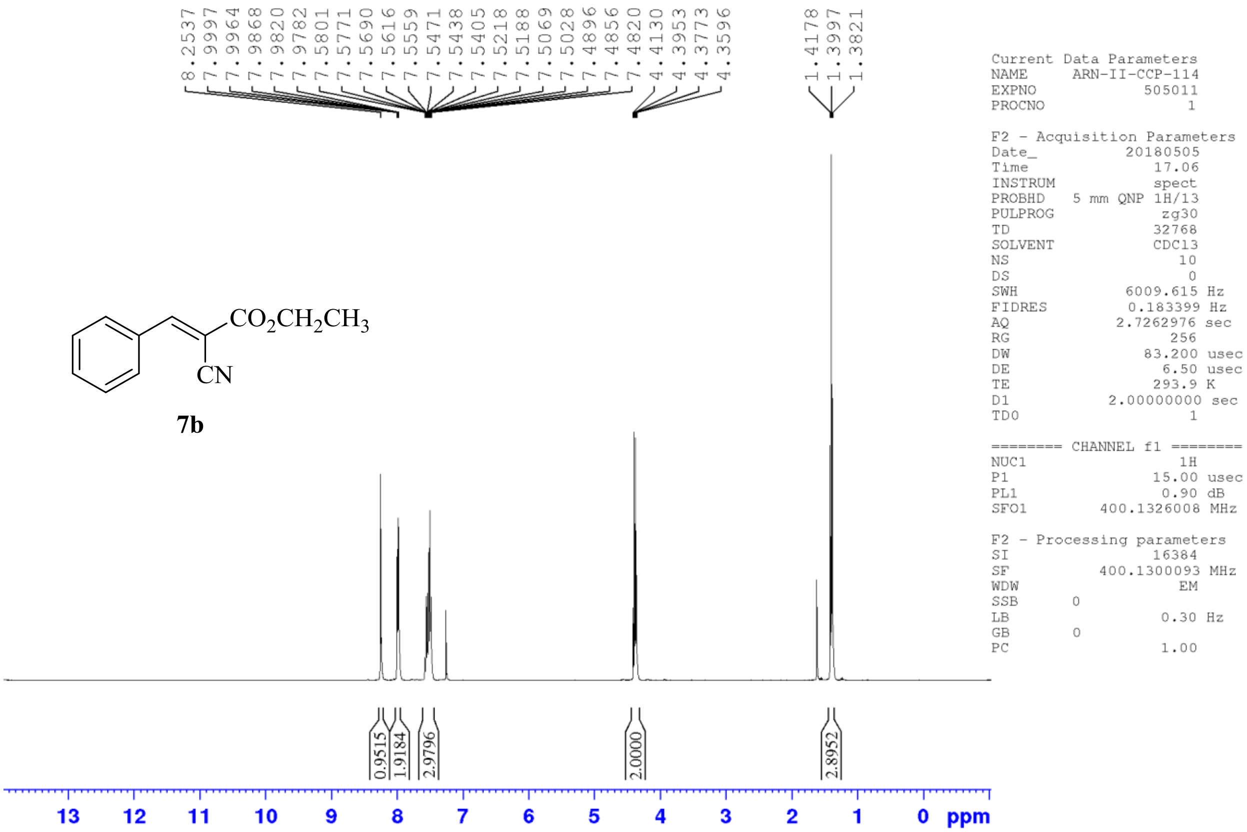

Ethyl (*E*)-2-cyano-3-phenylacrylate (**7b**)

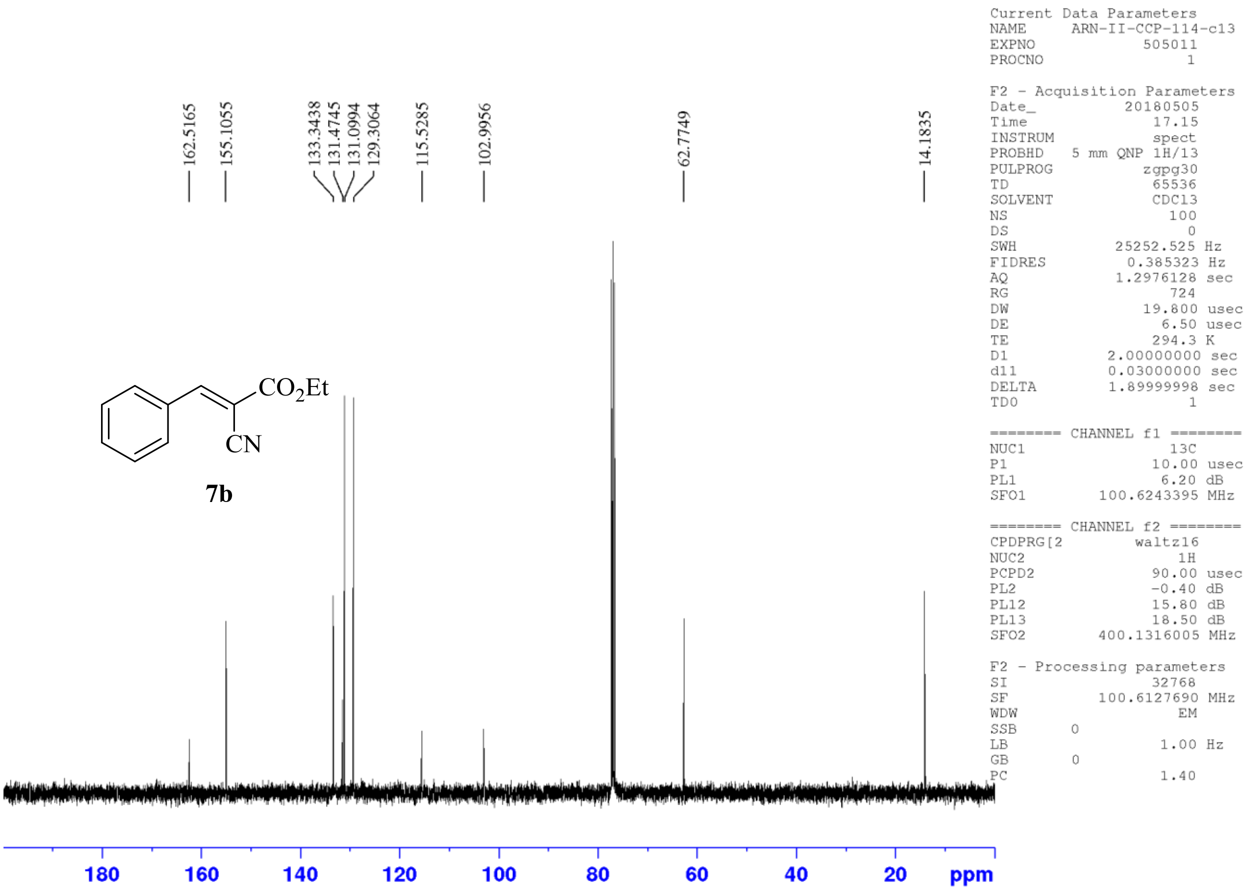

Ethyl (*Z*)-2-cyano-3-(4-methoxyphenyl) acrylate (**7c**)

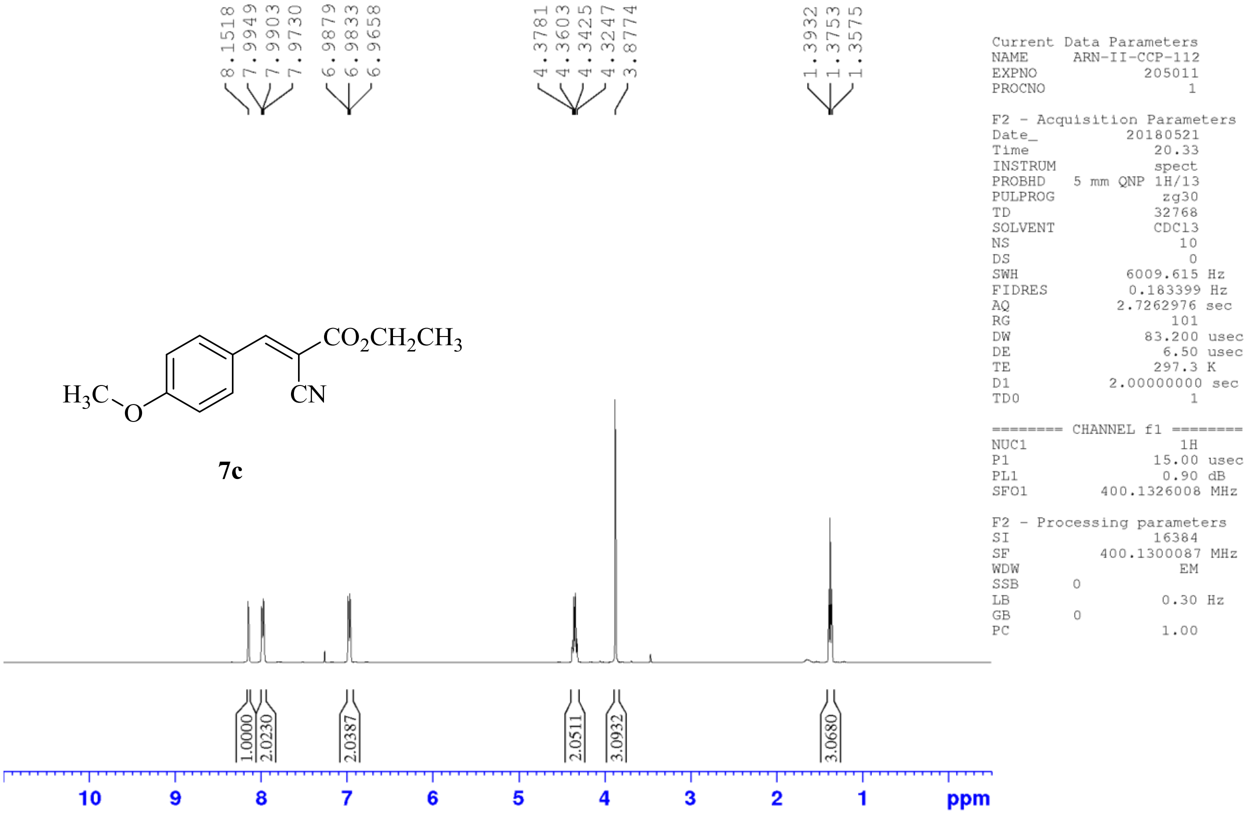

Ethyl (*Z*)-2-cyano-3-(4-methoxyphenyl) acrylate (**7c**)

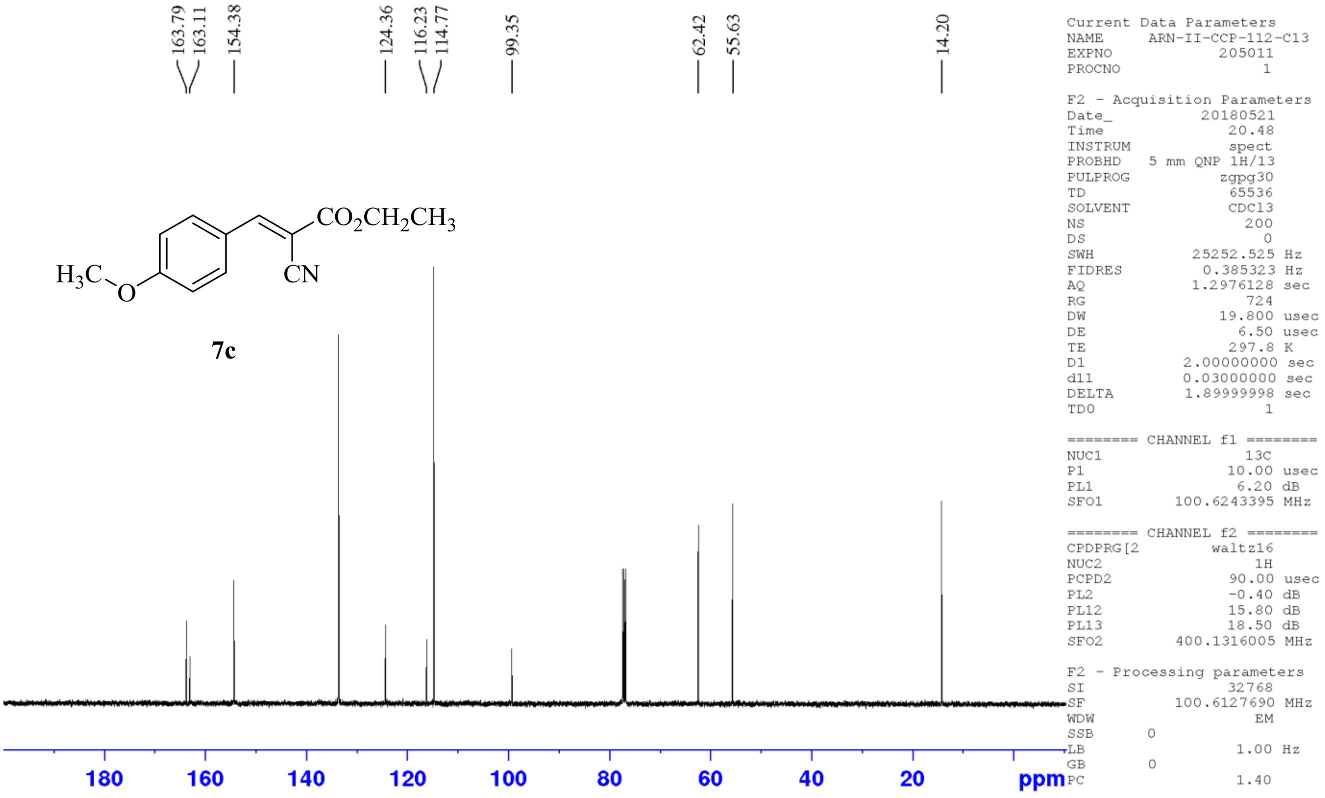

Ethyl (Z)-2-cyano-3-(3,5-dimethoxyphenyl)acrylate (**7d**)

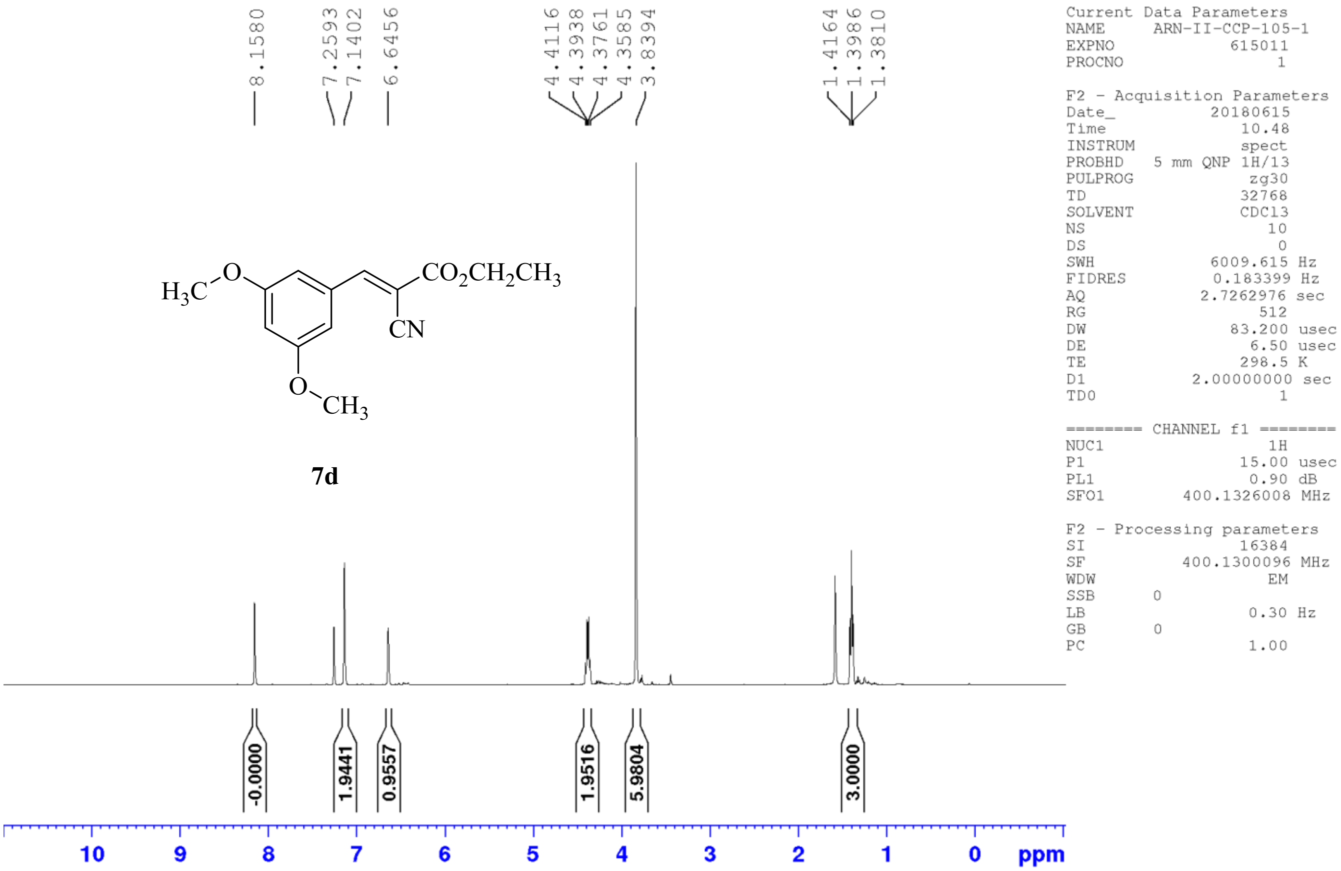

Ethyl (*Z*)-2-cyano-3-(3,5-dimethoxyphenyl)acrylate (**7d**)

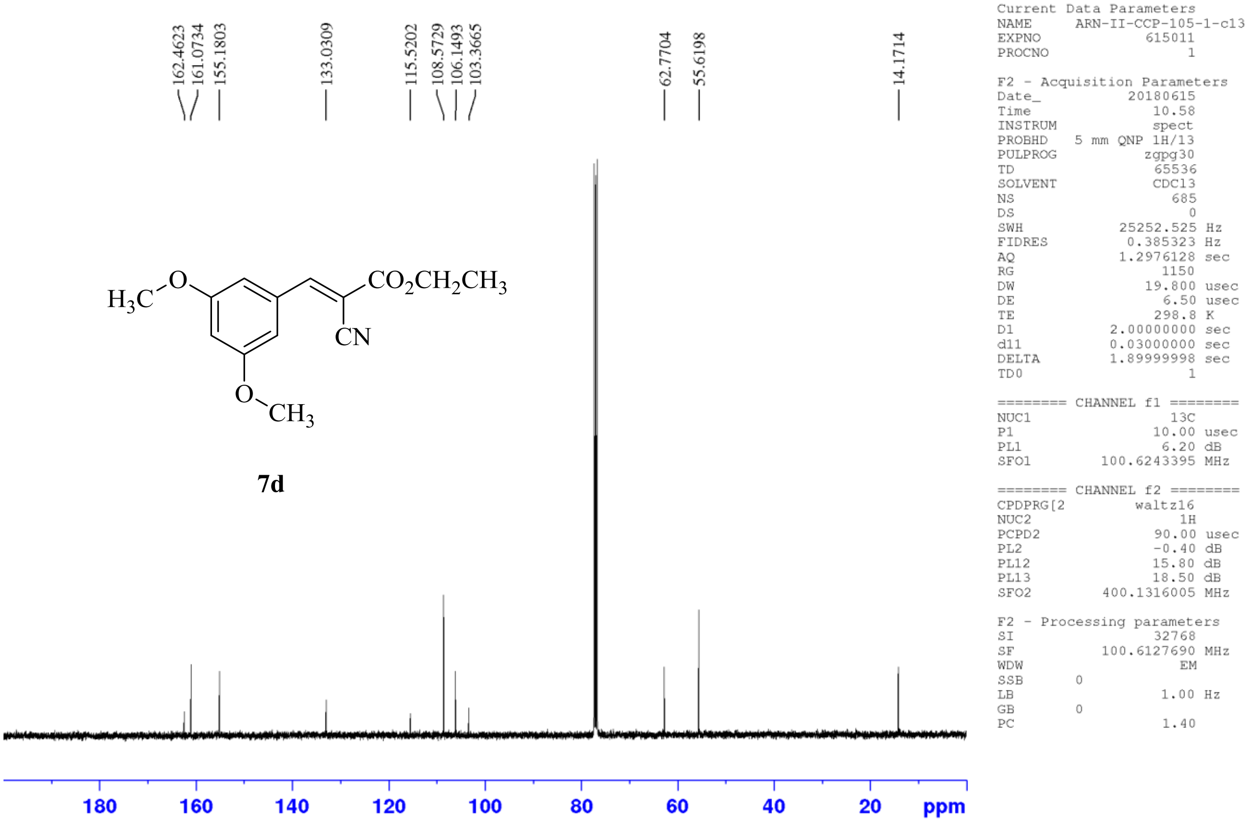

Racemate of ethyl 6-chloro-3-cyano-4-(2-methoxy-2-oxoethyl)-2-(2-methoxyphenyl)-1,2,3,4-tetrahydroquinoline-3-carboxylate (**9a**)

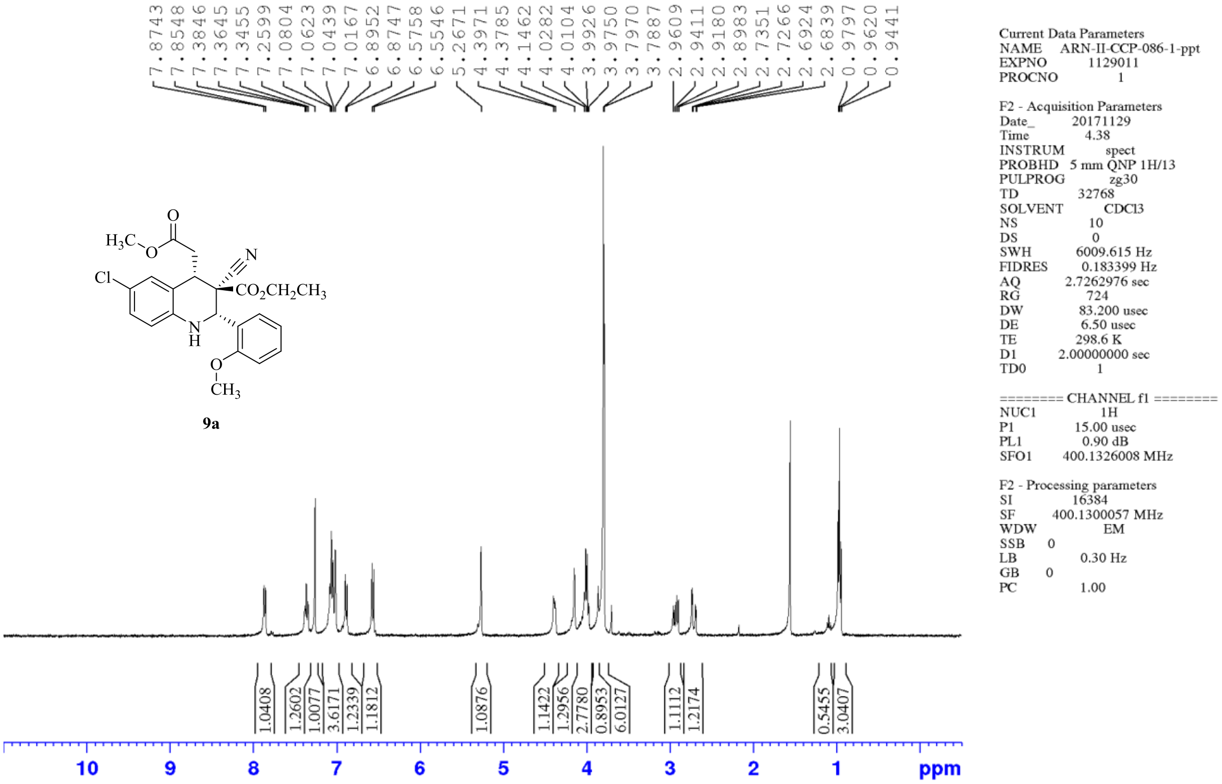

Racemate of ethyl 6-chloro-3-cyano-4-(2-methoxy-2-oxoethyl)-2-(2-methoxyphenyl)-1,2,3,4-tetrahydroquinoline-3-carboxylate (**9a**)

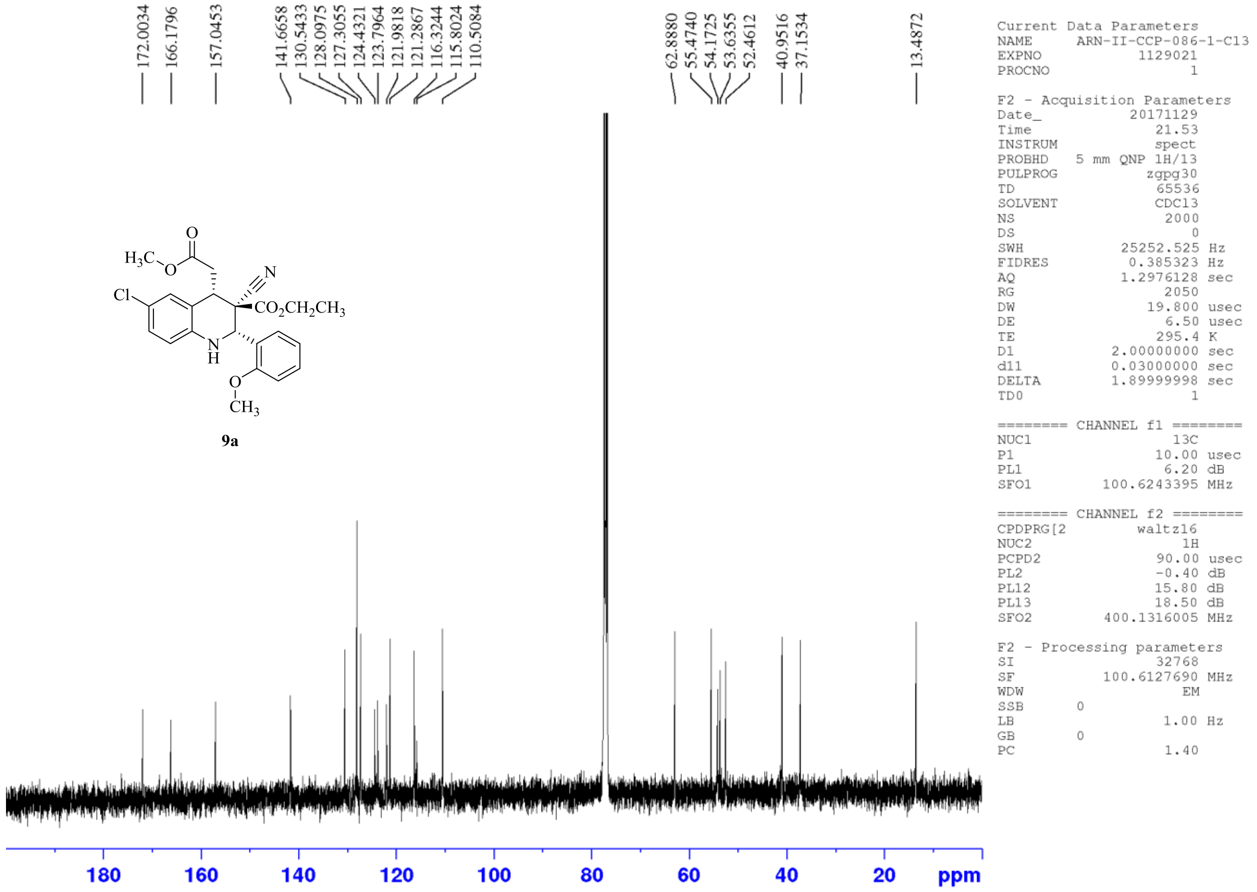

Racemate of ethyl 6-chloro-3-cyano-4-(2-methoxy-2-oxoethyl)-2-(2-methoxyphenyl)-1,2,3,4-tetrahydroquinoline-3-carboxylate (**9b**)

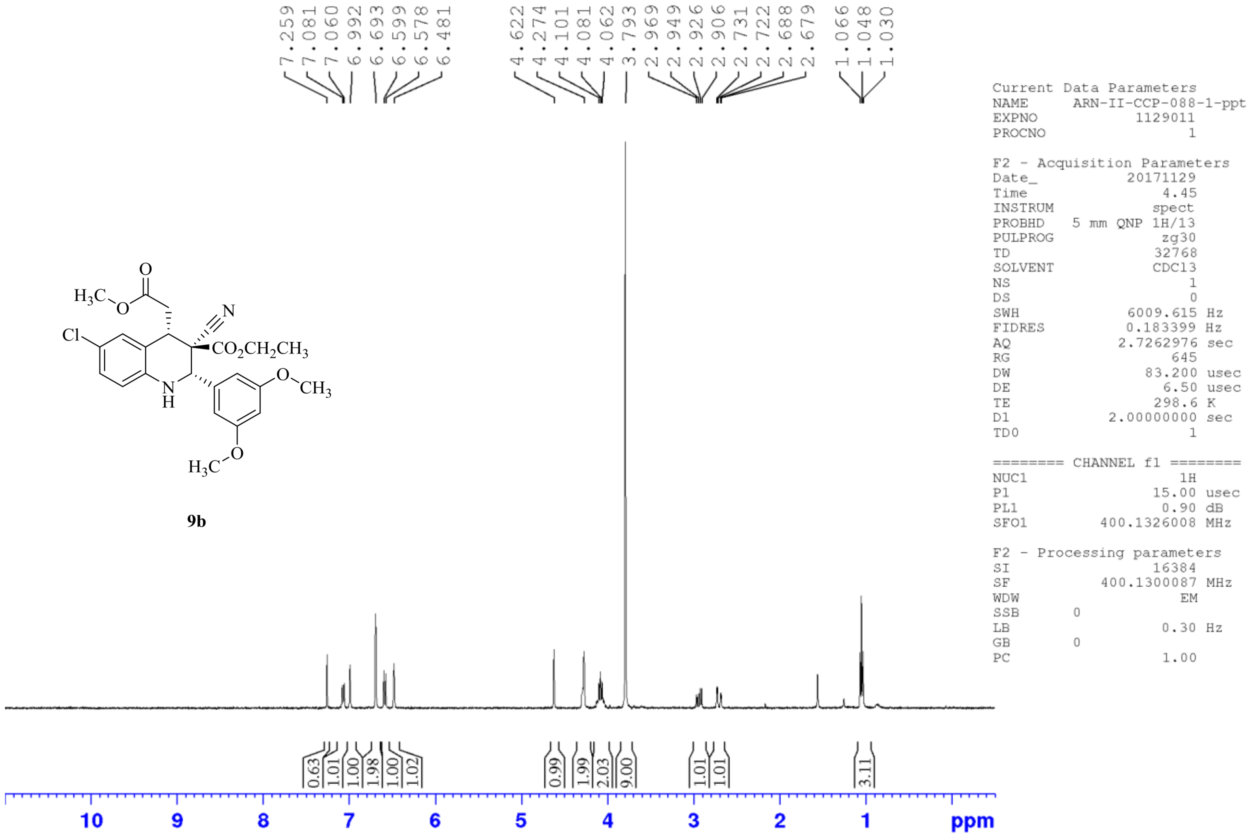

Racemate of ethyl 6-chloro-3-cyano-4-(2-methoxy-2-oxoethyl)-2-(2-methoxyphenyl)-1,2,3,4-tetrahydroquinoline-3-carboxylate (**9b**)

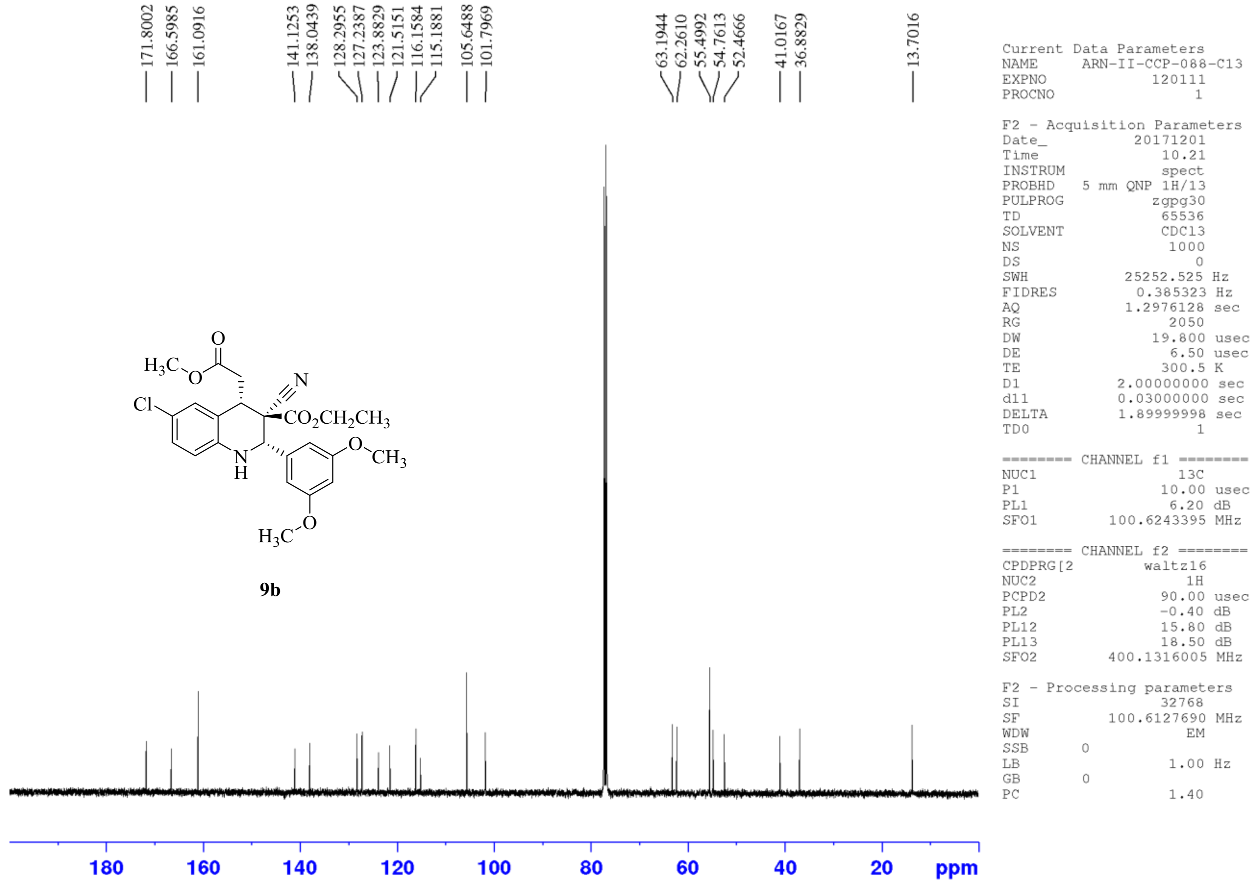

Racemate of ethyl 6-chloro-3-cyano-4-(2-methoxy-2-oxoethyl)-2-(naphthalen-2-yl)-1,2,3,4-tetrahydroquinoline-3-carboxylate (**9c**)

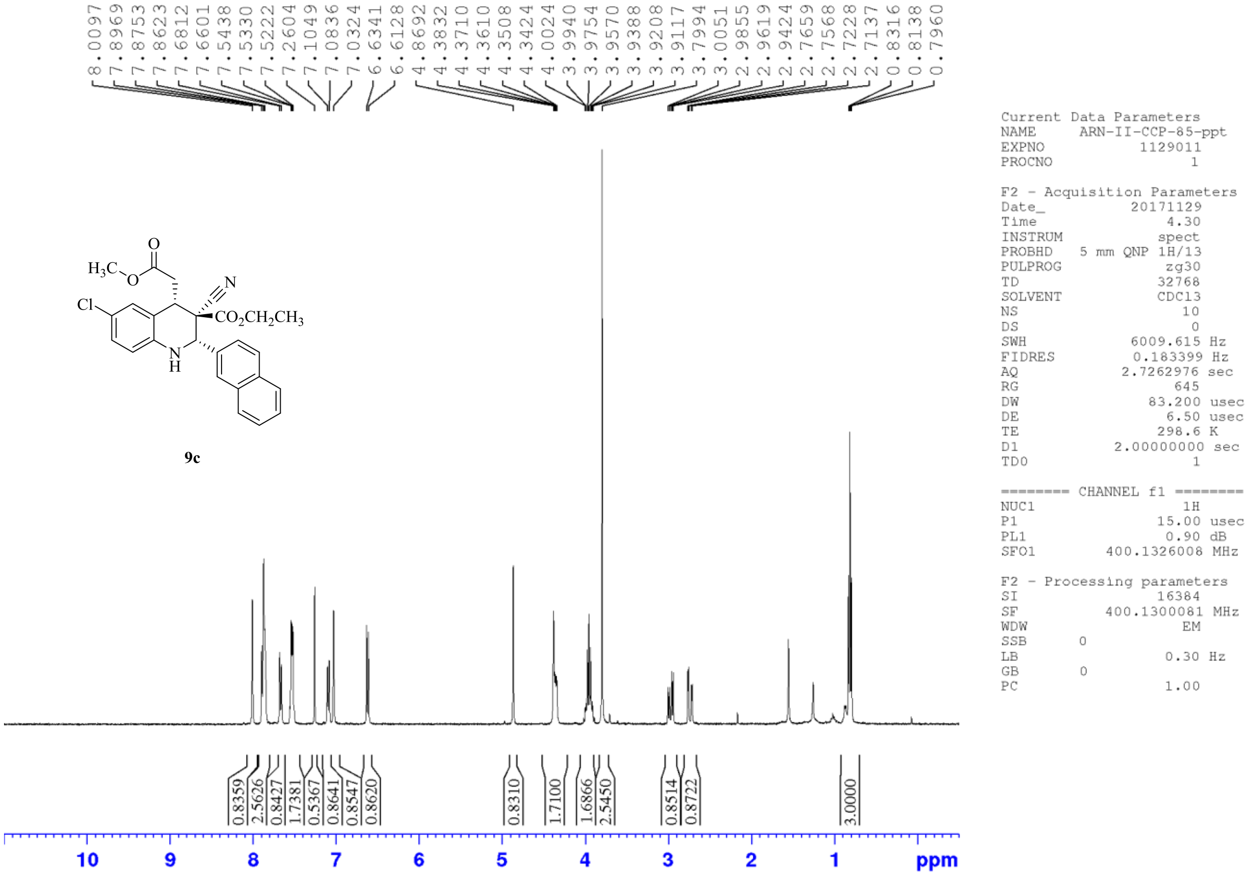

Racemate of ethyl 6-chloro-3-cyano-4-(2-methoxy-2-oxoethyl)-2-(naphthalen-2-yl)-1,2,3,4-tetrahydroquinoline-3-carboxylate (**9c**)

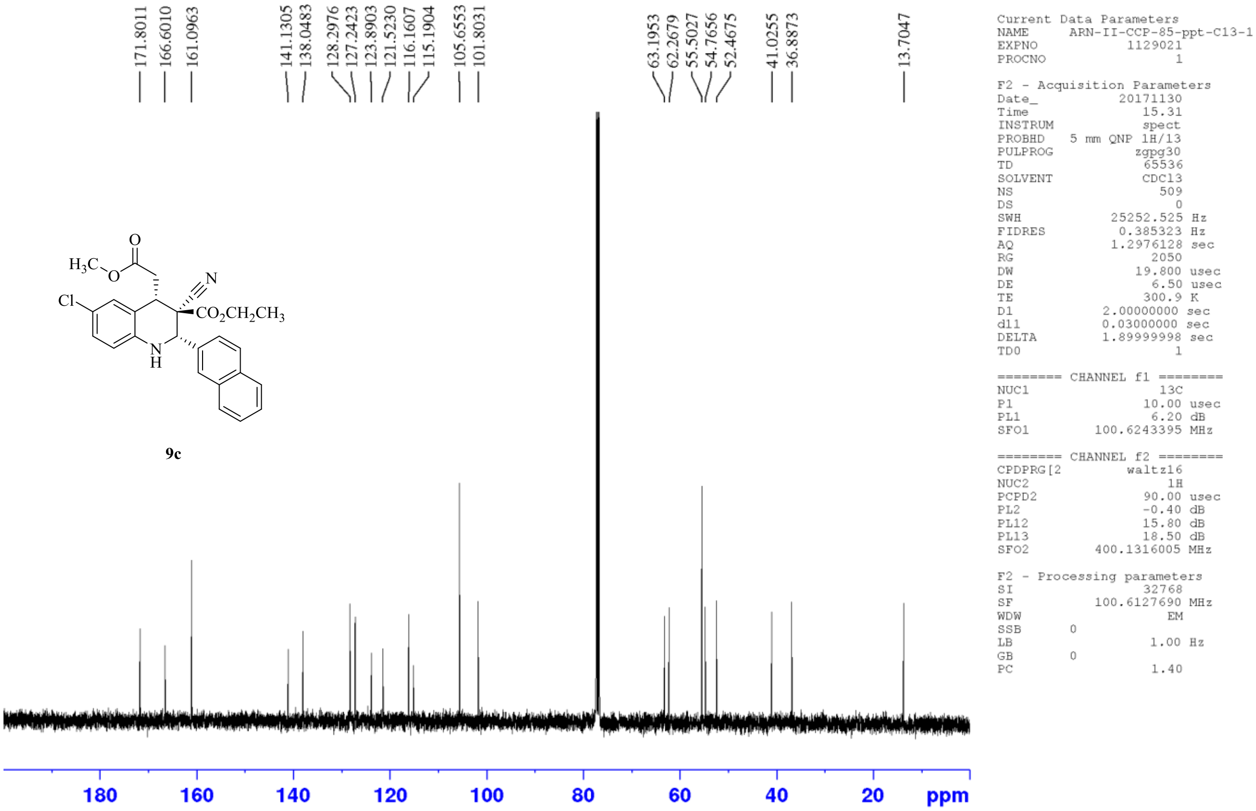

Racemate of ethyl 6-chloro-3-cyano-4-(2-methoxy-2-oxoethyl)-2-(naphthalen-1-yl)-1,2,3,4-tetrahydroquinoline-3-carboxylate (**9d**)

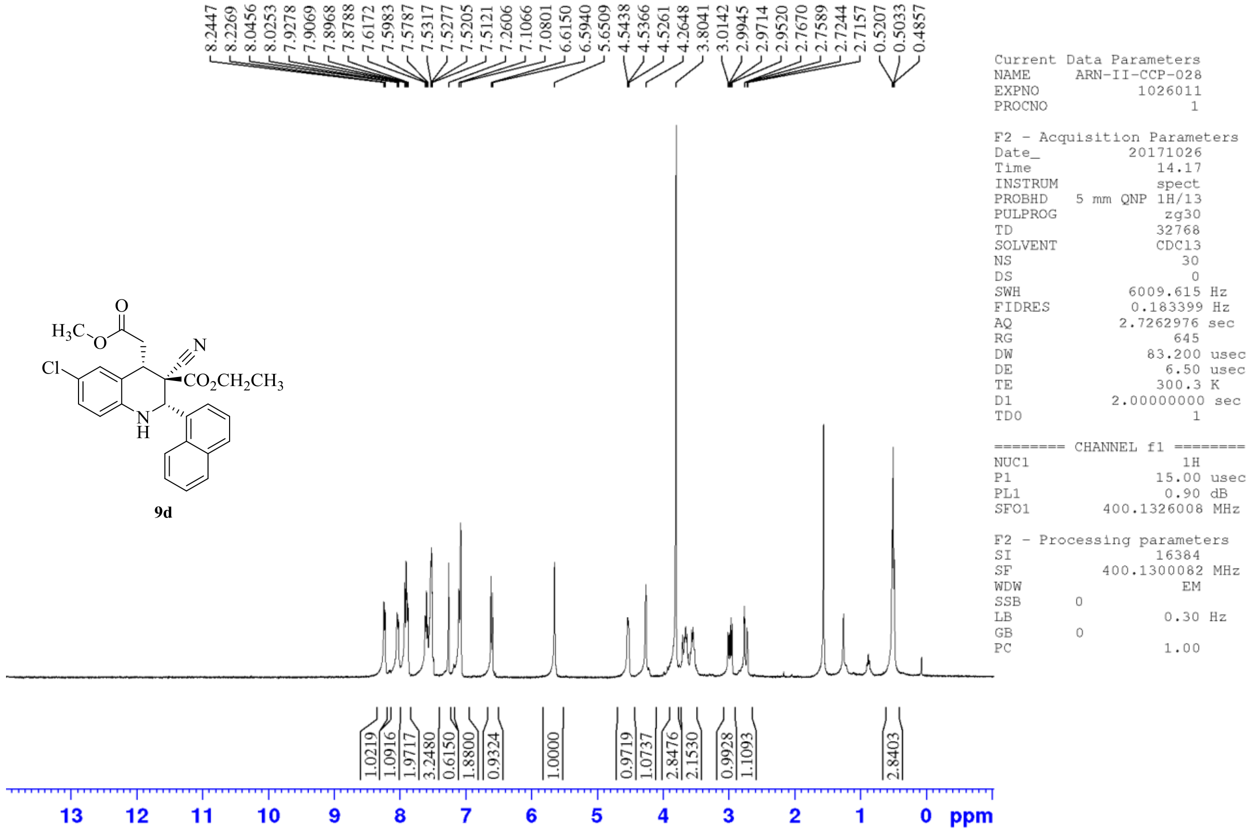

Racemate of ethyl 6-chloro-3-cyano-4-(2-methoxy-2-oxoethyl)-2-(naphthalen-1-yl)-1,2,3,4-tetrahydroquinoline-3-carboxylate (**9d**)

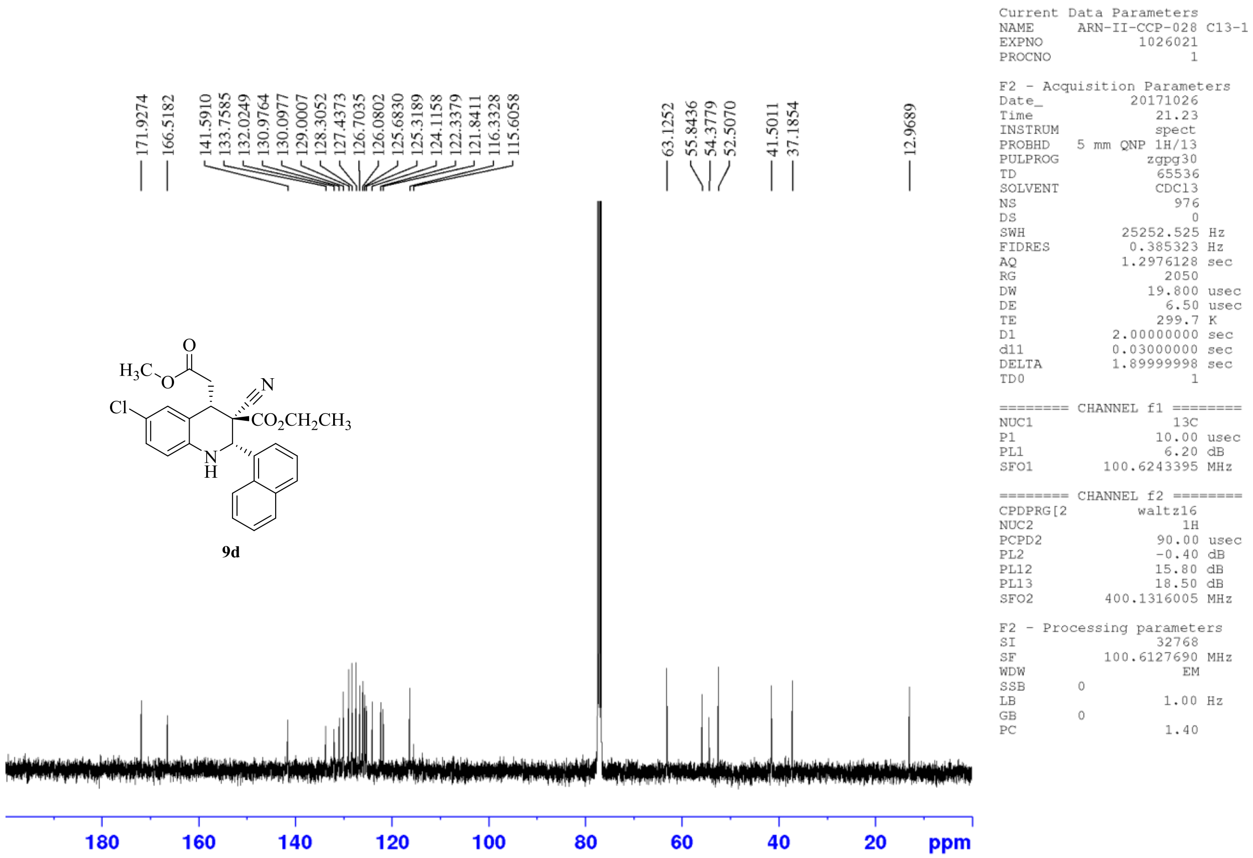

Racemeate of ethyl 6-chloro-3-cyano-4-(2-methoxy-2-oxoethyl)-2-(pyridin-2-yl)-1,2,3,4-tetrahydroquinoline-3-carboxylate (**9e**)

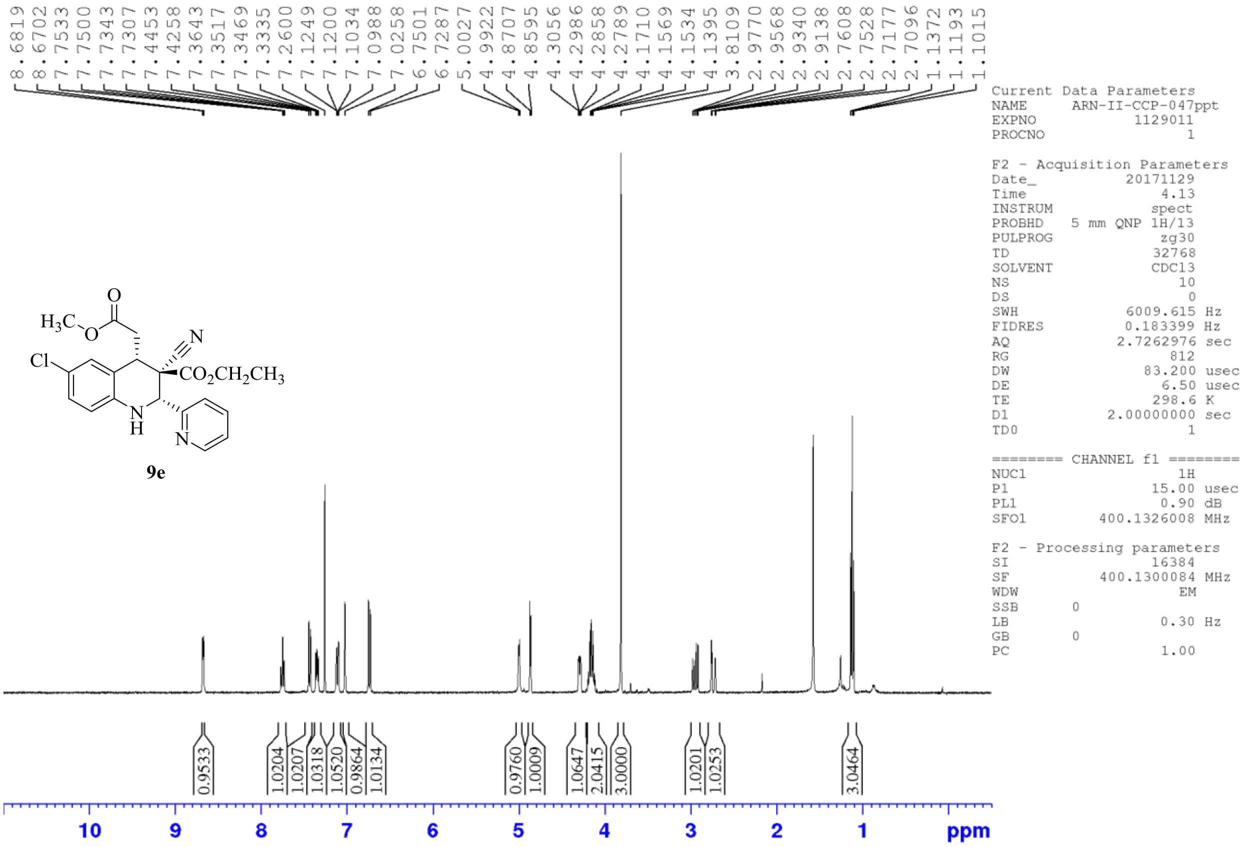

Racemeate of ethyl 6-chloro-3-cyano-4-(2-methoxy-2-oxoethyl)-2-(pyridin-2-yl)-1,2,3,4-tetrahydroquinoline-3-carboxylate (**9e**)

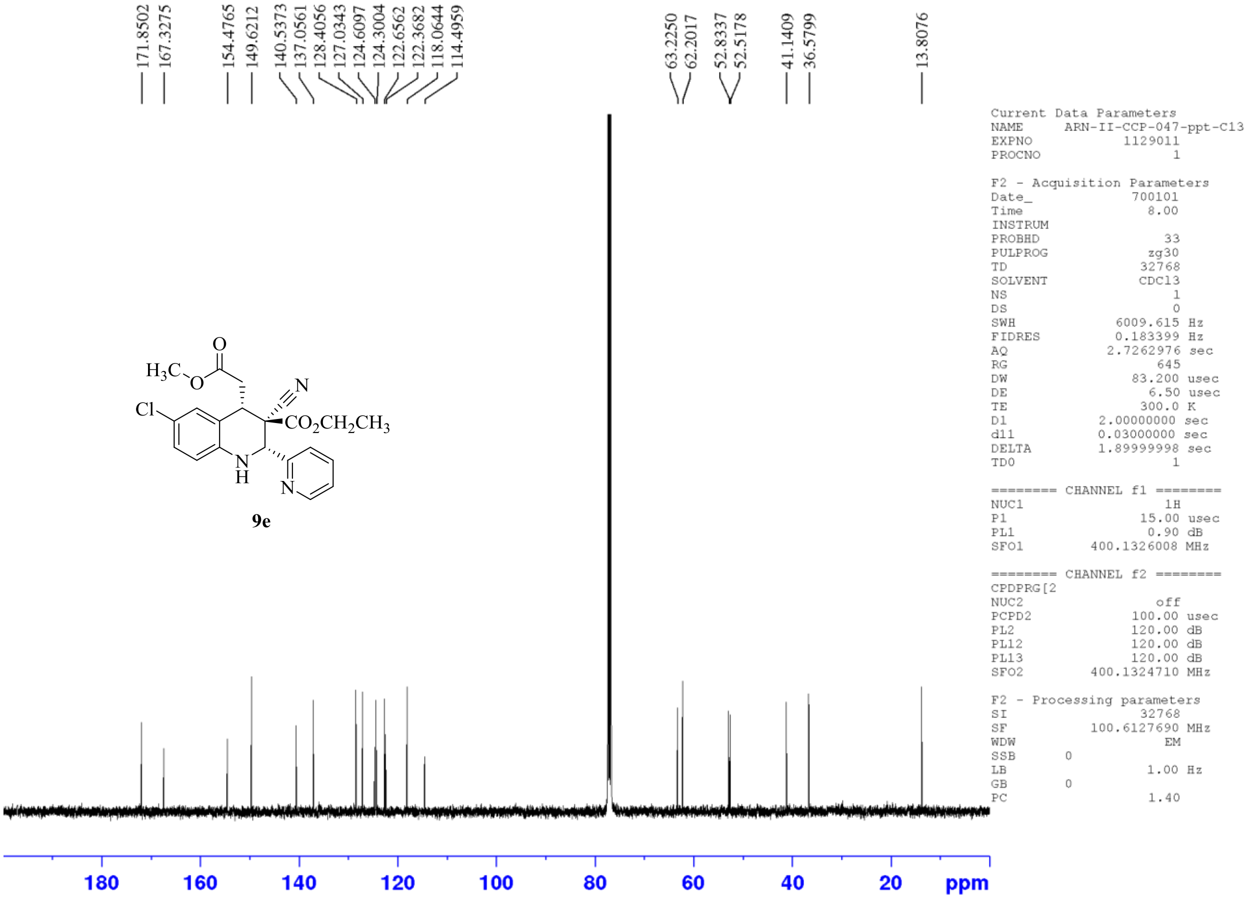

Racemate of ethyl 6-chloro-3-cyano-4-(2-methoxy-2-oxoethyl)-2-(thiophen-3-yl)-1,2,3,4-tetrahydroquinoline-3-carboxylate (**9f**)

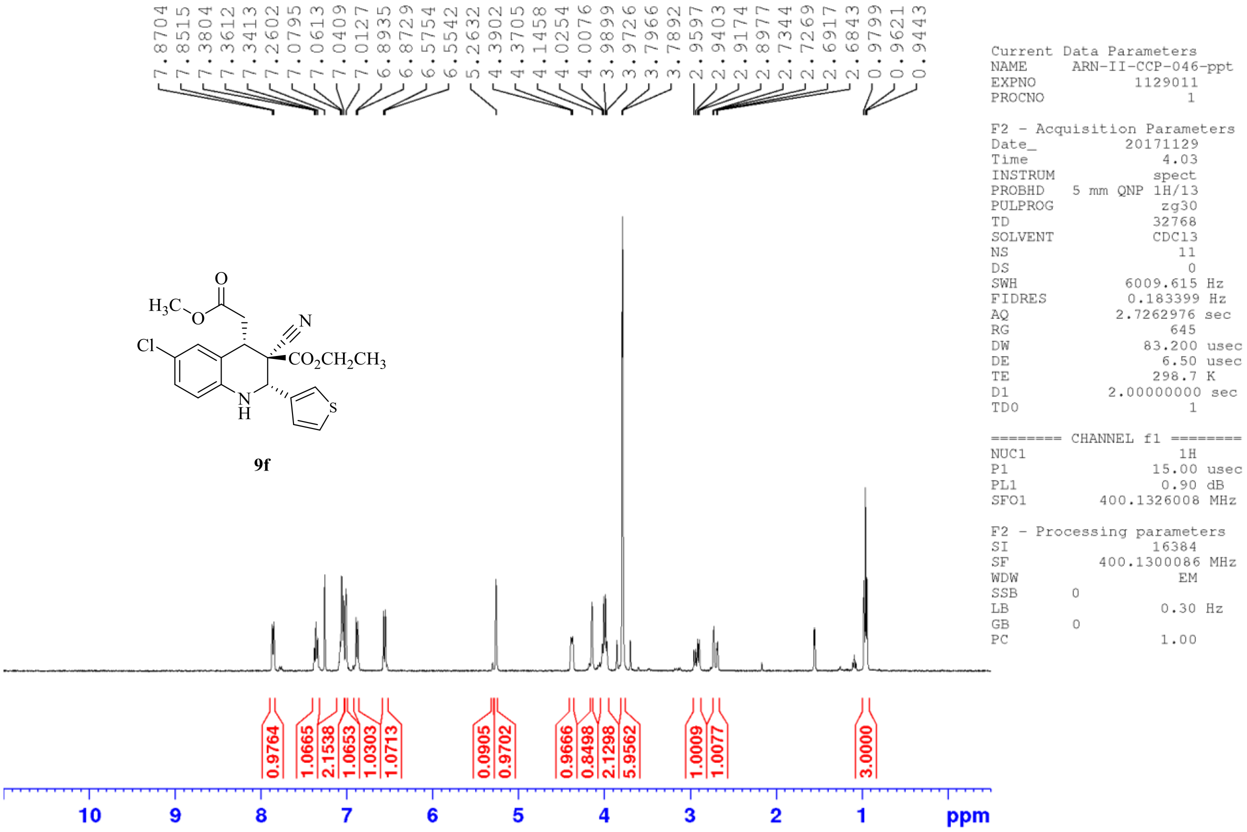

Racemate of ethyl 6-chloro-3-cyano-4-(2-methoxy-2-oxoethyl)-2-(thiophen-3-yl)-1,2,3,4-tetrahydroquinoline-3-carboxylate (**9f**)

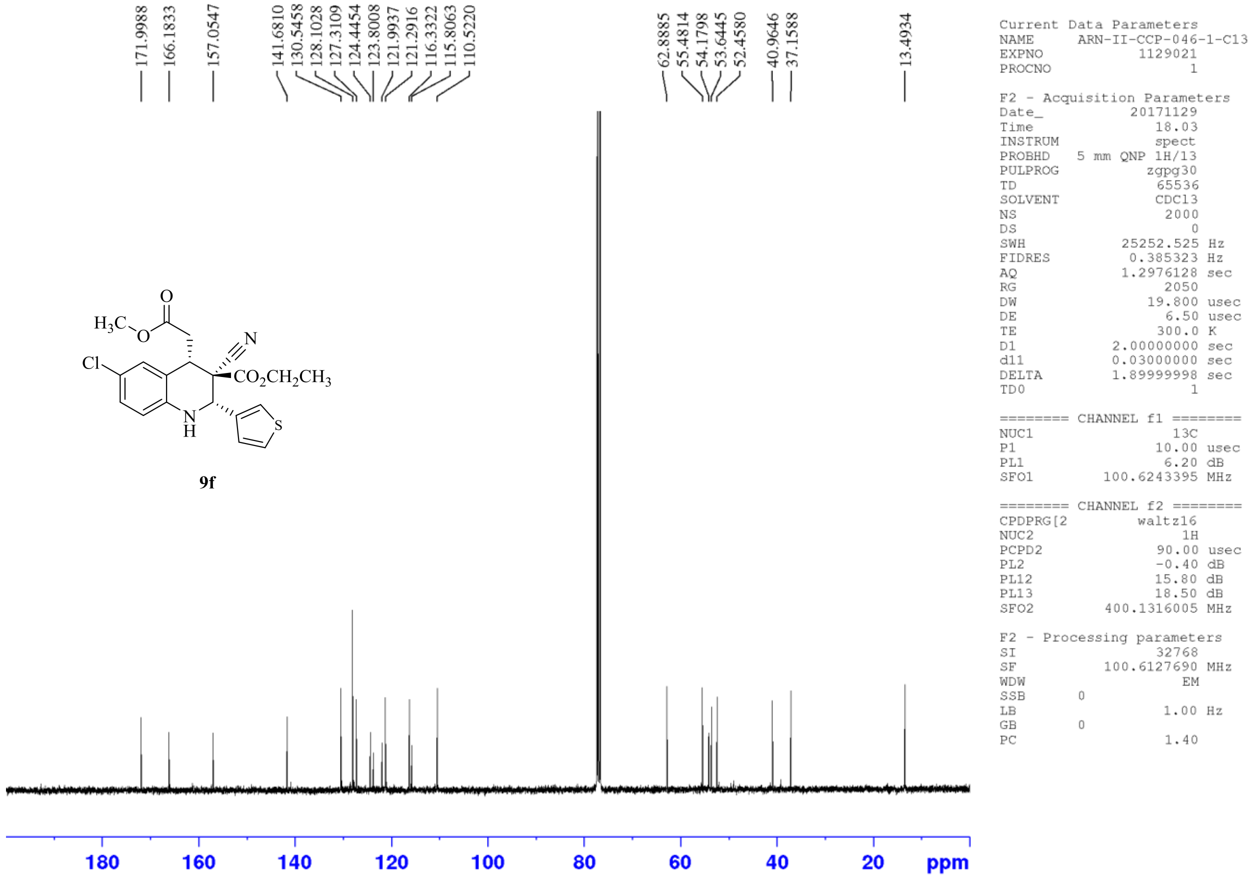

Racemate of ethyl 6-chloro-3-cyano-4-(2-methoxy-2-oxoethyl)-2-(thiophen-2-yl)-1,2,3,4-tetrahydroquinoline-3-carboxylate (**9g**)

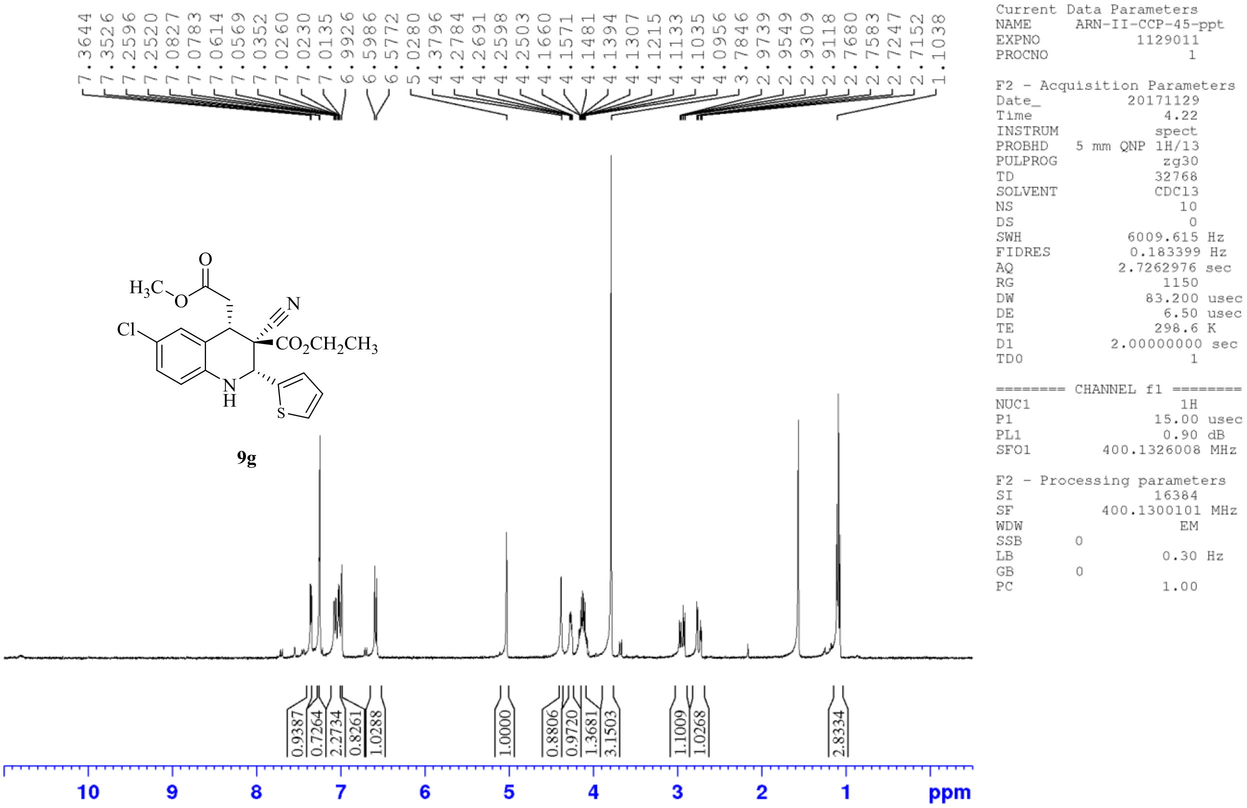

Racemate of ethyl 6-chloro-3-cyano-4-(2-methoxy-2-oxoethyl)-2-(thiophen-2-yl)-1,2,3,4-tetrahydroquinoline-3-carboxylate (**9g**)

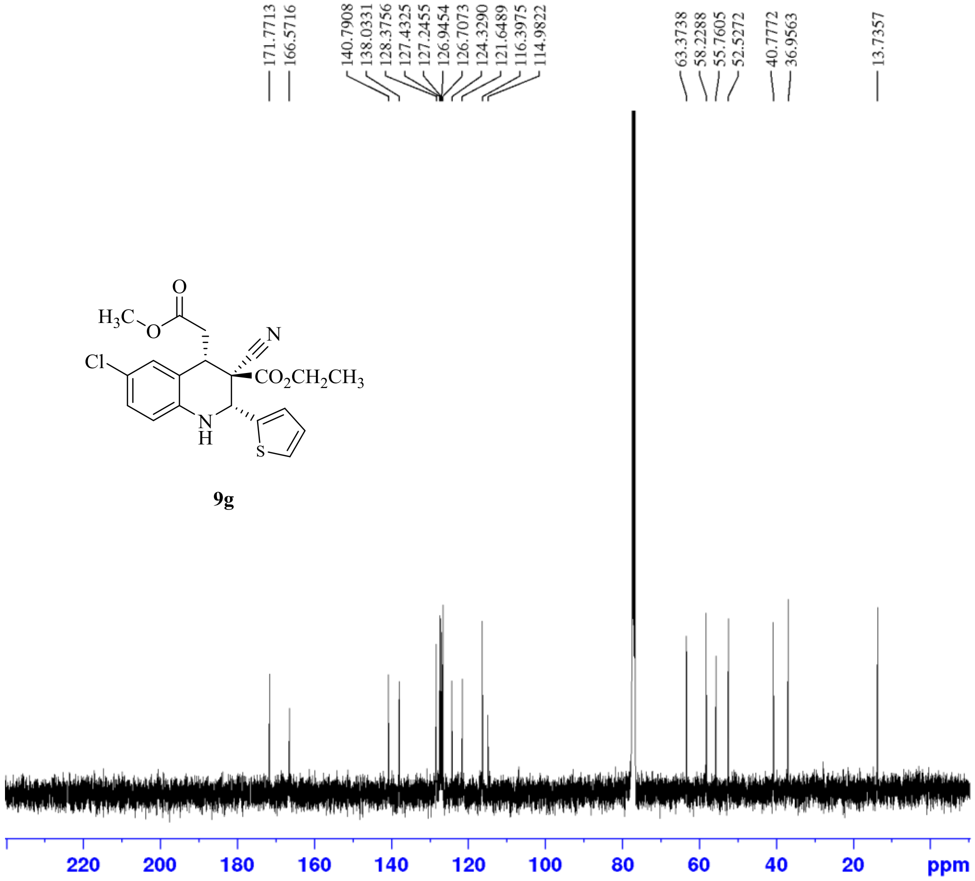

9g

```
Current Data Parameters
NAME     ARN-II-CCP-045-1-C13
EXPNO              1129021
PROCNO                   1

F2 - Acquisition Parameters
Date_             20171129
Time                 16.06
INSTRUM              spect
PROBHD   5 mm QNP 1H/13
PULPROG            zgpg30
TD                   65536
SOLVENT              CDCl3
NS                    1733
DS                       0
SWH             25252.525 Hz
FIDRES           0.385323 Hz
AQ              1.2976128 sec
RG                    2050
DW                  19.800 usec
DE                    6.50 usec
TE                   296.4 K
D1              2.00000000 sec
d11             0.03000000 sec
DELTA           1.89999998 sec
TD0                      1

======== CHANNEL f1 ========
NUC1                   13C
P1                   10.00 usec
PL1                   6.20 dB
SFO1           100.6243395 MHz

======== CHANNEL f2 ========
CPDPRG[2           waltz16
NUC2                    1H
PCPD2                90.00 usec
PL2                  -0.40 dB
PL12                 15.80 dB
PL13                 18.50 dB
SFO2           400.1316005 MHz

F2 - Processing parameters
SI                   32768
SF             100.6127690 MHz
WDW                     EM
SSB      0
LB                    1.00 Hz
GB       0
PC                    1.40
```

Racemate of 3-ethyl, 6-methyl 3-cyano-4-(2-methoxy-2-oxoethyl)-2-(naphthalen-2-yl)-1,2,3,4-tetrahydroquinoline-3,6-dicarboxylate (**9h**)

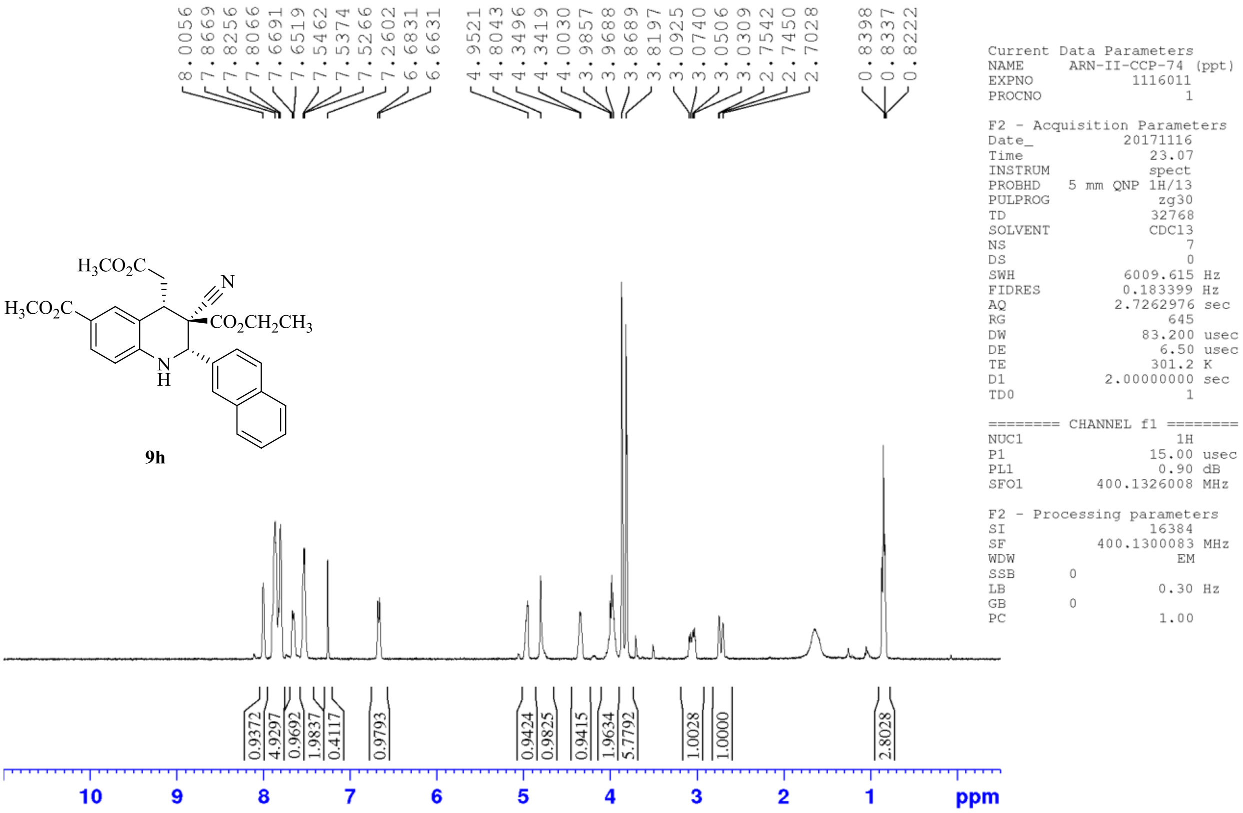

Racemate of 3-ethyl, 6-methyl 3-cyano-4-(2-methoxy-2-oxoethyl)-2-(naphthalen-2-yl)-1,2,3,4-tetrahydroquinoline-3,6-dicarboxylate (**9h**)

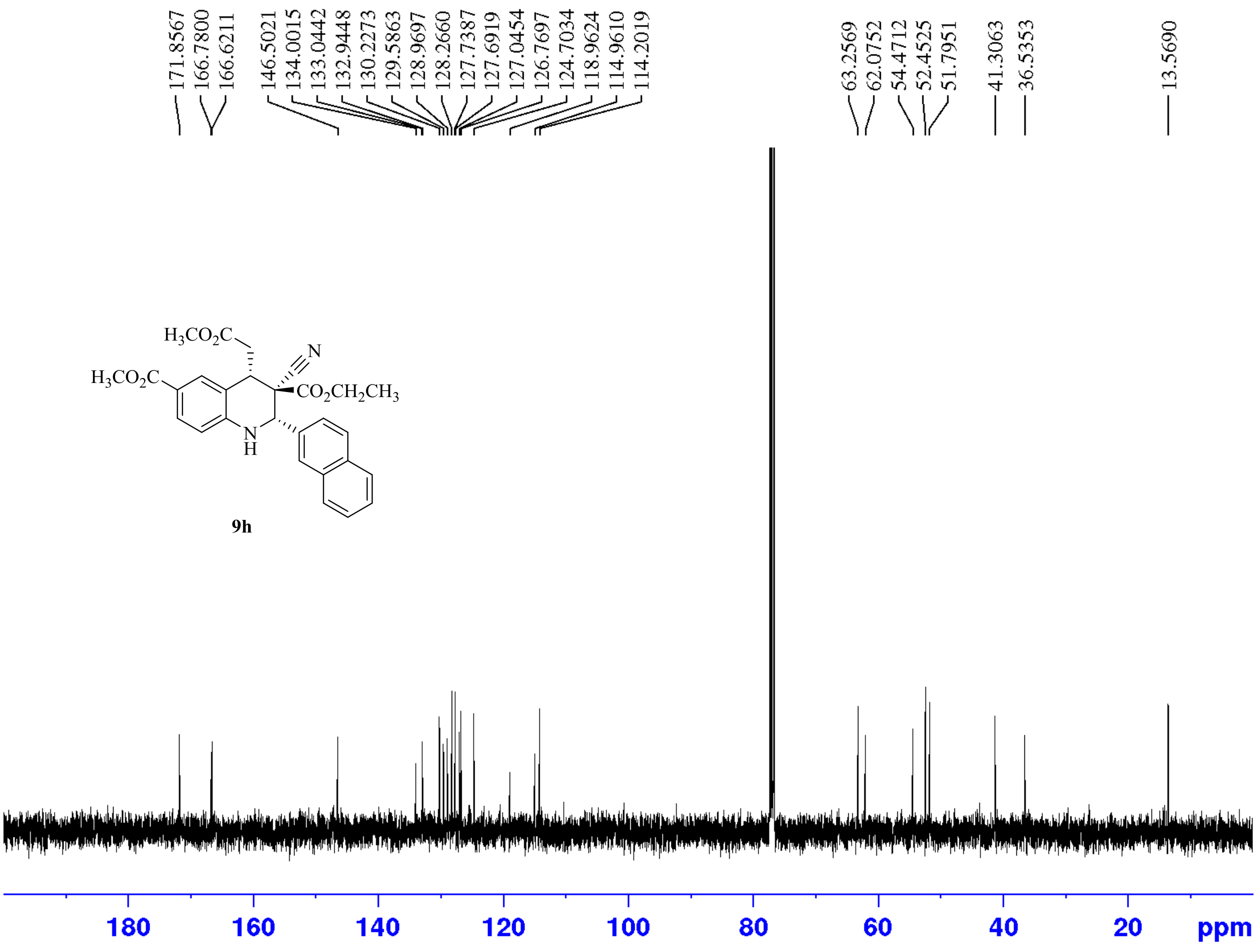

9h

Racemate of 3-ethyl, 6-methyl 3-cyano-4-(2-methoxy-2-oxoethyl)-2-(naphthalen-1-yl)-1,2,3,4-tetrahydroquinoline-3,6-dicarboxylate (**9i**).

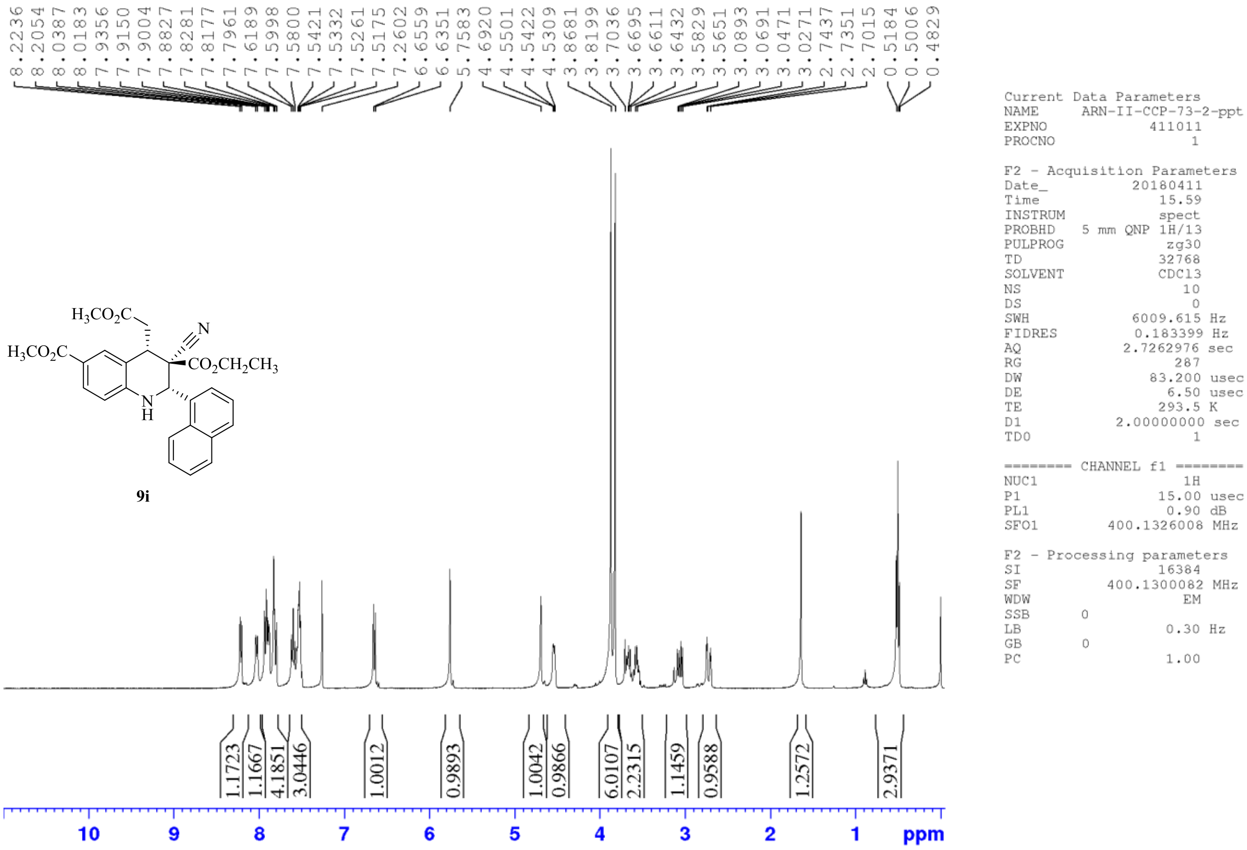

Racemate of 3-ethyl 6-methyl 3-cyano-4-(2-methoxy-2-oxoethyl)-2-(naphthalen-1-yl)-1,2,3,4-tetrahydroquinoline-3,6-dicarboxylate (**9i**).

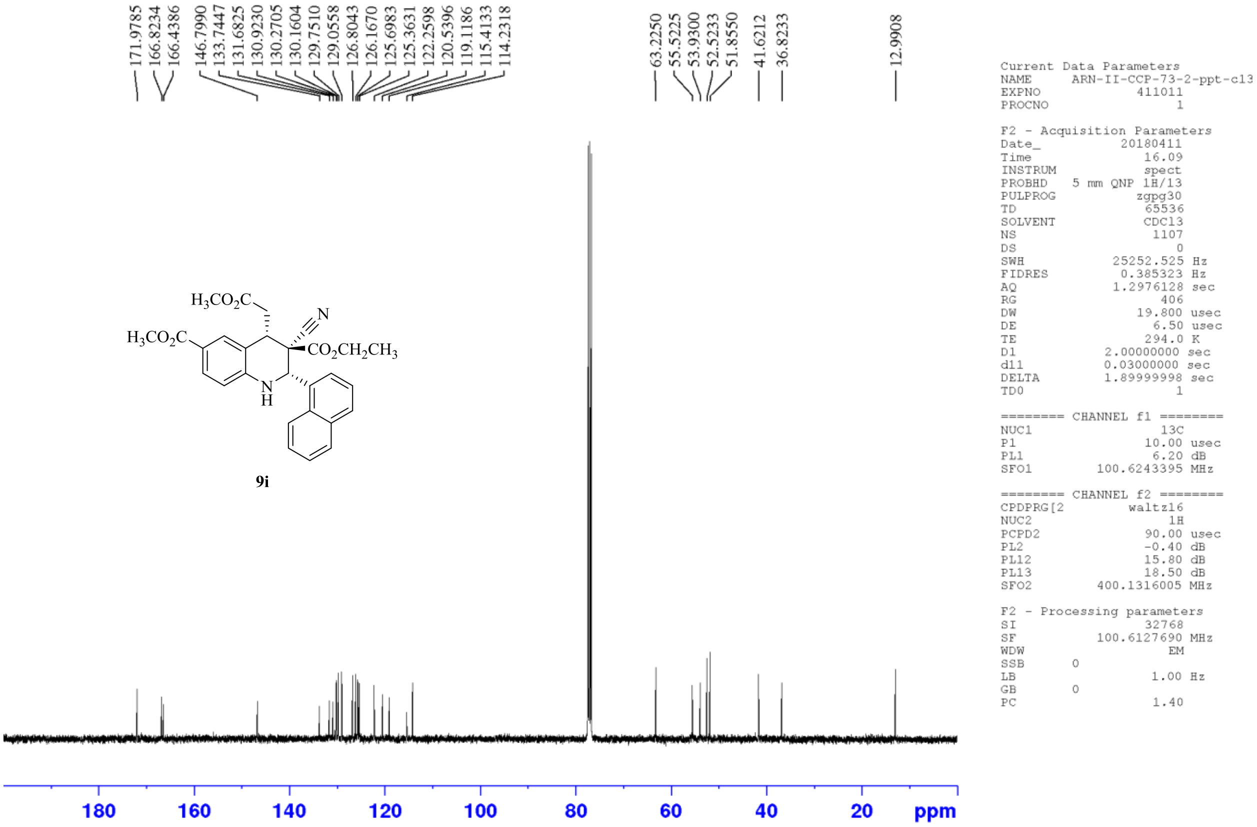

Racemate of 3-ethyl, 4,6-dimethyl 3-cyano-2-(2-methoxyphenyl)-1,2,3,4-tetrahydroquinoline-3,4,6-tricarboxylate (**9j**)

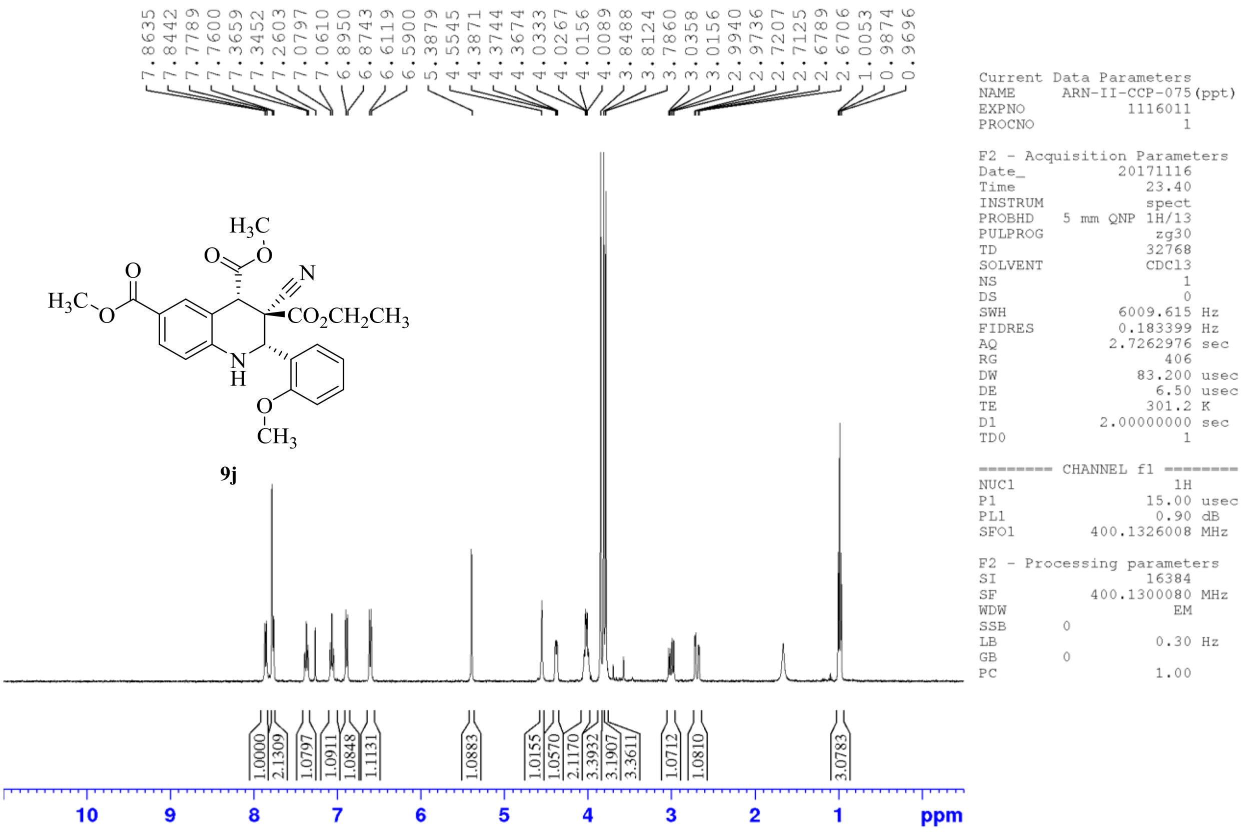

Racemate of 3-ethyl, 4,6-dimethyl 3-cyano-2-(2-methoxyphenyl)-1,2,3,4-tetrahydroquinoline-3,4,6-tricarboxylate (**9j**)

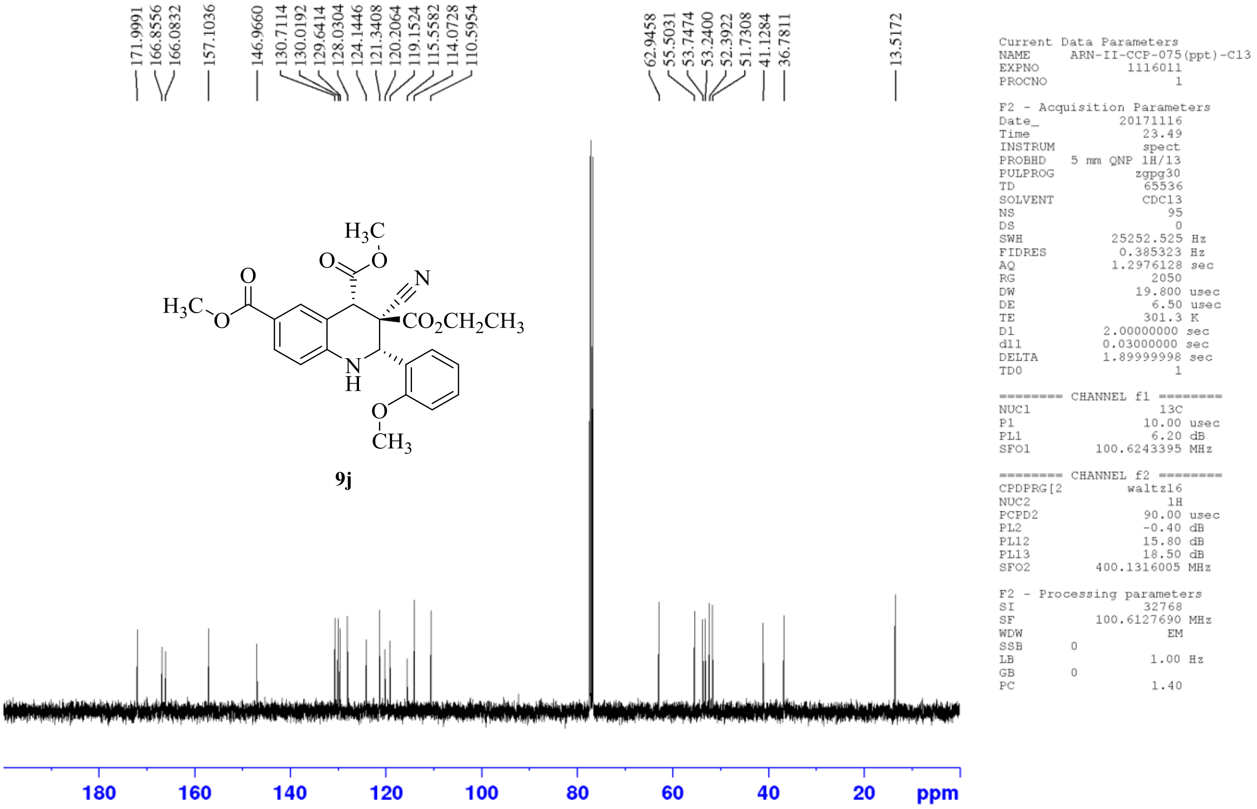

Racemate of 3-ethyl, 6-methyl 3-cyano-2-(3,5-dimethoxyphenyl)-4-(2-methoxy-2-oxoethyl)-1,2,3,4-tetrahydroquinoline-3,6-dicarboxylate (**9k**)

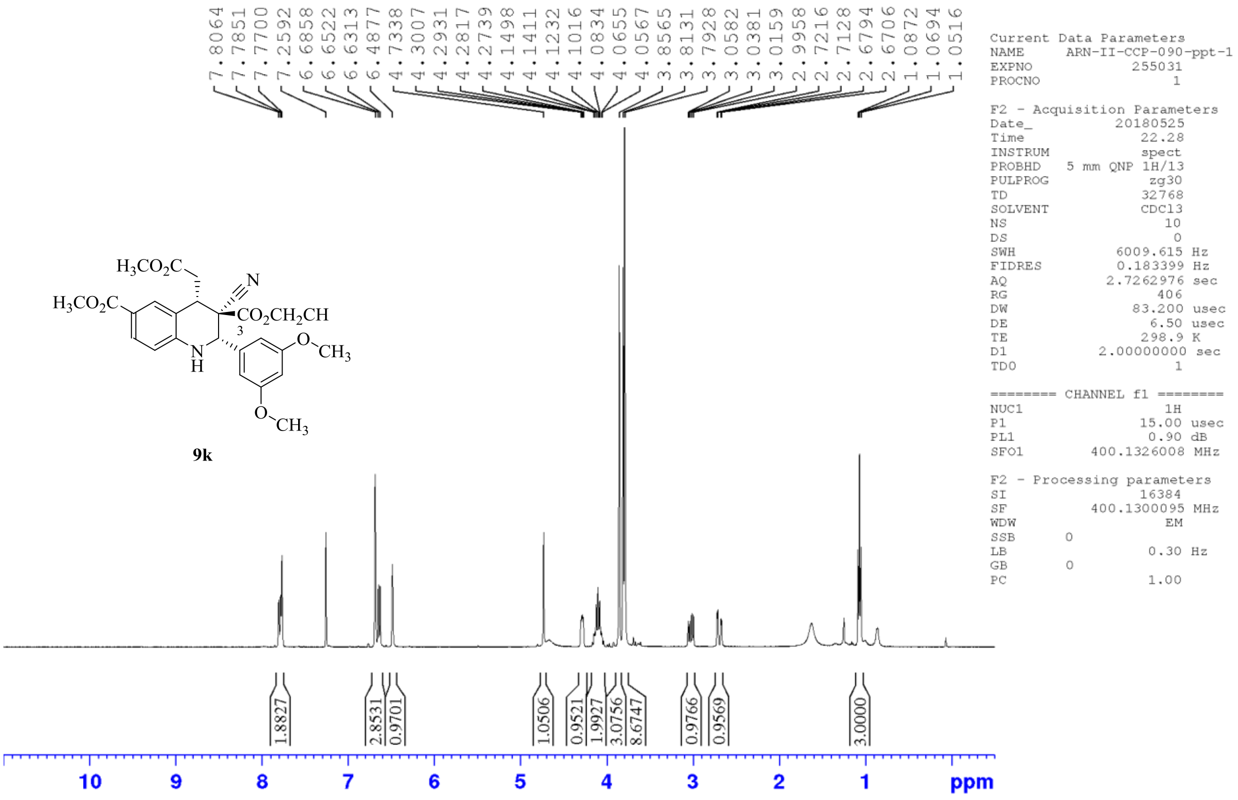

Racemate of 3-ethyl, 6-methyl 3-cyano-2-(3,5-dimethoxyphenyl)-4-(2-methoxy-2-oxoethyl)-1,2,3,4-tetrahydroquinoline-3,6-dicarboxylate (**9k**)

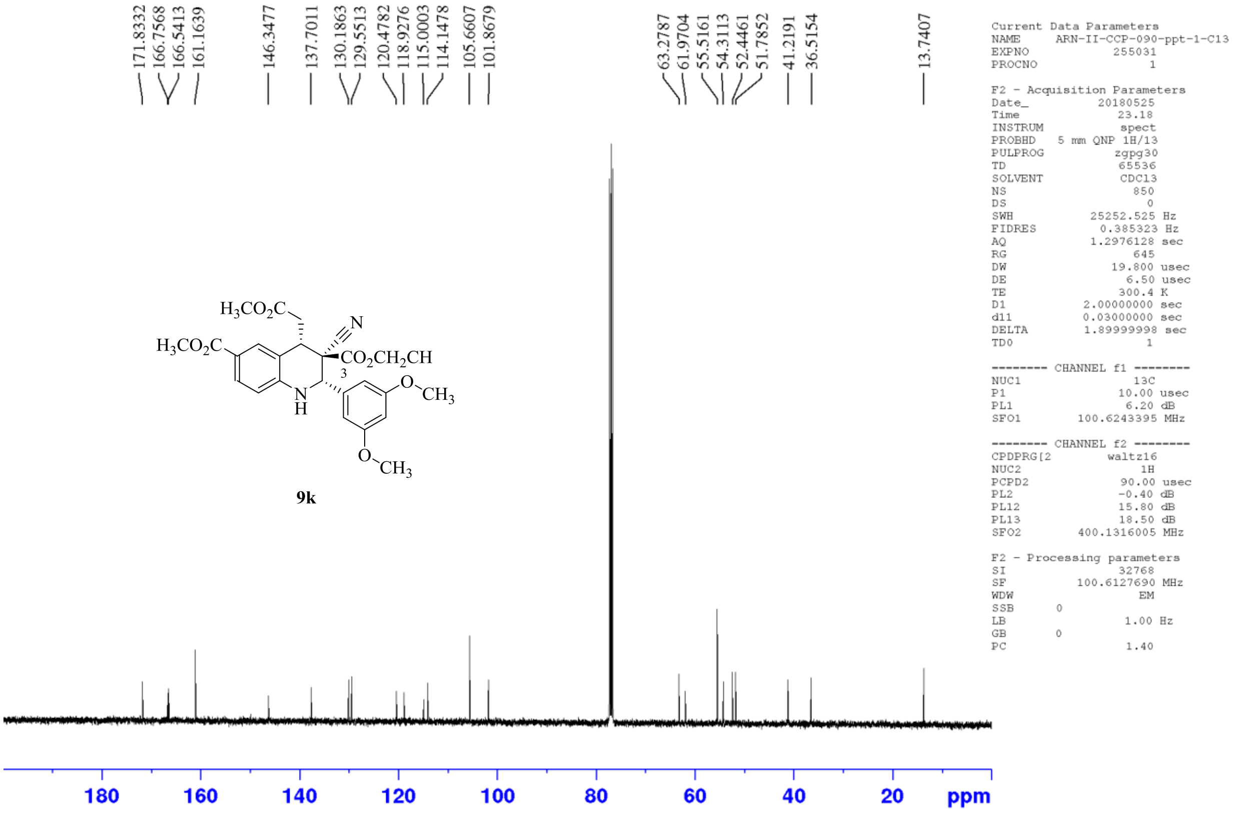

Racemate of ethyl 3-cyano-2-(naphthalen-2-yl)-4-(2-oxo-2-phenylethyl)-1,2,3,4-tetrahydroquinoline-3-carboxylate (**9l**)

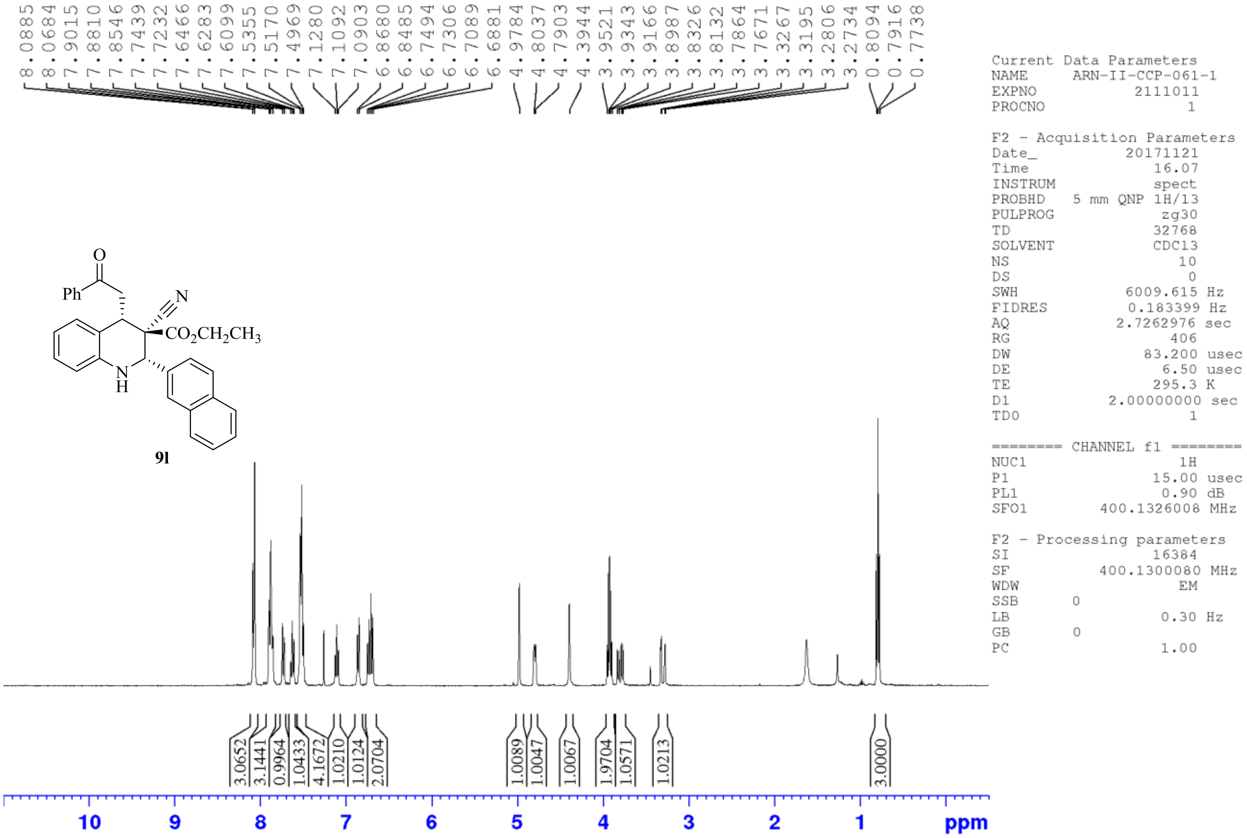

Racemate of ethyl 3-cyano-2-(naphthalen-2-yl)-4-(2-oxo-2-phenylethyl)-1,2,3,4-tetrahydroquinoline-3-carboxylate (**9l**)

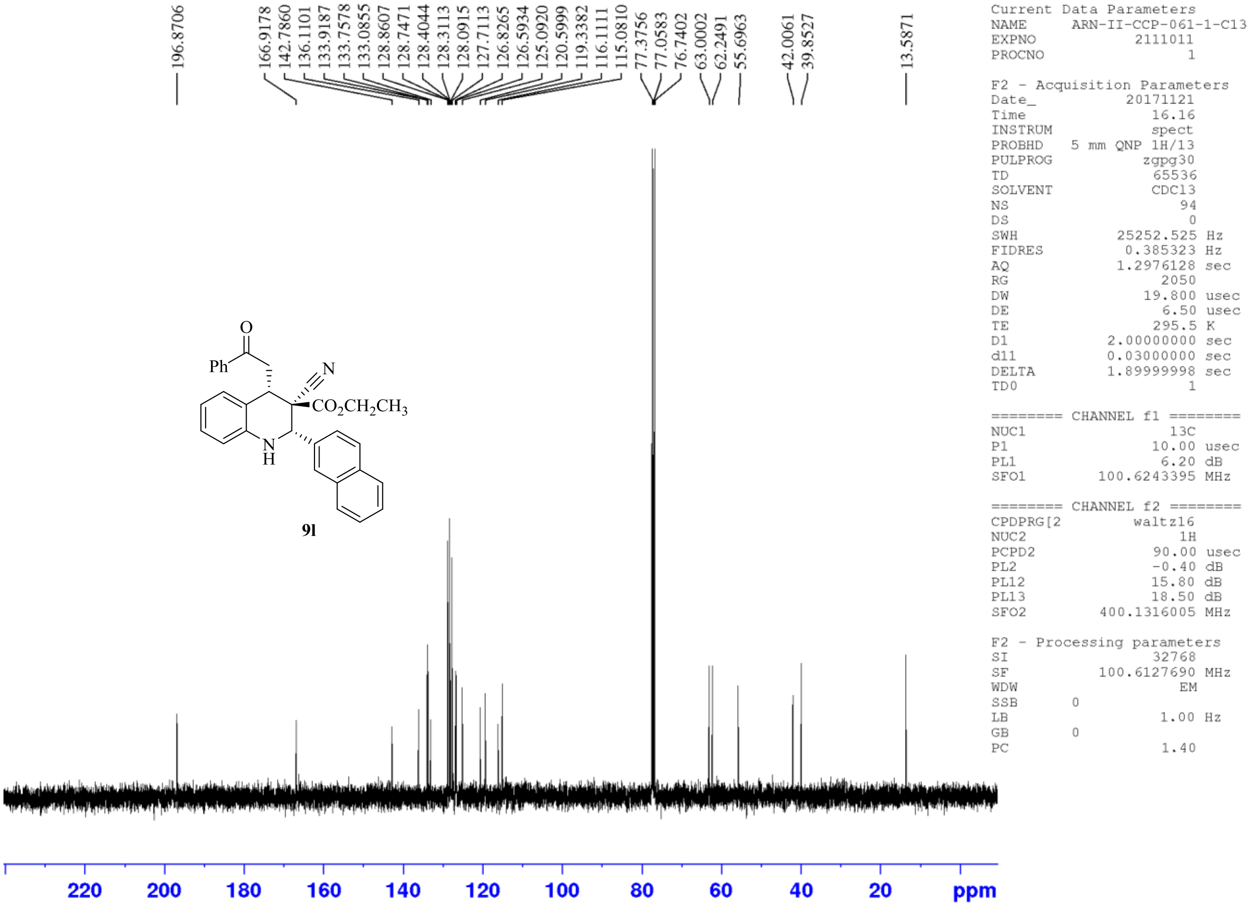

Racemate of ethyl 3-cyano-2-(2-methoxyphenyl)-4-(2-oxo-2-phenylethyl)-1,2,3,4-tetrahydroquinoline-3-carboxylate (**9m**)

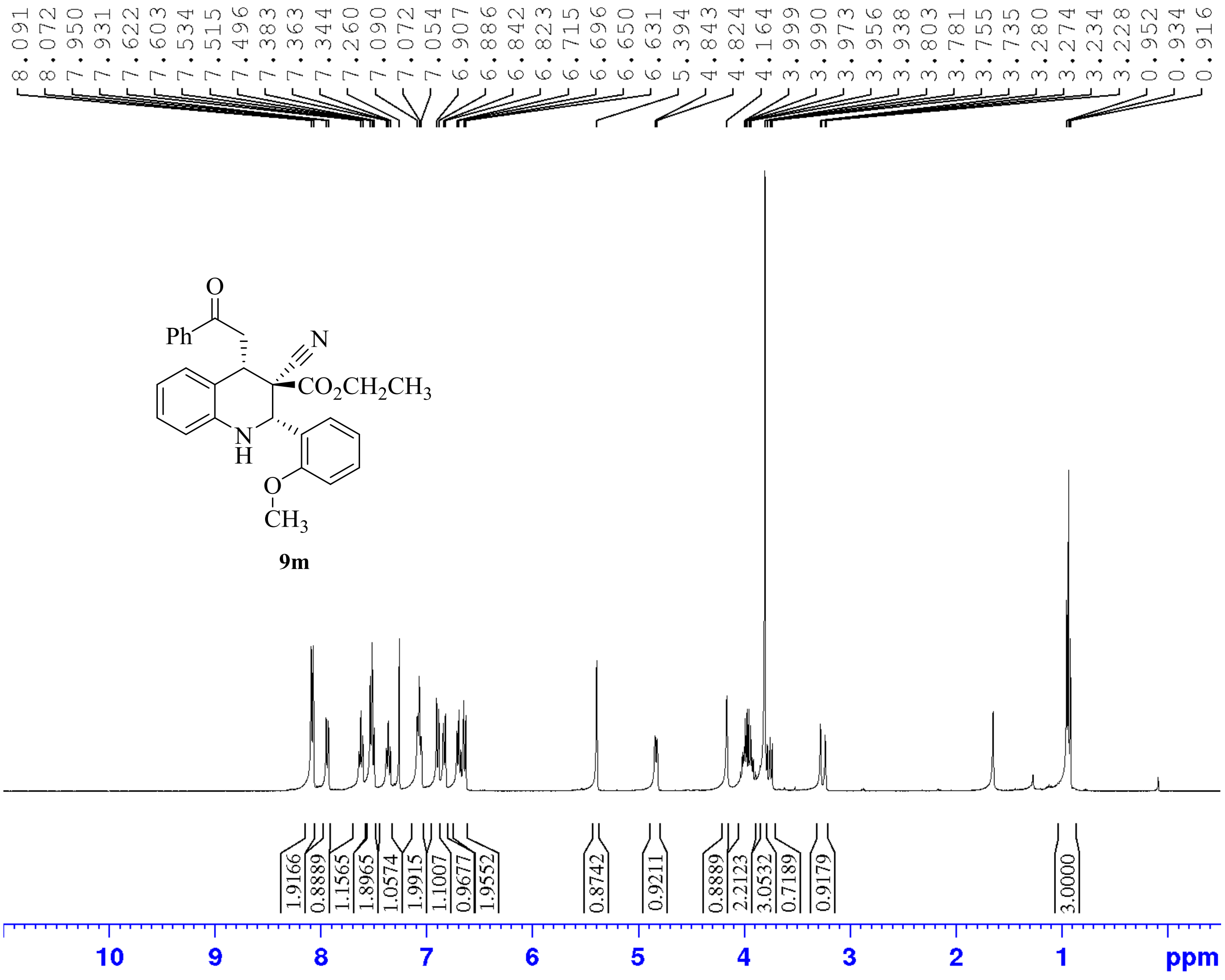

Racemate of ethyl 3-cyano-2-(2-methoxyphenyl)-4-(2-oxo-2-phenylethyl)-1,2,3,4-tetrahydroquinoline-3-carboxylate (**9m**)

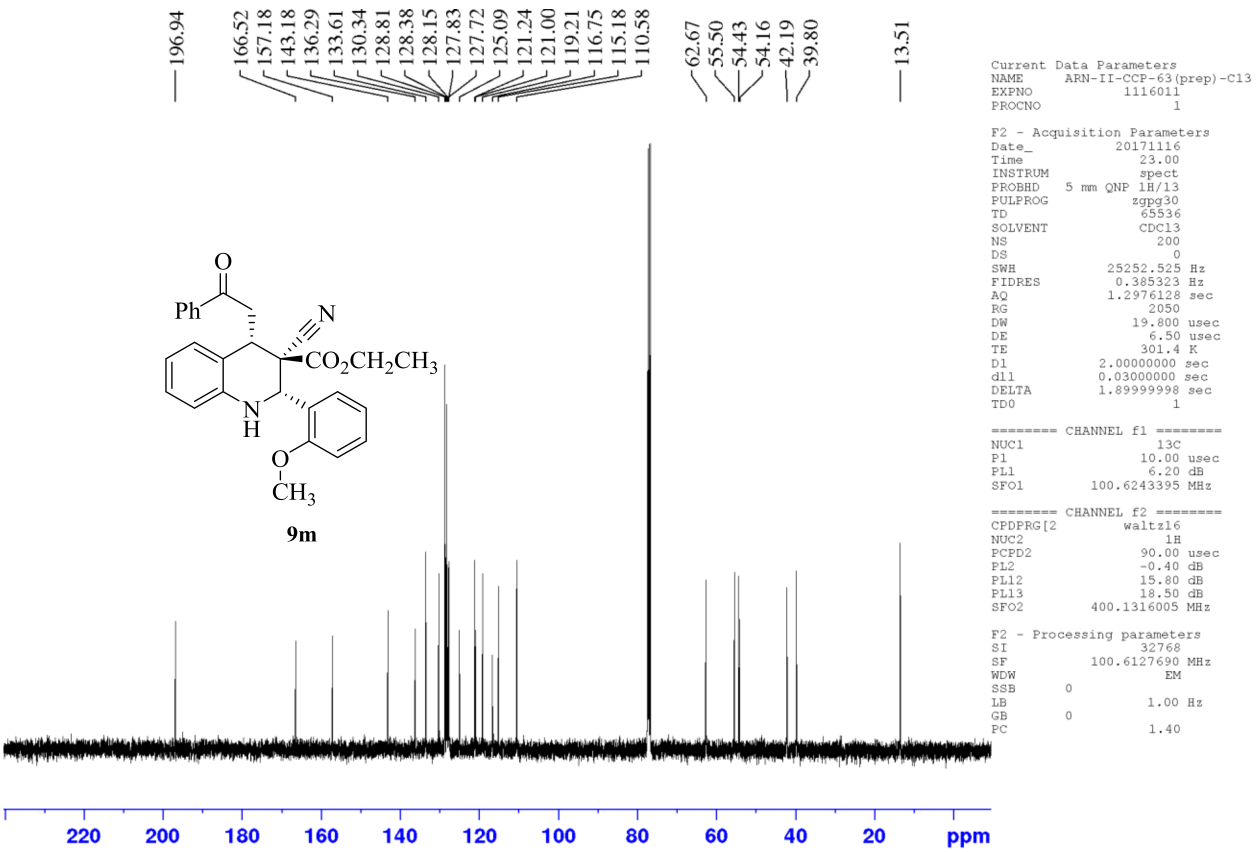

Racemate of ethyl 3-cyano-2-(naphthalen-1-yl)-4-(2-oxo-2-phenylethyl)-1,2,3,4-tetrahydroquinoline-3-carboxylate (**9n**)

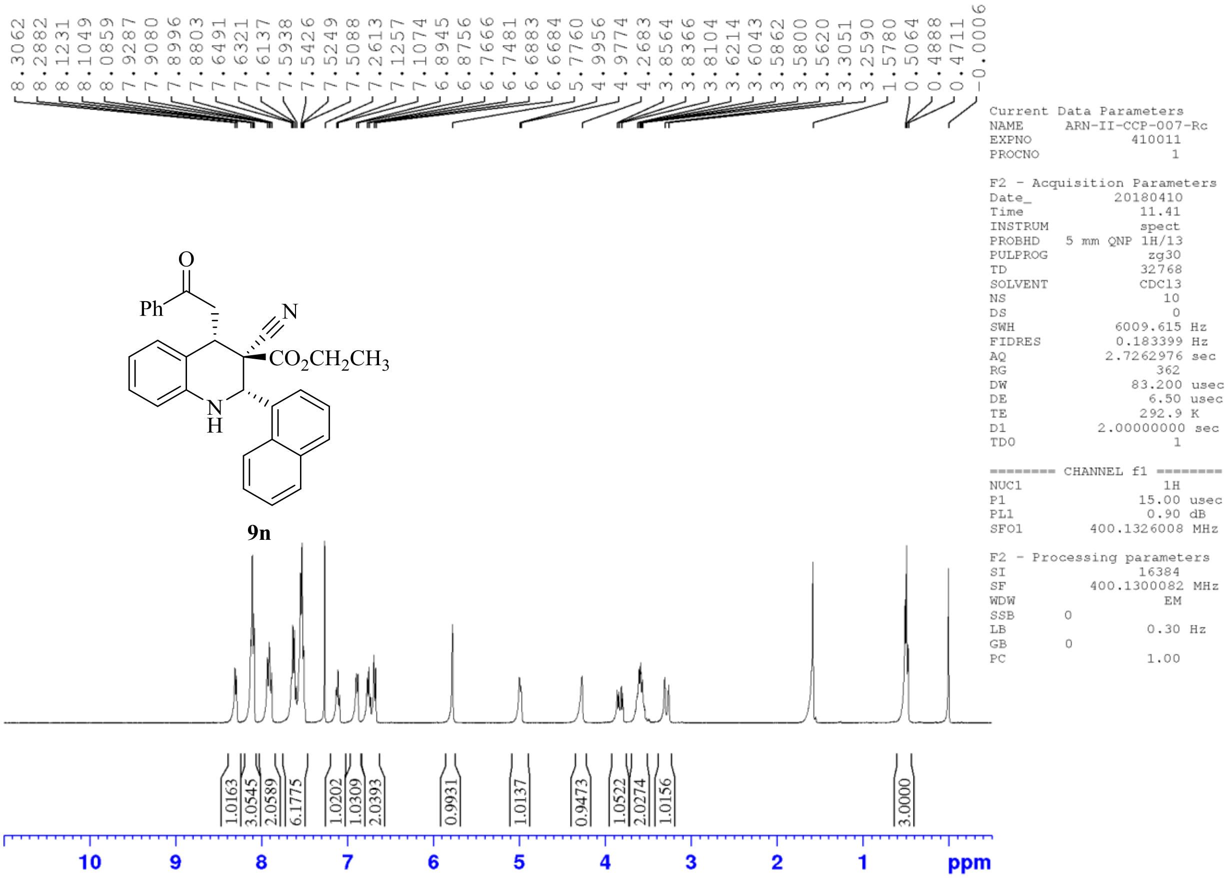

Racemate of ethyl 3-cyano-2-(naphthalen-1-yl)-4-(2-oxo-2-phenylethyl)-1,2,3,4-tetrahydroquinoline-3-carboxylate (**9n**)

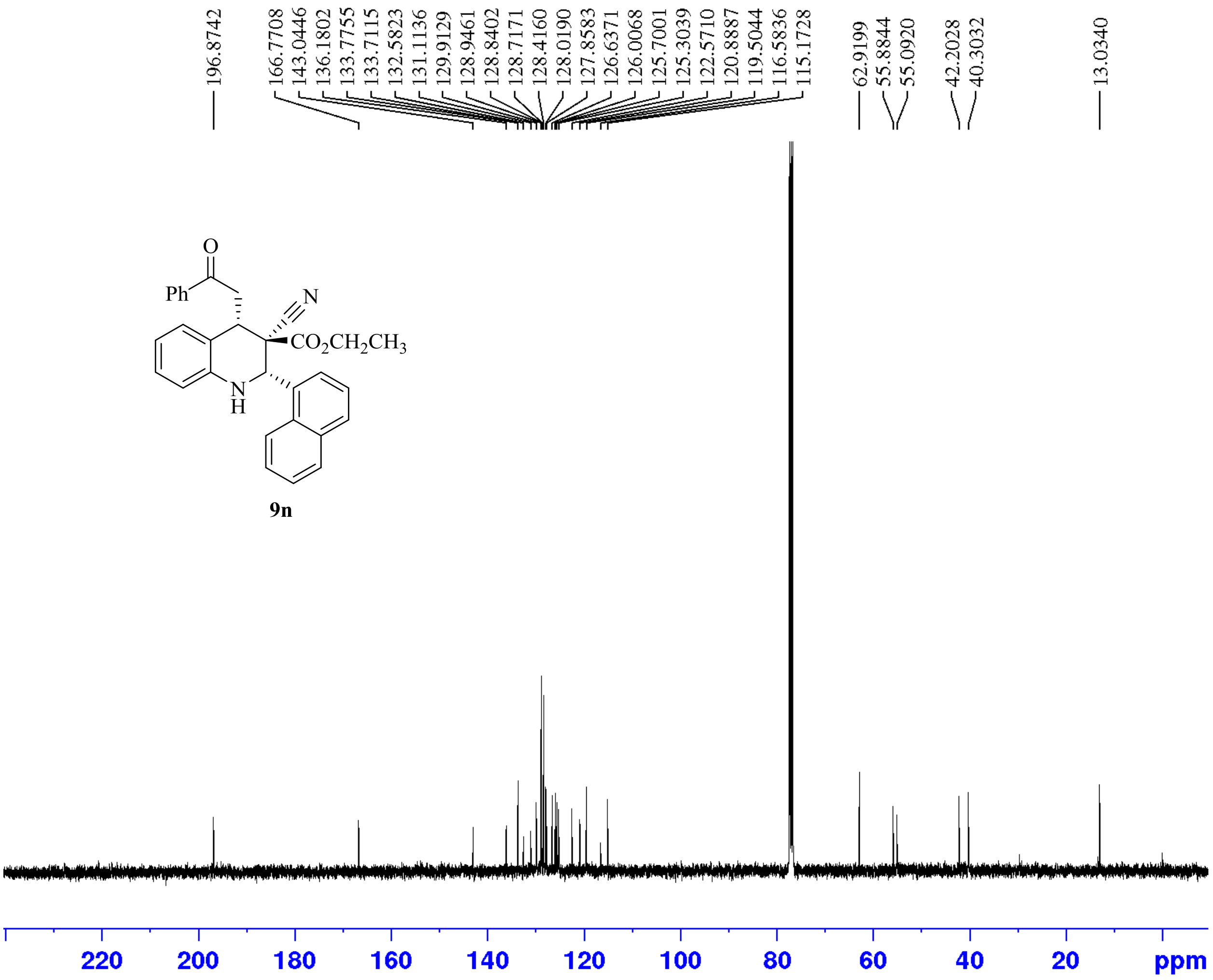

Racemate of (2R,3R,4S)-ethyl 3-cyano-4-(2-oxo-2-phenylethyl)-2-(thiophen-2-yl)-1,2,3,4-tetrahydroquinoline-3-carboxylate (**9o**)

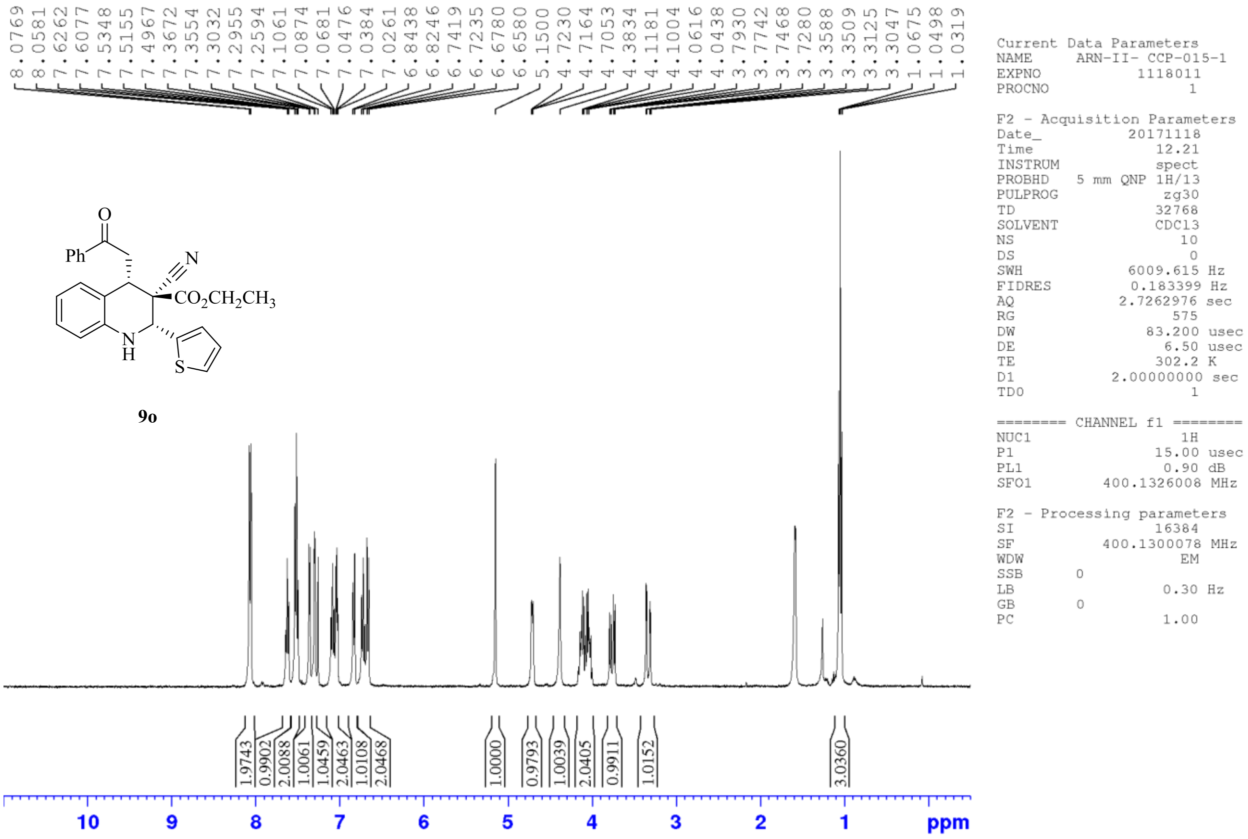

Racemate of ethyl 3-cyano-4-(2-oxo-2-phenylethyl)-2-(thiophen-2-yl)-1,2,3,4-tetrahydroquinoline-3-carboxylate (**9o)**

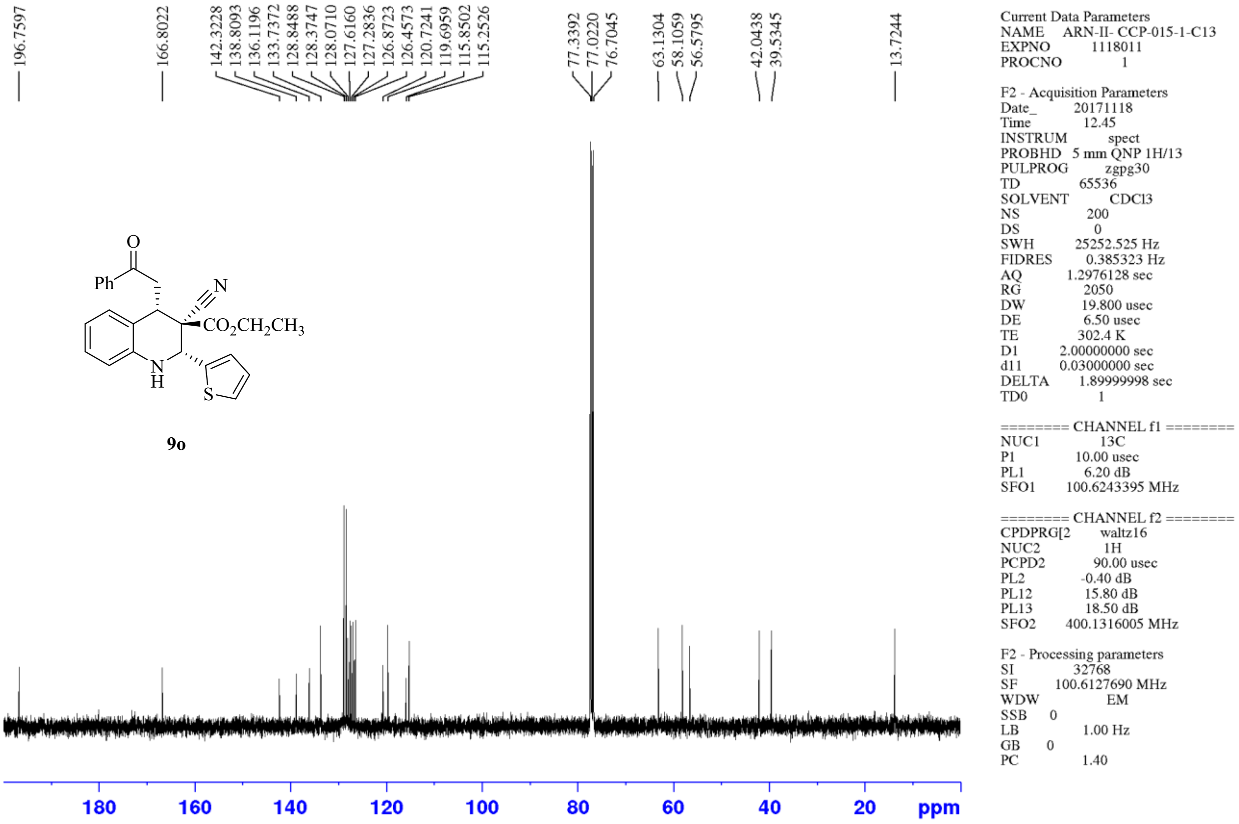

Racemate of ethyl 4-benzoyl-3-cyano-2-(3,5-dimethoxyphenyl)-1,2,3,4-tetrahydroquinoline-3-carboxylate (**9p**)

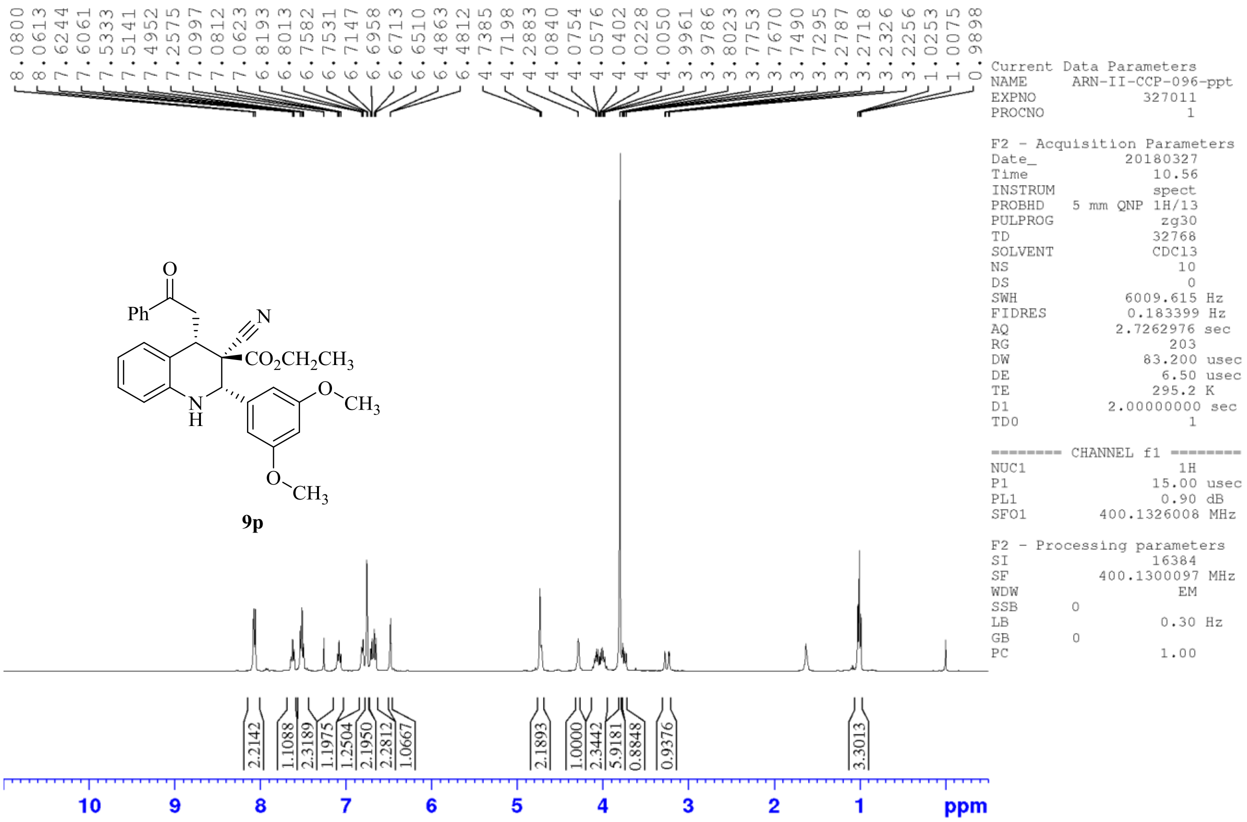

Racemate of ethyl 4-benzoyl-3-cyano-2-(3,5-dimethoxyphenyl)-1,2,3,4-tetrahydroquinoline-3-carboxylate (**9p**)

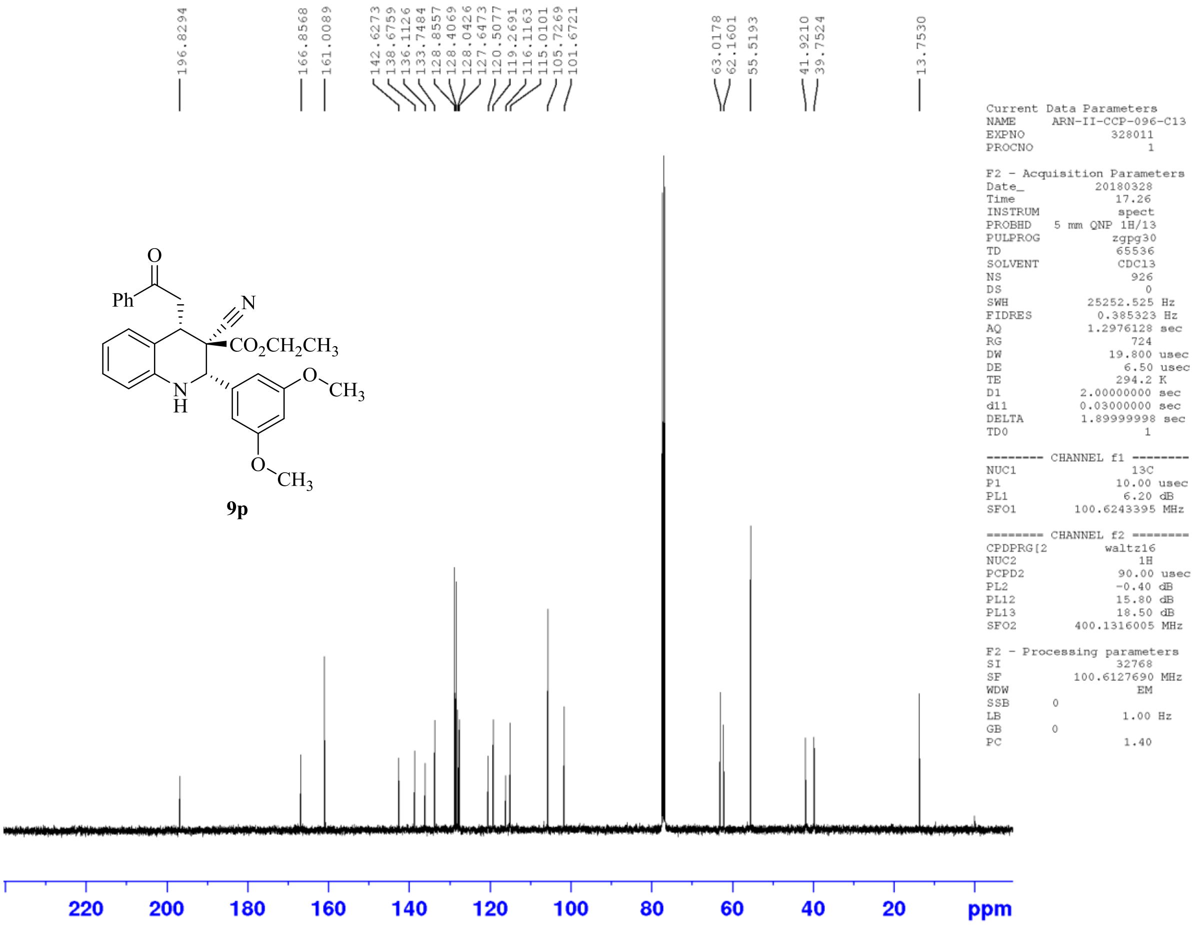

Racemate of ethyl 3-cyano-2-(4-nitrophenyl)-4-(2-oxo-2-phenylethyl)-1,2,3,4-tetrahydroquinoline-3-carboxylate (**9q**)

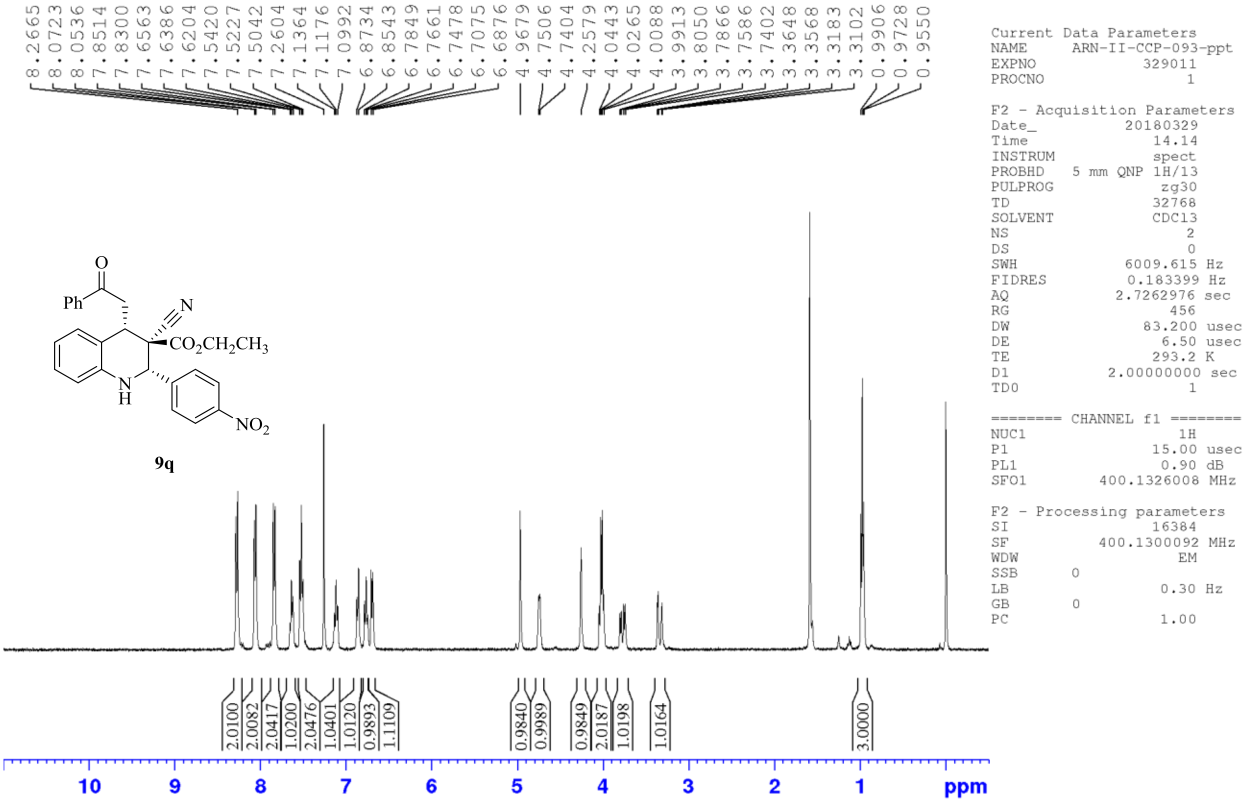

Racemic of ethyl 3-cyano-2-(4-nitrophenyl)-4-(2-oxo-2-phenylethyl)-1,2,3,4-tetrahydroquinoline-3-carboxylate (**9q**)

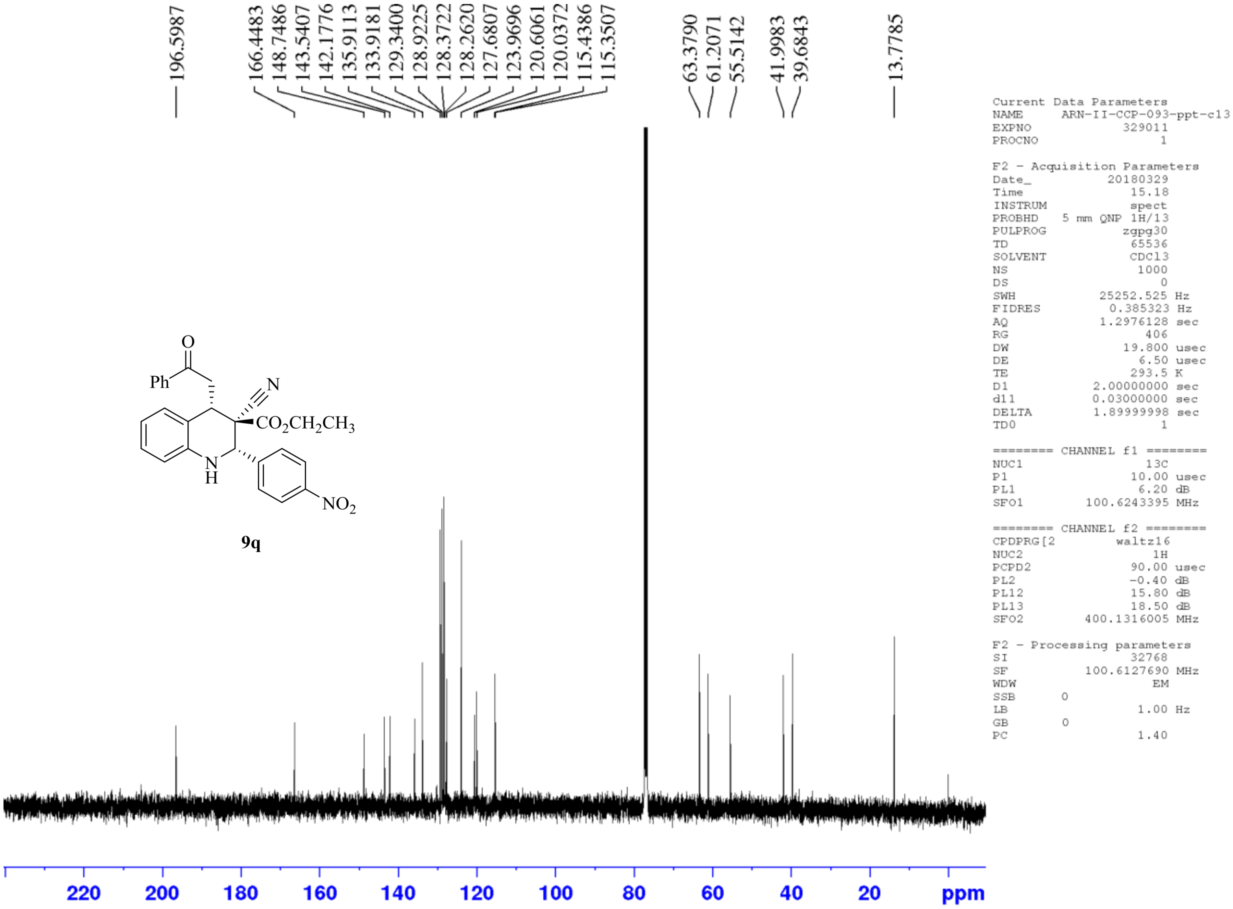

Racemic of 3-((ethylperoxy)-l2-methyl)-4-(2-oxo-2-phenylethyl)-2-(2,4,6-trimethoxyphenyl)-1,2,3,4-tetrahydroquinoline-3-carbonitrile (**9r**)

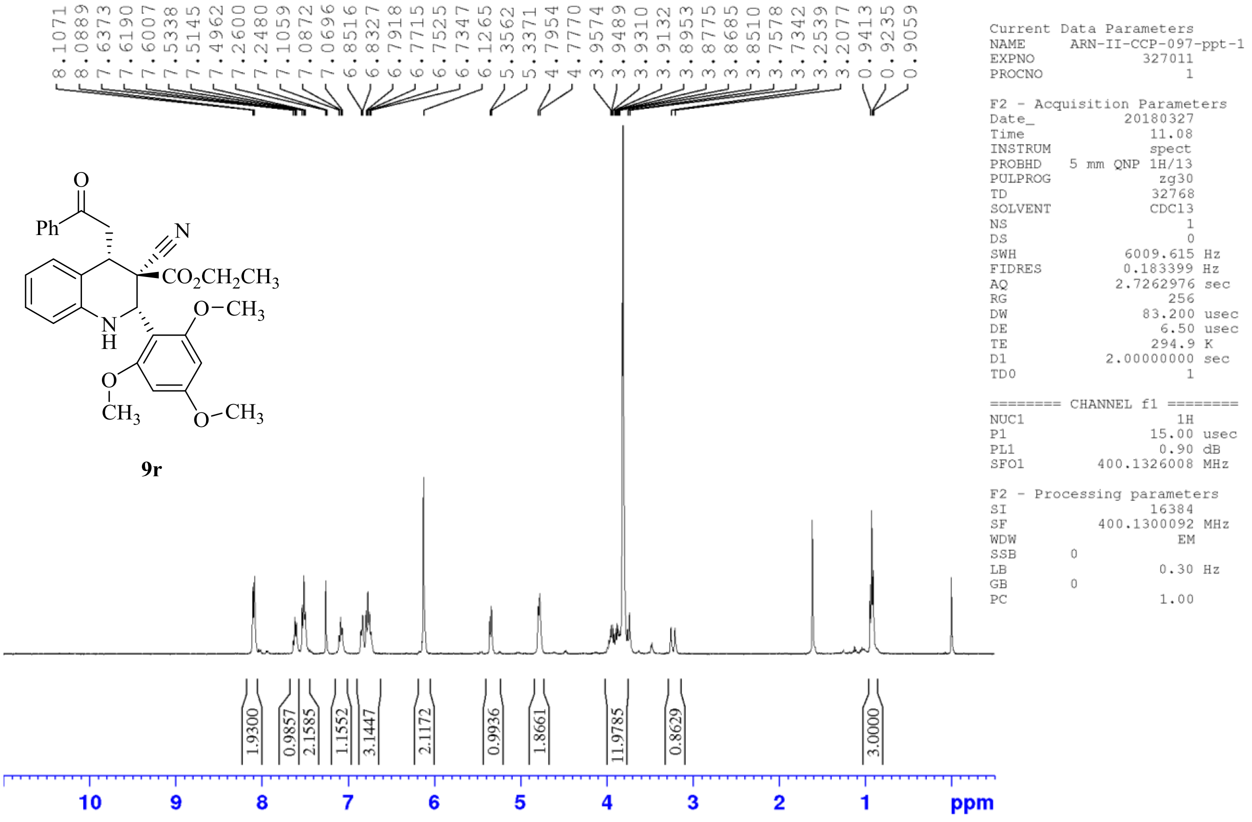

Racemic of 3-((ethylperoxy)-l2-methyl)-4-(2-oxo-2-phenylethyl)-2-(2,4,6-trimethoxyphenyl)-1,2,3,4-tetrahydroquinoline-3-carbonitrile (**9r**)

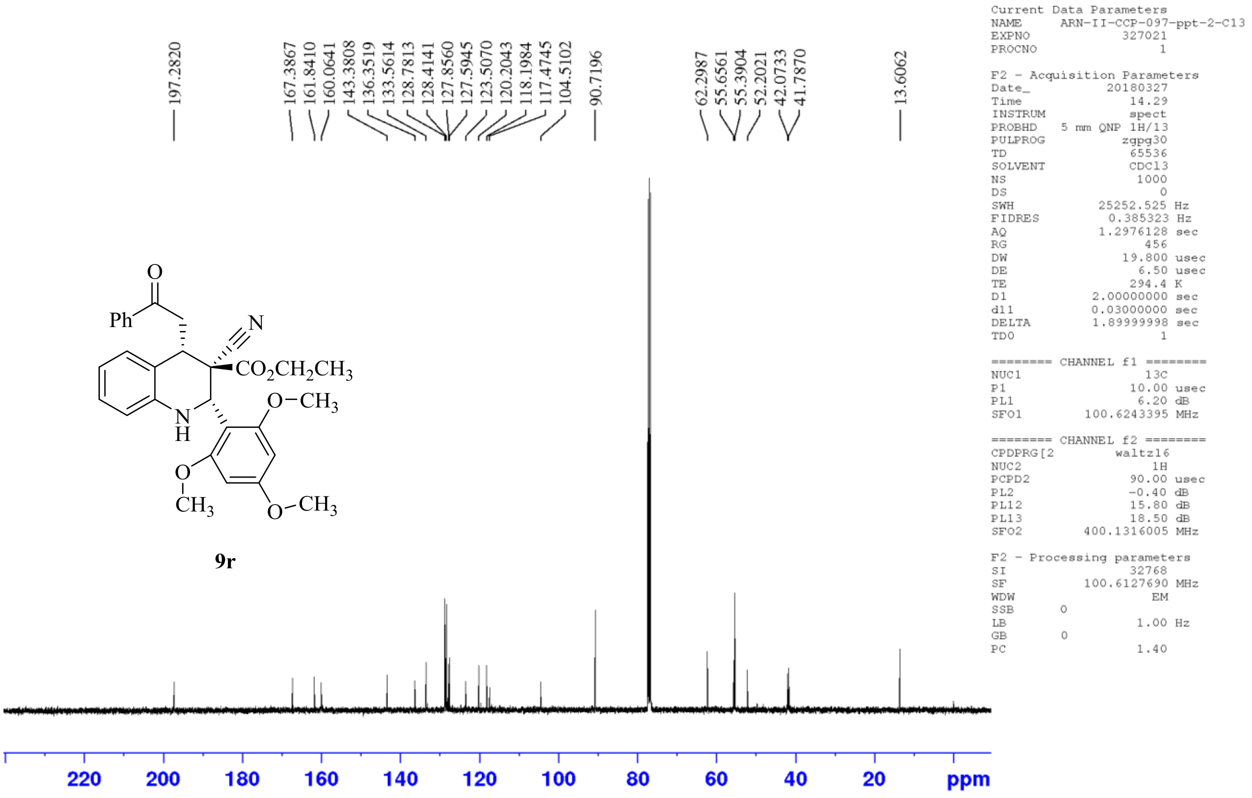

Supplement: RA-010-D0RA01264E-s002 [file RA-010-D0RA01264E-s002.pdf]
